# A statistical measure for the skewness of X chromosome inactivation based on family trios

**DOI:** 10.1186/s12863-018-0694-8

**Published:** 2018-12-05

**Authors:** Si-Qi Xu, Yu Zhang, Peng Wang, Wei Liu, Xian-Bo Wu, Ji-Yuan Zhou

**Affiliations:** 1 State Key Laboratory of Organ Failure Research, Ministry of Education and Guangdong Provincial Key Laboratory of Tropical Disease Research, Department of Biostatistics, School of Public Health, Southern Medical University, Guangzhou, China; 2 Guangdong Provincial Key Laboratory of Tropical Disease Research, Department of Epidemiology, School of Public Health, Southern Medical University, Guangzhou, China

**Keywords:** X chromosome inactivation, Skewing, Family trio, Ratio estimate

## Abstract

**Background:**

X chromosome inactivation (XCI) is an important gene regulation mechanism in females to equalize the expression levels of X chromosome between two sexes. Generally, one of two X chromosomes in females is randomly chosen to be inactivated. Nonrandom XCI (XCI skewing) is also observed in females, which has been reported to play an important role in many X-linked diseases. However, there is no statistical measure available for the degree of the XCI skewing based on family data in population genetics.

**Results:**

In this article, we propose a statistical approach to measure the degree of the XCI skewing based on family trios, which is represented by a ratio of two genotypic relative risks in females. The point estimate of the ratio is obtained from the maximum likelihood estimates of two genotypic relative risks. When parental genotypes are missing in some family trios, the expectation-conditional-maximization algorithm is adopted to obtain the corresponding maximum likelihood estimates. Further, the confidence interval of the ratio is derived based on the likelihood ratio test. Simulation results show that the likelihood-based confidence interval has an accurate coverage probability under the situations considered. Also, we apply our proposed method to the rheumatoid arthritis data from USA for its practical use, and find out that a locus, rs2238907, may undergo the XCI skewing against the at-risk allele. But this needs to be further confirmed by molecular genetics.

**Conclusions:**

The proposed statistical measure for the skewness of XCI is applicable to complete family trio data or family trio data with some paternal genotypes missing. The likelihood-based confidence interval has an accurate coverage probability under the situations considered. Therefore, our proposed statistical measure is generally recommended in practice for discovering the potential loci which undergo the XCI skewing.

**Electronic supplementary material:**

The online version of this article (10.1186/s12863-018-0694-8) contains supplementary material, which is available to authorized users.

## Background

Many human diseases are associated with genes on X chromosome, such as asthma, autoimmune diseases, cancers, some neurological and psychiatric diseases [1–5]. Most of these X-linked diseases often exhibit sex-specific patterns of susceptibility due to the difference in the number of copies of X chromosome between two sexes. Females have two copies of X chromosome whereas there is only one copy in males. To equalize the expression levels of X chromosome between sexes, dosage compensation is achieved by an important gene regulation mechanism in mammalian females, X chromosome inactivation (XCI), which results in expression silencing of one of two X chromosomes in females [6]. Up to 75% genes on X chromosome are subject to XCI, while there are about 15% escaping from inactivation and expressed from both X chromosomes, and the remaining 10% show variable inactivation patterns in different human cell lines [7].

During the process of XCI, one of two X chromosomes in females is chosen to be inactivated in a random way. This means that roughly 50% of cells in females have the paternal X chromosome expressed, while the others express the maternal one. Although random XCI occurs commonly, the XCI skewing also takes place in females, which is defined as the phenomenon of nonrandom inactivation that one of X chromosomes is selected to be silenced with a probability deviating from 50% [8]. Generally, the skewness of XCI is caused by a second selection mechanism. When the mutation on X chromosome affects the survival and proliferation of cells, the amount of cells carrying an active mutant X chromosome will become larger or smaller than that of cells with an active wild-type X chromosome, which thus leads to the skewness of XCI [9, 10]. Negative selection, where the mutation gives a growth disadvantage to cells, frequently happens in female carriers with X-linked diseases, such as mental retardation disorders, Wiskott-Aldrich syndrome and X-linked severe combined immunodeficiency [11–13]. On the other hand, when the mutation provides a growth advantage to cells, positive selection occurs and can result in some diseases, such as adrenoleukodystrophy and breast cancer [14, 15].

In genetic association studies on X chromosome, Clayton [16] first took XCI into consideration. Due to XCI, the genotypic effect of homozygous females can be treated the same as that of hemizygous males. Therefore, the genotypic scores are given to be 0, 1 and 2 corresponding to three genotypes at a diallelic locus on X chromosome in females, and 0 and 2 corresponding to two genotypes in males. However, Wang et al. [17] pointed out that such coding strategy only considers the situation of random XCI, but ignores the skewness of XCI and escape from XCI. To account for all possible situations of XCI, Wang et al. suggested that the genotypic score for the heterozygous females, denoted by *γ*, can be any possible values between 0 and 2. Under XCI, the value of *γ* reflects the degree of inactivation skewing, with *γ*/2 representing the proportion of cells having the mutant allele active. As such, *γ* = 1 stands for random XCI, while *γ* between 1 and 2 indicates the XCI skewing toward the mutant allele and *γ* between 0 and 1 denotes the XCI skewing against the mutant allele. For example, *γ* = 0.5 means that the skewness of XCI is against the mutant allele with 25% cells expressing the mutant allele and the other 75% cells expressing the normal allele. On the other hand, in molecular genetics, the XCI skewing pattern can be identified by assays taking advantage of differential methylation between the active and inactive X chromosomes or mRNA transcription in cells [18–20]. However, since the XCI pattern always varies among cell lines [21, 22], these assays, which usually use cells from only a few tissues to investigate the XCI patterns, cannot present the status of the whole body [10]. Further, there is no statistical measure available for detecting the XCI skewing pattern in population genetics as yet.

Therefore, in this article, we give the expression of *γ* for family trios with both parents and one affected daughter in the presence of association between the disease and genotypes. In fact, *γ* is a function of two genotypic relative risks (GRRs) in females. In addition, we obtain the point estimate of *γ* from the maximum likelihood estimates (MLEs) of the GRRs. When parental genotypes are missing in some family trios, the expectation-conditional-maximization (ECM) algorithm [23] is used to obtain the corresponding MLEs. Further, the confidence interval (CI) of *γ* is derived based on a likelihood ratio test (LRT). Finally, simulation study is conducted to investigate the performance of our proposed method. The simulation results show that the proposed likelihood-based CI has an accurate coverage probability under the situations considered. For practical use, we apply our proposed method to the rheumatoid arthritis (RA) data from USA.

## Methods

### Notations

Consider an X-linked diallelic locus with mutant allele *A* and normal allele *a*. Let *p*_*m*_ and *q*_*m*_ = 1 − *p*_*m*_ denote the allele frequencies of *A* and *a* in males, respectively. Suppose that *p*_*f*_ is the allele frequency of *A* and *ρ* is the inbreeding coefficient in females. Then, the frequencies of genotypes *aa*, *Aa* and *AA* in females are respectively *g*_0_ = (1 − *p*_*f*_)^2^ + *ρp*_*f*_(1 − *p*_*f*_), *g*_1_ = 2(1 − *ρ*)*p*_*f*_(1 − *p*_*f*_) and ${g}_2={p}_f^2+\rho {p}_f\left(1-{p}_f\right)$. Note that Hardy-Weinberg equilibrium holds in the population under study when *ρ* = 0 and *p*_*m*_ = *p*_*f*_. Let *f*_0_, *f*_1_ and *f*_2_ respectively represent the penetrances in females with genotypes *aa*, *Aa* and *AA*. The GRRs in females are defined as *λ*_1_ = *f*_1_/*f*_0_ and *λ*_2_ = *f*_2_/*f*_0_.

### Relationship between penetrances and XCI skewing in females

Let the genotypic scores be 0, *γ* and 2 corresponding to females with genotypes *aa*, *Aa* and *AA*, respectively, where *γ* ∈ [0, 2] represents the XCI skewing pattern. We assume that a generalized genetic model holds [24, 25], which is defined as *f*_0_ ≤ *f*_1_ ≤ *f*_2_ (i.e., 1 ≤ *λ*_1_ ≤ *λ*_2_) with at least one inequality being strict, in the presence of association between the disease and genotypes in females. If *f*_1_ is unknown, then it can be expressed as a function of *γ*, denoted by *f*_1_(*γ*). To derive the expression of *f*_1_(*γ*), let ${f}_1^{\prime}\left(\gamma \right)$ be the first order derivative of *f*_1_(*γ*) with respect to *γ*. As such, *f*_1_(*γ*) can be approximated by a first order Taylor expansion around *γ* = 1 as follows,

1 $$ {f}_1\left(\gamma \right)\approx {f}_1(1)+{f}_1^{\prime }(1)\left(\gamma -1\right). $$

On the other hand, when the XCI skewing is completely against *A*, we have *γ*=0 and *f*_1_ = *f*_0_. So, from Eq. (1), ${f}_0={f}_1(0)\approx {f}_1(1)-{f}_1^{\prime }(1)$. Similarly, when the XCI skewing is completely toward *A*, we have *γ*=2 and *f*_1_ = *f*_2_. Then ${f}_2={f}_1(2)\approx {f}_1(1)+{f}_1^{\prime }(1)$. Hence, *f*_1_(1) = (*f*_2_ + *f*_0_)/2 and ${f}_1^{\prime }(1)=\left({f}_2-{f}_0\right)/2$. Therefore, Eq. (1) turns to be

2 $$ {f}_1\left(\gamma \right)\approx {f}_0+\frac{\gamma \left({f}_2-{f}_0\right)}{2}. $$

From Eq. (2), we notice that *f*_1_ is around the midpoint between *f*_0_ and *f*_2_ under random XCI (i.e., *γ*=1). Actually, Eq. (2) means that the penetrance for heterozygous females is approximately linear in the genotypic score *γ* around *γ*=1.

Further, if *f*_1_ is known, then we can obtain *γ* from Eq. (2) as follows, which is a function of *f*_0_, *f*_1_ and *f*_2_, or *λ*_1_ and *λ*_2_,

$$ \gamma \approx \frac{2\left({f}_1-{f}_0\right)}{f_2-{f}_0}=\frac{2\left({\lambda}_1-1\right)}{\lambda_1-1}. $$

Note that the value of *γ* attains its maximum (*γ* = 2) when *λ*_1_ = *λ*_2_ ≠ 1, and *γ* = 0 when *λ*_1_ = 1 and *λ*_2_ ≠ 1. Assume that ${\widehat{\lambda}}_1$ and ${\widehat{\lambda}}_2$ are the MLEs of *λ*_1_ and *λ*_2_, respectively. Then, the point estimate of *γ*, $\widehat{\gamma}$, can be obtained by $2\left({\widehat{\lambda}}_1-1\right)/\left({\widehat{\lambda}}_2-1\right)$.

### MLEs of *λ*_1_ and *λ*_2_ using family trios without missing genotypes

Here we only include the trios with affected daughters in the analysis. Male offspring are not investigated because they are not informative of *λ*_1_ and *λ*_2_. Firstly, we consider complete family trios, each with both typed parents and an affected typed daughter. Let *F*, *M* and *C* represent the numbers of allele *A* in father, mother and daughter, respectively, and *D* denote that the daughter is affected. Eight possible types of *FMC* (i.e., 000, 010, 011, 021, 101, 111, 112 and 122) together with the corresponding probabilities *P*(*FM*), *P*(*C*| *FM*) and *P*(*FMC*| *D*) are shown in Table 1. Let Ω be the set of the eight possible types of *FMC* listed in Table 1 and then *P*(*FMC*| *D*) is derived as

3 $$ P\left( FM C\left|D\right.\right)=\frac{P(FM)P\left(C\left| FM\right.\right)P\left(D\left|C\right.\right)}{\sum_{F^{\prime }{M}^{\prime }{C}^{\prime}\in \Omega}P\left({F}^{\prime }{M}^{\prime}\right)P\left({C}^{\prime}\left|{F}^{\prime }{M}^{\prime}\right.\right)P\left(D\left|{C}^{\prime}\right.\right)}. $$

**Table 1:** Eight types of possible family trios and the corresponding probabilities

*FMC*	*P*(*FM*)	*P*(*C*| *FM*)	*P*(*FMC*| *D*)
000	*q*_*m*_ *g*_0_	1	*q*_*m*_*g*_0_/*R*
010	*q*_*m*_ *g*_1_	0.5	0.5*q*_*m*_*g*_1_/*R*
011	*q*_*m*_ *g*_1_	0.5	0.5*q*_*m*_*g*_1_*λ*_1_/*R*
021	*q*_*m*_ *g*_2_	1	*q*_*m*_*g*_2_*λ*_1_/*R*
101	*p*_*m*_ *g*_0_	1	*p*_*m*_*g*_0_*λ*_1_/*R*
111	*p*_*m*_ *g*_1_	0.5	0.5*p*_*m*_*g*_1_*λ*_1_/*R*
112	*p*_*m*_ *g*_1_	0.5	0.5*p*_*m*_*g*_1_*λ*_2_/*R*
122	*p*_*m*_ *g*_2_	1	*p*_*m*_*g*_2_*λ*_2_/*R*

Equation (3) holds when the disease status of a daughter is only related to her own genotype, and *P*(*C*| *FM*) is determined by Mendelian transmission, which is equal to 0.5 for heterozygous mother and 1 otherwise. Assume that *P*(*FM*) = *P*(*F*)*P*(*M*), and divide the numerator and denominator of Eq. (3) by *f*_0_. Then, *P*(*FMC*| *D*) for each trio type can be written as the last column in Table 1, where *R* = *q*_*m*_*g*_0_ + 0.5*q*_*m*_*g*_1_(1 + *λ*_1_) + *q*_*m*_*g*_2_*λ*_1_ + *p*_*m*_*g*_0_*λ*_1_ + 0.5*p*_*m*_*g*_1_(*λ*_1_ + *λ*_2_) + *p*_*m*_*g*_2_*λ*_2_. The detailed derivation of *P*(*FMC*| *D*) in Table 1 is given in Additional file 1: Appendix A.

Since we find that it is more convenient to directly estimate *g*_0_ and *g*_1_ rather than *ρ* and *p*_*f*_, we let *θ*= (*p*_*m*_, *g*_0_, *g*_1_, *λ*_1_, *λ*_2_)^*T*^ be the parameter vector of interest. As such, the log-likelihood function of the observed data conditional on the daughter being affected is given by

$$ \ln L\left(\theta \right)=\sum \limits_{FMC\in \Omega}{n}_{FMC}\ln P\left( FMC\left|D\right.\right), $$

where *n*_*FMC*_ is the number of the family trios of type *FMC*. To obtain the MLE of *θ*, numerical methods, such as Newton-Raphson algorithm (by using “maxLik” package in R software [26]) and the ECM algorithm introduced later, are applied. We choose the initial values of *p*_*m*_, *g*_0_, *g*_1_, *λ*_1_ and *λ*_2_ as follows: ${\widehat{p}}_m^{(0)}=\#\left(F=1\right)/\#\left(F\in \left\{0,1\right\}\right),$ ${\widehat{g}}_0^{(0)}=\#\left(M=0\right)/\#\left(M\in \left\{0,1,2\right\}\right)$, ${\widehat{g}}_1^{(0)}=\#\left(M=1\right)/\#\left(M\in \left\{0,1,2\right\}\right),$ ${\widehat{\lambda}}_1^{(0)}={n}_{011}/{n}_{010}$ and ${\widehat{\lambda}}_2^{(0)}=\left({n}_{011}{n}_{112}\right)/\left({n}_{010}{n}_{111}\right)$, where # denotes the counting measure. The details about the choice of these initial values are shown in Additional file 1: Appendix B.

### MLEs of *λ*_1_ and *λ*_2_ using family trios with parental genotypes missing

It is common that parental genotypes are missing in some family trios. For trios with paternal or maternal genotype missing, we call them “mother-daughter pairs” or “father-daughter pairs”, denoted by *MC* and *FC*, respectively. Thus, *MC* takes possible genotypes from Ω_*MC*_={00, 01, 10, 11, 12, 21, 22}, and *FC* takes possible genotypes from Ω_*FC*_={00, 01, 11, 12}. As for trios with both parental genotypes missing, we refer to them as “single daughters”. The probabilities of the mother-daughter pair *MC*, father-daughter pair *FC* and single daughter *C* given the daughter being affected are respectively$P\left( MC|D\right)={\sum}_{F\in \left\{0,1\right\}}P\left( FMC|D\right)$, $P\left( FC|D\right)={\sum}_{M\in \left\{0,1,2\right\}}P\left( FMC|D\right)$ and $P\left(C|D\right)={\sum}_{F\in \left\{0,1\right\}}{\sum}_{M\in \left\{0,1,2\right\}}P\left( FMC|D\right)$, where *P*(*FMC*| *D*) are given in Table 1.

Suppose that we collect *n*_*FMC*_ family trios of type *FMC*, *n*_1*m*, *MC*_ mother-daughter pairs of type *MC*, *n*_1*f*, *FC*_ father-daughter pairs of type *FC* and *n*_0, *C*_ single daughters of type *C*, where the subscripts 1 *m*, 1*f* and 0 respectively mean that each trio has only a mother, only a father and no parents. Then, the log-likelihood function of the observed data is

4 $$ {\displaystyle \begin{array}{l}\ln L\left(\theta \right)=\sum \limits_{FMC\in \Omega}{n}_{FMC}\ln P\left( FMC\left|D\right.\right)+\sum \limits_{MC\in {\Omega}_{MC}}{n}_{1m, MC}\ln P\left( MC\left|D\right.\right)\\ {}+\sum \limits_{FC\in {\Omega}_{FC}}{n}_{1f, FC}\ln P\left( FC\left|D\right.\right)+\sum \limits_{C\in \left\{0,1,2\right\}}{n}_{0,C}\ln P\left(C\left|D\right.\right).\end{array}} $$

Let *N*_2_, *N*_1*m*_, *N*_1*f*_ and *N*_0_ respectively be the numbers of family trios, mother-daughter pairs, father-daughter pairs and single daughters. Then, ${N}_2={\sum}_{FMC\in \Omega}{n}_{FMC}$, ${N}_{1m}={\sum}_{MC\in {\Omega}_{MC}}{n}_{1m, MC},{N}_{1f}={\sum}_{FC\in {\Omega}_{FC}}{n}_{1f, FC}$, ${N}_0={\sum}_{C\in \left\{0,1,2\right\}}{n}_{0,C}$ and the total sample size *N* = *N*_2_ + *N*_1*m*_ + *N*_1*f*_ + *N*_0_.

Since it is not so easy to obtain the MLE of *θ* from the above observed log-likelihood function (4), the ECM algorithm will be employed. Assume that ${n}_{1m, MC}={\sum}_{F\in \left\{0,1\right\}}{z}_{1m, FMC}$, ${n}_{1f, FC}={\sum}_{M\in \left\{0,1,2\right\}}{z}_{1f, FMC}$ and *n*_0, *C*_= ${\sum}_{F\in \left\{0,1\right\}}{\sum}_{M\in \left\{0,1,2\right\}}{z}_{0, FMC}$, where *z*_1*m*, *FMC*_, *z*_1*f*, *FMC*_ and *z*_0, *FMC*_ are the unobserved numbers of trios *FMC* for mother-daughter pairs *MC*, father-daughter pairs *FC* and single daughters *C*, respectively (see Additional file 1: Tables S1-S3). Then, the log-likelihood function for the complete data (*n*_*FMC*_, *z*_1*m*, *FMC*_, *z*_1*f*, *FMC*_, *z*_0, *FMC*_) can be written as

$$ \ln {L}_C\left(\theta \right)=\sum \limits_{FMC\in \Omega}\left({n}_{FMC}+{z}_{1m, FMC}+{z}_{1f, FMC}+{z}_{0, FMC}\right)\ln P\left( FMC|D\operatorname{}\right). $$

The following ECM algorithm contains one E-step and five CM-steps at each iteration. In the E-step at iteration (*k* + 1), we obtain the conditional expectation of ln*L*_*C*_(*θ*) with respect to the conditional distributions of *z*_1*m*, *FMC*_, *z*_1*f*, *FMC*_ and *z*_0, *FMC*_ given *n*_1*m*, *MC*_, *n*_1*f*, *FC*_ and *n*_0, *C*_, respectively. *z*_1*m*, *FMC*_ ∣ *n*_1*m*, *MC*_, *z*_1*f*, *FMC*_ ∣ *n*_1*f*, *FC*_ and *z*_0, *FMC*_ ∣ *n*_0, *C*_ follow the binomial distributions with respective success probabilities *P*(*F*| *MC*, *D*), *P*(*M*| *FC*, *D*) and *P*(*FM*| *C*, *D*). Thus, the *Q* function is given by

5 $$ Q\left(\theta |{\widehat{\theta}}^{(k)}\right)=\sum \limits_{FMC\in \Omega}\left[{n}_{FMC}+{E}_{{\widehat{\theta}}^{(k)}}\Big({z}_{1m, FMC}|{n}_{1m, MC}\right)+{E}_{{\widehat{\theta}}^{(k)}}\left({z}_{1f, FMC}\left|{n}_{1f, FC}\right)+{E}_{{\widehat{\theta}}^{(k)}}\left({z}_{0, FMC}|{n}_{0,C}\right)\right]\ \ln P\left( FMC|D\right), $$

$\mathrm{where}\kern0.5em {\hat{\theta}}^{(k)}={\left({\hat{p}}_m^{(k)},{\hat{g}}_0^{(k)},{\hat{g}}_1^{(k)},{\hat{\lambda}}_1^{(k)},{\hat{\lambda}}_2^{(k)}\right)}^T$ is the MLE of *θ* at iteration *k*,

$$ {E}_{{\widehat{\theta}}^{(k)}}\left({z}_{1m, FMC}|{n}_{1m, MC}\right)={n}_{1m, MC}P\left(F| MC,D;{\widehat{\theta}}^{(k)}\right)={n}_{1m, MC}\frac{P\left( FMC|D;{\widehat{\theta}}^{(k)}\right)}{\sum \limits_{F^{\prime}\in \left\{0,1\right\}}P\left({F}^{\prime } MC|D;{\widehat{\theta}}^{(k)}\right)}, $$

$$ {E}_{{\widehat{\theta}}^{(k)}}\left({z}_{1f, FMC}|{n}_{1f, FC}\right)={n}_{1f, FC}P\left(M| FC,D;{\widehat{\theta}}^{(k)}\right)={n}_{1f, FC}\frac{P\left( FMC|D;{\widehat{\theta}}^{(k)}\right)}{\sum \limits_{M^{\prime}\in \left\{0,1,2\right\}}P\left(F{M}^{\prime }C|D;{\widehat{\theta}}^{(k)}\right)} $$

and

$$ {E}_{{\widehat{\theta}}^{(k)}}\Big({z}_{0, FMC}\left|{n}_{0,C}\right)={n}_{0,C}P\left( FM|C,D;{\widehat{\theta}}^{(k)}\right)={n}_{0,C}\frac{P\left( FM C|D;{\widehat{\theta}}^{(k)}\right)}{\sum \limits_{F^{\prime}\in \left\{0,1\right\}}\sum \limits_{M^{\prime}\in \left\{0,1,2\right\}}P\left({F}^{\prime }{M}^{\prime }C|D;{\widehat{\theta}}^{(k)}\right)}. $$

In the CM-steps, the *Q* function is maximized with respect to each of components of *θ* in turn, with the others fixed at their previous values. The MLE of *θ* at iteration (*k* + 1) are given in Additional file 1: Appendix B. The initial value of *θ* is obtained only based on *N*_2_ complete family trios when *N*_2_ ≠ 0 (see Additional file 1: Appendix B). However, when *N*_2_ = 0, we estimate the initial values of *λ*_1_ and *λ*_2_ by replacing unknown *n*_010_, *n*_011_, *n*_111_ and *n*_112_ values in ${\widehat{\lambda}}_1^{(0)}={n}_{011}/{n}_{010}$ and ${\widehat{\lambda}}_2^{(0)}=\left({n}_{011}{n}_{112}\right)/\left({n}_{010}{n}_{111}\right)$ by their respective conditional expectations (see Additional file 1: Tables S1-S3). For example, *n*_011_ is replaced by

$$ E\left({z}_{1m,011}|{n}_{1m,11}\right)+E\left({z}_{1f,011}|{n}_{1f,01}\right)+E\left({z}_{\mathrm{0,011}}|{n}_{0,1}\right) $$

$$ ={n}_{1m,11}{\widehat{q}}_m^{(0)}+{n}_{1f,01}\bullet \frac{0.5{\widehat{g}}_1^{(0)}}{0.5{\widehat{g}}_1^{(0)}+{\widehat{g}}_2^{(0)}}+{n}_{0,1}\bullet \frac{0.5{\widehat{q}}_m^{(0)}{\widehat{g}}_1^{(0)}}{{\widehat{p}}_m^{(0)}{\widehat{g}}_0^{(0)}+0.5{\widehat{g}}_1^{(0)}+{\widehat{q}}_m^{(0)}{\widehat{g}}_2^{(0)}}, $$

where ${\widehat{p}}_m^{(0)}$, ${\widehat{q}}_m^{(0)}=1-{\widehat{p}}_m^{(0)}$, ${\widehat{g}}_0^{(0)}$, ${\widehat{g}}_1^{(0)}$ and ${\widehat{g}}_2^{(0)}=1-{\widehat{g}}_0^{(0)}-{\widehat{g}}_1^{(0)}$are the initial values of *p*_*m*_, *q*_*m*_, *g*_0_, *g*_1_ and *g*_2_, respectively. The details about the choice of these initial values are shown in Additional file 1: Appendix B. Given the initial value of *θ*, the steps mentioned above continue until the convergence criterion is satisfied. For example, the absolute differences between the estimates of the parameters at two consecutive iterations are all less than 10^−7^. In addition, note that the ECM algorithm still works when there are no missing genotypes in all the family trios. However, it contains only the CM steps in this situation and can be regarded as a special case of the cyclic coordinate ascent method, which is simple and stable [23].

### Confidence interval of *γ* based on likelihood method

To obtain the CI, we first construct a LRT for testing the null hypothesis *H*_0_: *γ* = *γ*_0_ as follows,

$$ \mathrm{LRT}=2\ln \frac{L\left(\widehat{\theta}\right)}{L\left({\overset{\sim }{\theta}}_0\right)}, $$

where $\widehat{\theta}=$ (${\widehat{p}}_m$, ${\widehat{g}}_0$, ${\widehat{g}}_1$, ${\widehat{\lambda}}_1$, ${\widehat{\lambda}}_2$)^*T*^ is the MLE of *θ* under *H*_1_. Let *θ*_0_= (*p*_*m*_, *g*_0_, *g*_1_, *λ*_2_)^*T*^ be the parameter vector under *H*_0_ with *γ*_0_ = 2(*λ*_1_ − 1)/(*λ*_2_ − 1) (i.e., *λ*_1_ = *γ*_0_(*λ*_2_ − 1)/2 + 1), and then ${\overset{\sim }{\theta}}_0=$ (${\overset{\sim }{p}}_m$, ${\overset{\sim }{g}}_0$, ${\overset{\sim }{g}}_1$, ${\overset{\sim }{\lambda}}_2$)^*T*^ denotes the MLE of *θ*_0_. The choice of the initial value of *θ*_0_ and the solution of ${\overset{\sim }{\theta}}_0$ using family trios with missing parental genotypes is given in Additional file 1: Appendix B. The LRT asymptotically follows a chi-square distribution with the degree of freedom being one (i.e., ${\chi}_1^2$).

At the significance level α, the 100(1 − α)% confidence interval of *γ* based on the LRT is

$$ \left\{\gamma :\mathrm{LRT}\left(\gamma \right)<\kern0.5em {\chi}_{1-\alpha, 1}^2\right\}, $$

and the confidence limits are the values that satisfy

6 $$ \mathrm{LRT}\left(\gamma \right)={\chi}_{1-\alpha, 1}^2. $$

Note that there is no closed-form solution of Eq. (6). Thus, numerical method is applied, such as functions from “rootSolve” package in R software [26]. Let *γ*_*L*_ and *γ*_*U*_ be two unequal roots of Eq. (6) with *γ*_*L*_ < *γ*_*U*_. Generally, the 100(1 − α)% CI of *γ* would be (*γ*_*L*_, *γ*_*U*_). However, since the true value of *γ* is bounded in [0, 2], the original estimated CI of *γ* will be truncated by [0, 2] if necessary. As such, the ultimate CI of *γ* is (*γ*_*L*_, *γ*_*U*_)∩[0, 2], which is easier to be interpreted than the origin CI.

### Discontinuity problem of confidence interval of *γ*

Note that *γ* is a ratio, so like other ratio estimates [27], we find that the proposed CI may consist of two disjoint intervals, such as [0, 0.03)∪(0.59, 2]. In this article, this kind of CIs is referred to as “discontinuous CI” for convenience. Let’s take a close look at this discontinuity problem by the following example. Consider the situation of (*n*_000_, *n*_010_, *n*_011_, *n*_021_, *n*_101_, *n*_111_, *n*_112_, *n*_122_)=(191, 89, 112, 54, 114, 59, 62, 19). Then, $\widehat{\gamma}$ is 1.92 and two roots of Eq. (6) are 0.03 and 0.59, respectively. If the CI is set to be (0.03, 0.59) normally, to our surprise, $\widehat{\gamma}$ is located outside this CI. When testing the null hypothesis *H*_0_: *γ* = *γ*_0_, we find that *γ*_0_ taking values between 0.03 and 0.59 is rejected by the LRT. This means that the interval (0.03, 0.59) is actually a rejection region of the corresponding LRT rather than an acceptance region. Hence, the CI of *γ* turns to be (−∞, 0.03)∪(0.59,+∞), and [0, 0.03)∪(0.59, 2] after being truncated. The discontinuous CI may occur when the denominator of the ratio is close to zero (i.e., *λ*_2_ is close to 1 in this article) [28]. In fact, when *λ*_2_ = 1, we assume that we cannot obtain information on the XCI skewing pattern according to the CI of *γ*. This is because our proposed method measures XCI skewness in the presence of association between the disease and genotypes (i.e., *λ*_2_ ≠ 1). On the other hand, although these discontinuous CIs are considered to be uninformative and are difficult to be interpreted, there is no satisfactory “objective” method for dealing with this problem well [27].

### Simulation settings

To assess the performance of the proposed method, we conduct the following simulation study. The sample size *N* is taken to be 700, which is close to that of RA data (757 pedigrees) [29]. We consider six different combinations of (*N*_2_, *N*_1*m*_, *N*_1*f*_, *N*_0_), which are referred to as six “missing patterns” (MP1–MP6) for convenience and are shown in Table 2. When the missing pattern changes from MP1 to MP4, the number of case-parents trios *N*_2_ decreases and the number of single daughters *N*_0_ increases with *N*_1*m*_ = *N*_1*f*_. For MP5 and MP6, the number of mother-daughter pairs *N*_1*m*_ is different from that of father-daughter pairs *N*_1*f*_ with *N*_2_ = *N*_0_. In addition, (*p*_*m*_, *p*_*f*_) is set to be (0.30, 0.30), (0.25, 0.30), (0.30, 0.25), (0.20, 0.20), (0.15, 0.20) and (0.20, 0.15), and we assume that *ρ*=0 and 0.05, and *λ*_2_=1.5 and 2. *λ*_1_ is calculated from *λ*_1_ = *γ*(*λ*_2_ − 1)/2 + 1, where *γ* varies from 0 to 2 in increments of 0.5. Given *p*_*m*_, *p*_*f*_, *ρ*, *λ*_2_ and *γ*, *N*_2_ case-parents trios are randomly generated from a multinomial distribution with probabilities *P*(*FMC*| *D*) shown in Table 1. Similarly, *N*_1*m*_ mother-daughter pairs, *N*_1*f*_ father-daughter pairs and *N*_0_ single daughters are randomly drawn from the multinomial distributions with probabilities $P\left( MC|D\right)={\sum}_{F\in \left\{0,1\right\}}P\left( FMC|D\right)$, $P\left( FC|D\right)={\sum}_{M\in \left\{0,1,2\right\}}P\left( FMC|D\right)$ and $P\left(C|D\right)={\sum}_{F\in \left\{0,1\right\}}{\sum}_{M\in \left\{0,1,2\right\}}P\left( FMC|D\right)$, respectively. The simulations are based on *k*=10,000 replicates and 5% significance level.

**Table 2:** Six simulation settings for different combinations of (*N*_2_, *N*_1*m*_, *N*_1*f*_, *N*_0_) with total sample size *N* being fixed at 700

MP	*N*_2_	*N*_1 *m*_	*N*_1 *f*_	*N*_0_
1	700	0	0	0
2	350	100	100	150
3	0	200	200	300
4	0	100	100	500
5	100	400	100	100
6	100	100	400	100

We assess the statistical properties of the CI by the following indexes. Let the coverage probability (CP) be the proportion that the CI contains the true value of *γ* among *k* replicates. Note that under *H*_0_: *γ* = *γ*_0_, the estimated type І error rate of the LRT is 1−CP. ML and MR denote the left tail error and the right tail error (missing the true value of *γ*), respectively, with ML $=\#\left[\left(\gamma <{\gamma}_L\right)\cap \left({\gamma}_L\le \widehat{\gamma}\le {\gamma}_U\right)\right]/k$ and MR $=\#\left[\left(\gamma >{\gamma}_U\right)\cap \left({\gamma}_L\le \widehat{\gamma}\le {\gamma}_U\right)\right]/k$. Further, we use ML/(ML + MR) to measure the balance of ML and MR, which will be close to 0.5 when the balance is achieved. Notice that we do not consider those discontinuous CIs when calculating ML and MR, since we cannot distinguish between the left side and the right side of the discontinuous CIs. As such, we use DP$=1-\#\left({\gamma}_L\le \widehat{\gamma}\le {\gamma}_U\right)/k$ to represent the proportion of the discontinuous CIs among *k* replicates. In addition, note that the distribution of a ratio is not necessarily symmetric [30, 31], and the median can be always used to estimate the central tendency of a skewed distribution better than the mean [32]. So, we give the median of the point estimates of *γ* over *k* replicates under each simulation scenario. Further, for simulating the power of the LRT, we fix *γ*_0_ at 0, 1 and 2. Finally, we also compare the simulation results under MP1 (consisting of only 700 family trios with both parents) based on the ECM algorithm with those based on the Newton-Raphson algorithm. It is found that the results of the two algorithms are almost consistent with each other (data not shown for brevity). Therefore, we only give the simulation results on the basis of the ECM algorithm in the following section.

## Results

### Simulation results

Table 3 lists the estimated CP, ML/(ML + MR) and DP of the likelihood-based CI of *γ* against MP and *γ* with *ρ*=0, *λ*_2_=1.5, and (*p*_*m*_, *p*_*f*_) being (0.30, 0.30), (0.25, 0.30) and (0.30, 0.25). It is shown in the table that the CP is around 95% under the situations considered. On the other hand, we find that ML/(ML + MR) and DP appear not to be greatly affected by (*p*_*m*_, *p*_*f*_). However, the value of *γ* has strong effect on ML/(ML + MR) and DP. When *γ* takes values on the boundary (i.e., 0 and 2), ML/(ML + MR) always stays close to 1 and 0, respectively, which indicates extreme imbalance of two tail errors. DP increases as *γ* gets close to the boundary. Also, the missing pattern has great influence on both ML/(ML + MR) and DP. When the missing pattern varies from MP1 to MP4, where the number of case-parents trios decreases and that of single daughters increases, ML/(ML + MR) becomes more and more far away from 0.5 and DP sharply increases. We also find that under MP5, where the number of mother-daughter pairs is larger than that of father-daughter pairs, ML/(ML + MR) is a little closer to 0.5 and DP becomes smaller compared to those under MP6.

**Table 3:** Statistical properties of likelihood-based confidence interval of *γ* against missing pattern (MP) and *γ* with *ρ*=0, *λ*_2_=1.5, and (*p*_*m*_, *p*_*f*_) being (0.30, 0.30), (0.25, 0.30) and (0.30, 0.25)^a^

		(*p*_*m*_, *p*_*f*_) = (0.30, 0.30)			(*p*_*m*_, *p*_*f*_) = (0.25, 0.30)			(*p*_*m*_, *p*_*f*_) = (0.30, 0.25)		
MP	*γ*	CP (%)	ML/(ML + MR)	DP	CP (%)	ML/(ML + MR)	DP	CP (%)	ML/(ML + MR)	DP
1	0	94.37	1	0.098	94.46	1	0.099	94.74	1	0.100
	0.5	94.70	0.51	0.034	94.59	0.51	0.030	94.79	0.56	0.035
	1	95.01	0.33	0.018	94.94	0.34	0.022	94.96	0.34	0.023
	1.5	95.10	0.26	0.052	95.08	0.30	0.048	95.08	0.25	0.058
	2	95.13	0	0.061	94.84	0	0.060	94.97	0	0.072
2	0	95.17	1	0.162	94.69	1	0.161	94.78	1	0.160
	0.5	94.98	0.59	0.064	94.87	0.53	0.055	95.21	0.59	0.061
	1	94.96	0.21	0.036	95.05	0.20	0.038	95.21	0.17	0.037
	1.5	94.65	0.16	0.104	95.01	0.12	0.105	94.67	0.09	0.110
	2	94.58	0	0.130	94.98	0	0.120	94.87	0	0.140
3	0	94.79	1	0.373	95.28	1	0.347	94.99	1	0.372
	0.5	95.57	0.75	0.140	95.58	0.62	0.119	95.58	0.75	0.138
	1	95.49	0.01	0.071	95.72	0.02	0.069	95.56	0.03	0.063
	1.5	94.84	0	0.232	94.93	0	0.227	94.96	0	0.210
	2	94.87	0	0.413	94.75	0	0.385	95.13	0	0.393
4	0	94.72	1	0.500	94.59	1	0.472	94.76	1	0.488
	0.5	95.14	0.87	0.183	95.31	0.81	0.162	95.32	0.89	0.174
	1	94.99	0.05	0.067	95.13	0.03	0.073	94.85	0.04	0.070
	1.5	94.79	0	0.229	94.86	0	0.238	94.65	0	0.194
	2	94.65	0	0.457	94.39	0	0.460	94.81	0	0.402
5	0	94.77	1	0.211	95.12	1	0.204	95.08	1	0.209
	0.5	95.56	0.67	0.077	95.61	0.56	0.066	95.60	0.66	0.078
	1	95.04	0.12	0.050	95.06	0.13	0.052	95.49	0.11	0.047
	1.5	94.72	0.04	0.155	94.83	0.07	0.150	94.69	0.05	0.150
	2	94.74	0	0.220	94.66	0	0.199	94.81	0	0.231
6	0	95.08	1	0.295	95.25	1	0.275	95.05	1	0.291
	0.5	95.28	0.72	0.108	95.43	0.62	0.094	95.68	0.77	0.106
	1	95.20	0.06	0.059	95.30	0.05	0.063	95.03	0.03	0.059
	1.5	94.57	0.01	0.190	95.09	0.03	0.183	94.64	0.01	0.186
	2	94.87	0	0.294	94.69	0	0.267	94.64	0	0.297

^a^The simulations are conducted under 10,000 replicates and 5% significance level

Table 4 shows the corresponding statistical properties of the CI of *γ* with *ρ*=0, *λ*_2_=2, and (*p*_*m*_, *p*_*f*_) being (0.30, 0.30), (0.25, 0.30) and (0.30, 0.25). As expected, the CP is still controlled well, the ML/(ML + MR) is closer to 0.5 and DP is lower with larger *λ*_2_. We also investigate the effect of *ρ*=0.05, and the corresponding results are similar to those of *ρ*=0 (see Additional file 1: Tables S4 and S5). On the other hand, when (*p*_*m*_, *p*_*f*_) is set to be (0.20, 0.20), (0.15, 0.20) and (0.20, 0.15), the results are similar to those when (*p*_*m*_, *p*_*f*_) being taken as (0.30, 0.30), (0.25, 0.30) and (0.30, 0.25) (see Additional file 1: Tables S6–S9). In addition, the median of the point estimates of *γ* among *k* replicates under each simulation scenario is shown in Additional file 1: Figures S1 and S2. From Additional file 1: Figure S1, we can see that the median of $\widehat{\gamma}$ gets more far away from the true value of *γ* as the missing pattern varies from MP1 to MP4, and it is always slightly closer to *γ* under MP5 than that under MP6. The increase of the value of *λ*_2_ also improves the accuracy of the median of $\widehat{\gamma}$, while the values of *ρ*, *p*_*m*_ and *p*_*f*_ seem to have no great influence on the median of $\widehat{\gamma}$.

**Table 4:** Statistical properties of likelihood-based confidence interval of *γ* against missing pattern (MP) and *γ* with *ρ*=0, *λ*_2_=2, and (*p*_*m*_, *p*_*f*_) being (0.30, 0.30), (0.25, 0.30) and (0.30, 0.25)^a^

		(*p*_*m*_, *p*_*f*_) = (0.30, 0.30)			(*p*_*m*_, *p*_*f*_) = (0.25, 0.30)			(*p*_*m*_, *p*_*f*_) = (0.30, 0.25)		
MP	*γ*	CP (%)	ML/(ML + MR)	DP	CP (%)	ML/(ML + MR)	DP	CP (%)	ML/(ML + MR)	DP
1	0	94.67	0.88	0.025	94.73	0.90	0.030	94.80	0.93	0.029
	0.5	94.71	0.40	0.005	94.95	0.42	0.006	94.65	0.41	0.008
	1	94.86	0.41	0.004	94.97	0.40	0.004	95.09	0.41	0.006
	1.5	94.96	0.41	0.006	95.25	0.43	0.005	95.11	0.41	0.008
	2	95.14	0.02	0.022	94.99	0.01	0.024	95.08	0.01	0.024
2	0	95.17	1	0.032	94.71	0.99	0.040	94.64	1	0.038
	0.5	95.16	0.40	0.025	95.13	0.35	0.022	95.00	0.38	0.027
	1	94.60	0.36	0.019	95.24	0.38	0.019	95.01	0.33	0.020
	1.5	94.62	0.39	0.026	95.32	0.43	0.020	94.93	0.40	0.034
	2	94.71	0	0.028	94.81	0	0.026	95.13	0	0.029
3	0	94.91	1	0.168	95.10	1	0.189	95.09	1	0.172
	0.5	95.24	0.29	0.159	95.61	0.27	0.135	95.56	0.31	0.164
	1	95.58	0.02	0.097	95.51	0.02	0.101	95.25	0	0.091
	1.5	95.21	0.03	0.280	95.06	0.04	0.250	94.92	0	0.296
	2	95.16	0	0.176	95.07	0	0.142	95.10	0	0.219
4	0	94.74	1	0.387	94.68	1	0.406	94.57	1	0.393
	0.5	94.80	0.43	0.263	94.61	0.32	0.227	94.51	0.46	0.275
	1	95.19	0.01	0.127	95.37	0	0.155	95.20	0.01	0.110
	1.5	94.67	0	0.449	94.54	0	0.455	94.69	0	0.405
	2	94.65	0	0.433	94.71	0	0.399	94.61	0	0.462
5	0	94.75	1	0.050	94.94	1	0.063	94.93	1	0.054
	0.5	95.18	0.36	0.050	95.73	0.37	0.046	95.31	0.39	0.058
	1	94.98	0.25	0.037	94.85	0.26	0.042	95.21	0.22	0.037
	1.5	94.97	0.23	0.084	94.71	0.31	0.065	94.85	0.25	0.093
	2	95.24	0	0.037	94.42	0	0.035	94.63	0	0.051
6	0	95.34	1	0.083	95.10	1	0.095	95.20	1	0.101
	0.5	95.72	0.36	0.092	95.24	0.36	0.081	95.48	0.33	0.094
	1	94.93	0.09	0.059	94.91	0.12	0.062	94.83	0.10	0.063
	1.5	94.96	0.14	0.133	94.57	0.18	0.119	95.07	0.13	0.146
	2	94.81	0	0.055	94.71	0	0.054	94.95	0	0.075

^a^The simulations are conducted under 10,000 replicates and 5% significance level

**Fig. 1:**
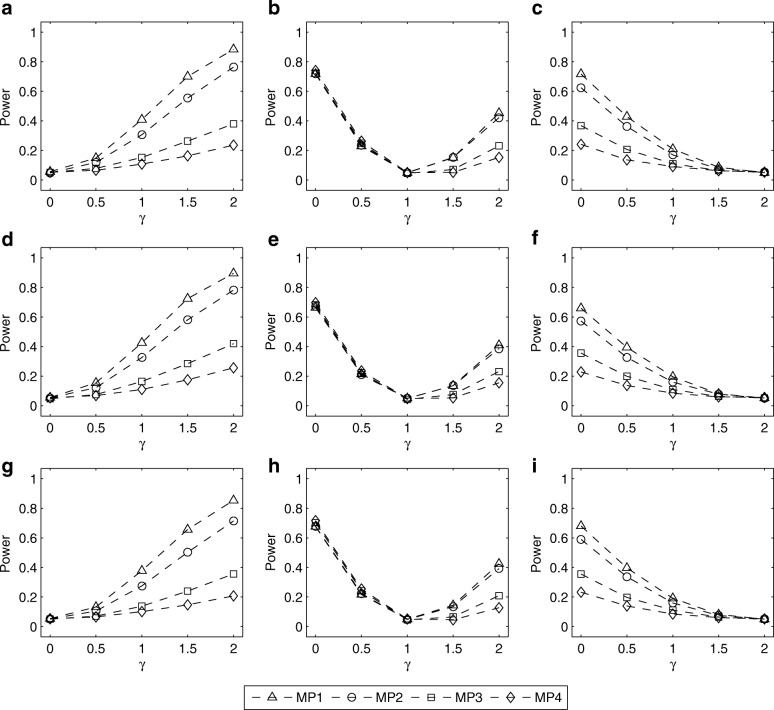
Estimated powers of LRT against *γ* under MP1–MP4 with *ρ*=0 and *λ*_2_= 1.5. The results are based on 10,000 replicates and 5% significance level. **a** (*p*_*m*_, *p*_*f*_) = (0.30, 0.30) and *γ*_0_=0; **b** (*p*_*m*_, *p*_*f*_) = (0.30, 0.30) and *γ*_0_=1; **c** (*p*_*m*_, *p*_*f*_) = (0.30, 0.30) and *γ*_0_=2; **d** (*p*_*m*_, *p*_*f*_) = (0.25, 0.30) and *γ*_0_=0; **e** (*p*_*m*_, *p*_*f*_) = (0.25, 0.30) and *γ*_0_=1; **f** (*p*_*m*_, *p*_*f*_) = (0.25, 0.30) and *γ*_0_=2; **g** (*p*_*m*_, *p*_*f*_) = (0.30, 0.25) and *γ*_0_=0; **h** (*p*_*m*_, *p*_*f*_) = (0.30, 0.25) and *γ*_0_=1; **i** (*p*_*m*_, *p*_*f*_) = (0.30, 0.25) and *γ*_0_=2. Note that *γ*_0_=0, 1 and 2 represent XCI skewing completely against mutant allele, random XCI and XCI skewing completely toward mutant allele, respectively

**Fig. 2:**
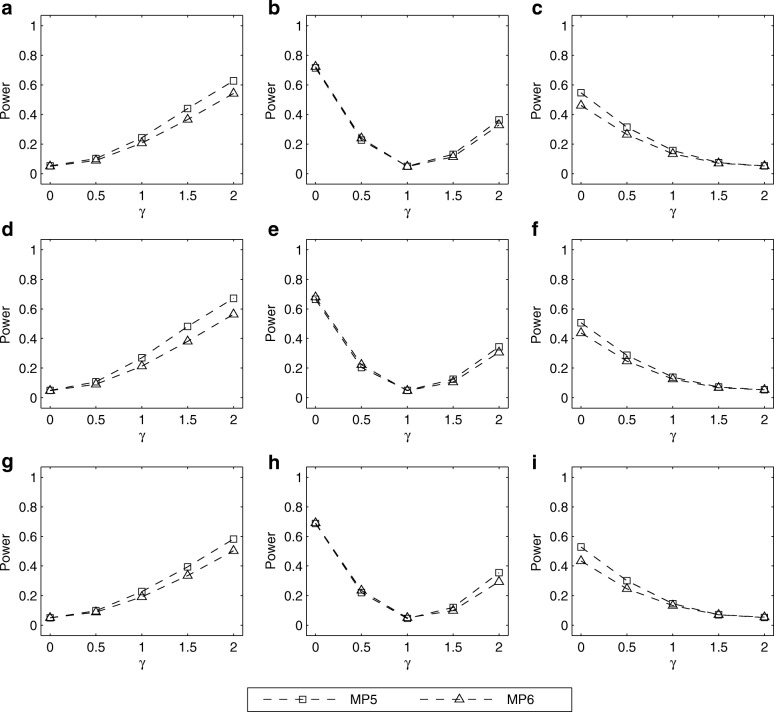
Estimated powers of LRT against *γ* under MP5 and MP6 with *ρ*=0 and *λ*_2_= 1.5. The results are based on 10,000 replicates and 5% significance level. **a** (*p*_*m*_, *p*_*f*_) = (0.30, 0.30) and *γ*_0_=0; **b** (*p*_*m*_, *p*_*f*_) = (0.30, 0.30) and *γ*_0_=1; **c** (*p*_*m*_, *p*_*f*_) = (0.30, 0.30) and *γ*_0_=2; **d** (*p*_*m*_, *p*_*f*_) = (0.25, 0.30) and *γ*_0_=0; **e** (*p*_*m*_, *p*_*f*_) = (0.25, 0.30) and *γ*_0_=1; **f** (*p*_*m*_, *p*_*f*_) = (0.25, 0.30) and *γ*_0_=2; **g** (*p*_*m*_, *p*_*f*_) = (0.30, 0.25) and *γ*_0_=0; **h** (*p*_*m*_, *p*_*f*_) = (0.30, 0.25) and *γ*_0_=1; **i** (*p*_*m*_, *p*_*f*_) = (0.30, 0.25) and *γ*_0_=2. Note that *γ*_0_=0, 1 and 2 represent XCI skewing completely against mutant allele, random XCI and XCI skewing completely toward mutant allele, respectively

**Fig. 3:**
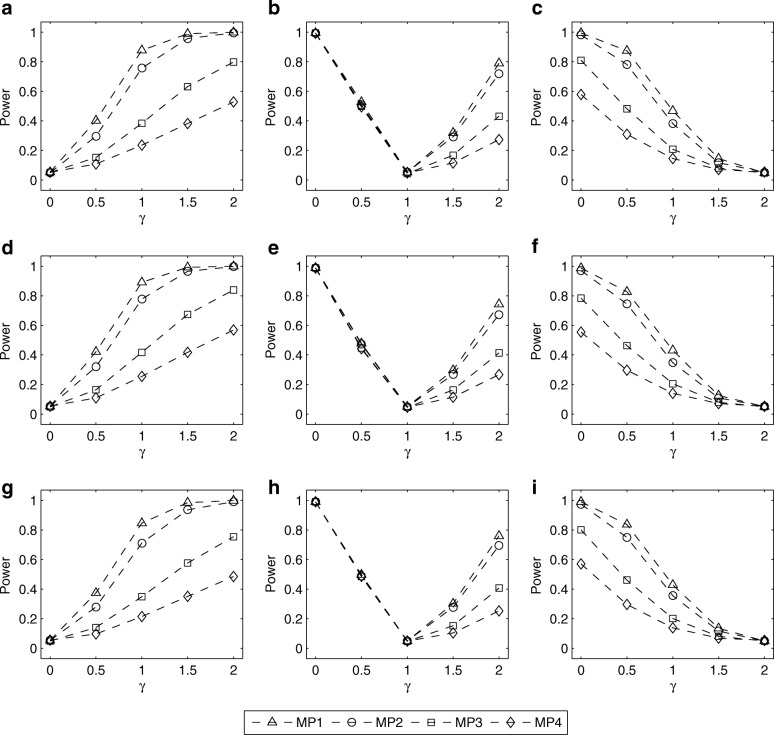
Estimated powers of LRT against *γ* under MP1–MP4 with *ρ*=0 and *λ*_2_=2. The results are based on 10,000 replicates and 5% significance level. **a** (*p*_*m*_, *p*_*f*_) = (0.30, 0.30) and *γ*_0_=0; **b** (*p*_*m*_, *p*_*f*_) = (0.30, 0.30) and *γ*_0_=1; **c** (*p*_*m*_, *p*_*f*_) = (0.30, 0.30) and *γ*_0_=2; **d** (*p*_*m*_, *p*_*f*_) = (0.25, 0.30) and *γ*_0_=0; **e** (*p*_*m*_, *p*_*f*_) = (0.25, 0.30) and *γ*_0_=1; **f** (*p*_*m*_, *p*_*f*_) = (0.25, 0.30) and *γ*_0_=2; **g** (*p*_*m*_, *p*_*f*_) = (0.30, 0.25) and *γ*_0_=0; **h** (*p*_*m*_, *p*_*f*_) = (0.30, 0.25) and *γ*_0_=1; **i** (*p*_*m*_, *p*_*f*_) = (0.30, 0.25) and *γ*_0_=2. Note that *γ*_0_=0, 1 and 2 represent XCI skewing completely against mutant allele, random XCI and XCI skewing completely toward mutant allele, respectively

**Fig. 4:**
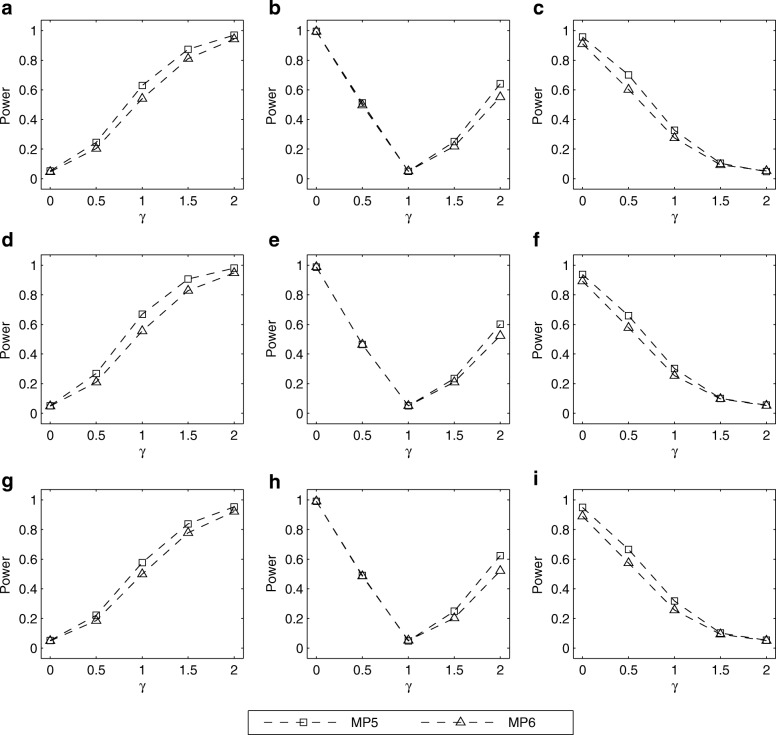
Estimated powers of LRT against *γ* under MP5 and MP6 with *ρ*=0 and *λ*_2_=2. The results are based on 10,000 replicates and 5% significance level. **a** (*p*_*m*_, *p*_*f*_) = (0.30, 0.30) and *γ*_0_=0; **b** (*p*_*m*_, *p*_*f*_) = (0.30, 0.30) and *γ*_0_=1; **c** (*p*_*m*_, *p*_*f*_) = (0.30, 0.30) and *γ*_0_=2; **d** (*p*_*m*_, *p*_*f*_) = (0.25, 0.30) and *γ*_0_=0; **e** (*p*_*m*_, *p*_*f*_) = (0.25, 0.30) and *γ*_0_=1; **f** (*p*_*m*_, *p*_*f*_) = (0.25, 0.30) and *γ*_0_=2; **g** (*p*_*m*_, *p*_*f*_) = (0.30, 0.25) and *γ*_0_=0; **h** (*p*_*m*_, *p*_*f*_) = (0.30, 0.25) and *γ*_0_=1; **i** (*p*_*m*_, *p*_*f*_) = (0.30, 0.25) and *γ*_0_=2. Note that *γ*_0_=0, 1 and 2 represent XCI skewing completely against mutant allele, random XCI and XCI skewing completely toward mutant allele, respectively

The simulated powers of the LRT for testing *H*_0_ : *γ* = *γ*_0_ with (*p*_*m*_, *p*_*f*_) = (0.30, 0.30), (0.25, 0.30) and (0.30, 0.25) are given in Figs. 1, 2, 3, 4. Fig. 1 shows the simulated powers of the LRT against *γ* under MP1–MP4 with *ρ*= 0 and *λ*_2_=1.5. From the first row to the third row of the panels in Fig. 1, (*p*_*m*_, *p*_*f*_) is set to be (0.30, 0.30), (0.25, 0.30) and (0.30, 0.25), respectively. From the first column to the third column, *γ*_0_ is fixed at 0, 1 and 2, respectively. It is found that the power increases as the value of ∣*γ* − *γ*_0_∣ gets larger. For example, in Fig. 1(a), when testing for *H*_0_: *γ* = *γ*_0_ with *γ*_0_= 0 (XCI skewing completely against mutant allele), the power under *γ*=1.5 (75% cells express mutant allele) is greater than that under *γ*= 0.5 (25% cells express mutant allele). On the other hand, when the missing pattern changes from MP1 to MP4, the loss in power is always substantial. Also, we compare the corresponding powers under MP5 and MP6 in Fig. 2. The power under MP5 is always higher than that under MP6 when *γ* ≠ *γ*_0_, which implies that the mother-daughter pairs contain more information on the skewness of XCI than the father-daughter pairs. This is not surprising because when the father’s genotype is missing in a trio, it can be inferred according to the available mother’s and daughter’s genotypes, except for the mother-daughter pair of type *MC* = 11, whereas we cannot infer the missing mother’s genotypes from any father-daughter pairs. In addition, Figs. 3 and 4 give the simulated powers of the LRT under *ρ*=0 and *λ*_2_=2 for MP1–MP4 and MP5–MP6, respectively. It is shown that the increase of *λ*_2_ leads to the growth in power (Fig. 3 vs. Fig. 1, Fig. 4 vs. Fig. 2). We also simulate the powers under *ρ*=0.05 and (*p*_*m*_, *p*_*f*_) = (0.30, 0.30), (0.25, 0.30) and (0.30, 0.25), which are similar to those under *ρ*=0 (see Additional file 1: Figures S3–S6). Finally, the simulated powers under (*p*_*m*_, *p*_*f*_) = (0.20, 0.20), (0.15, 0.20) and (0.20, 0.15) are given in Additional file 1: Figures S7–S14, which are always lower than those under (*p*_*m*_, *p*_*f*_) = (0.30, 0.30), (0.25, 0.30) and (0.30, 0.25), respectively.

### Application to RA data

Rheumatoid arthritis (RA) is an autoimmune disease, which has been reported to be associated with the skewness of XCI [33]. To investigate the XCI skewing patterns at the X-linked loci associated with RA, we apply our proposed method to the data from North American Rheumatoid Arthritis Consortium [29], which is made available from Genetic Analysis Workshop 15 [34]. The dataset includes 757 pedigrees and 293 single nucleotide polymorphism (SNP) markers on X chromosome. In this application, one nuclear family with a typed affected daughter is selected randomly from each pedigree. As such, a total of 703 nuclear families are included, which contains 64 case-parents trios, 179 mother-daughter pairs, 37 father-daughter pairs and 423 single daughters.

Since our proposed method is applicable in the presence of association, we ultimately measure the degree of XCI skewing at five SNPs which have been found to be associated with RA by the XMCPDT method at the significance level of 1% [35]. Notice that the XMCPDT method is conducted based on 246 pedigrees from the RA dataset. We identify the at-risk allele by the value of ${\widehat{\lambda}}_2$, and denote the estimates of the frequencies of the at-risk allele in males and females obtained from the ECM algorithm by ${\widehat{p}}_m$ and ${\widehat{p}}_f$, respectively. Table 5 lists the *p*-value of XMCPDT, the values of (${\widehat{p}}_m$, ${\widehat{p}}_f$), ${\widehat{\lambda}}_2$ and $\widehat{\gamma}$, and 95% CI of *γ* based on the proposed likelihood method for each of five SNPs. From Table 5, we find that there are three SNPs (rs916685, rs1043034 and rs2005463) with the 95% CIs containing the value of *γ*=1, which indicates the random XCI. On the other hand, the XCI skewing at rs2238907 is found with $\widehat{\gamma}=$0.35 and the 95% CI being [0, 0.79), which suggests that the skewness of XCI is against the at-risk allele with 17.5% (0.35/2) cells in heterozygous females having the at-risk allele active, while the other 82.5% cells keeping the normal allele active. However, the 95% CI of *γ* at rs1264064 is [0, 2], providing no information on the XCI skewing pattern. In addition, we evaluate $\widehat{\gamma}$’s and the 95% CIs of *γ* at the rest 288 SNPs in the RA dataset, and find that there are 21 SNPs with nonrandom XCI pattern. But note that, if we assume that all of 293 SNPs except for rs2238907 are under *H*_0_: *γ* = 1, then the corresponding false positive rate would be 0.0719 (21/292), which is still below the upper bound $0.05+1.96\kern0.5em \times \kern0.5em \sqrt{0.05\kern0.5em \times \kern0.5em \left(1-0.05\right)/292}=\mathrm{0.0750.}$ Besides, association between these 21 SNPs and RA has not been found by XMCPDT at the 1% significance level, so we should draw conclusions with this caution.

**Table 5:** Application of proposed method to RA dataset with *p*-values of XMCPDT less than 1% significance level

SNP name	*p*-value^a^	(${\widehat{p}}_m$, ${\widehat{p}}_f$)	${\widehat{\lambda}}_2$	$\widehat{\gamma}$	95% CI of *γ*
rs2238907	0.004	(0.20, 0.24)	2.18	0.35	[0, 0.79)
rs916685	0.003	(0.17, 0.20)	2.55	0.53	[0, 1.33)
rs1264064	0.001	(0.42, 0.45)	1.96	0.71	[0, 2]
rs1043034	0.007	(0.19, 0.24)	3.73	0.81	(0.51, 1.69)
rs2005463	0.007	(0.18, 0.23)	4.59	0.61	(0.40, 1.06)

^a^*P*-value of XMCPDT for testing association between SNP and RA [35]

## Discussion

In this article, we propose a statistical measure *γ* of the degree of the XCI skewing for family trio data, which can be represented as a ratio of two GRRs in females in the presence of association between the disease and genotypes. Further, we obtain the point estimate of *γ*, which is constructed by the MLEs of two GRRs in females. When there are missing parental genotypes in some family trios, the ECM algorithm is used to estimate the two GRRs. The CI of *γ* is derived from the likelihood method by inverting the LRT. We conduct the simulation study under various simulation settings, including six missing patterns of families, six groups of allele frequencies, two different values of inbreeding coefficient in females, two different values of *λ*_2_ and five different values of *γ*. The simulation results show that the proposed likelihood-based CI of *γ* has an accurate CP under the situations considered, while ML/(ML + MR) and DP of the CI of *γ* and the median of estimates of *γ* are influenced by the values of *γ*, *λ*_2_ and the missing pattern. Similarly, the simulated power of LRT is affected by the values of ∣*γ* − *γ*_0_∣, *λ*_2_, (*p*_*m*_, *p*_*f*_) and the missing pattern. Finally, we apply our proposed method to the RA data from USA and find out a locus, rs2238907, which may undergo the XCI skewing against the at-risk allele.

Many X-linked diseases are always associated with XCI skewing in females. Our proposed statistical measure *γ* provides information on the potential loci subject to XCI skewing, thus it is helpful to uncover the pathogenesis of X-linked diseases. However, most of the statistical studies on X chromosome today focus mainly on the association tests [17, 24, 25, 36–38], so there are no other statistical methods available to measure the skewness of XCI. On the other hand, although the XCI skewing pattern can also be detected by differential methylation between the active and inactive X chromosomes or mRNA transcription in cells, our proposed statistical method takes use of population data to measure the skewness of XCI. Thus, it can reflect the average level of the XCI skewing in female population.

There are some issues in our proposed method. First of all, the original CI is truncated by [0, 2] to enhance the interpretability of the CI. However, when the whole original CI lies outside [0, 2], the CI ultimately obtained after truncation would be empty. Although it is hard to interpret this kind of CI containing no values, the simulation results show that when *γ* takes values on the boundary (i.e., 0 and 2), these empty CIs seldom occur. For example, when *γ* = 0, two tail errors (ML and MR) are extremely imbalance with ML/(ML + MR) being 1 or close to 1. This means that there are no or very few original CIs whose upper limit is below 0. Likewise, ML/(ML + MR) is 0 or close to 0 when *γ* = 2, which implies that there are no or very few original CIs whose lower limit is beyond 2. On the other hand, the proposed likelihood method has its own drawback in deriving the CI of a ratio like any other ratio estimation methods. We find that the likelihood-based CI of *γ* may consist of two disjoint intervals, such as [0, 0.03) ∪ (0.59, 2], and it is also difficult for us to interpret. For example, if $\widehat{\gamma}=1.92$ and the CI of *γ* is [0, 0.03) ∪ (0.59, 2], then the corresponding LRT would reject the null hypothesis of *γ*_0_ = 0.5, and accept that of *γ*_0_ = 0.01. It is hard to explain that the LRT rejects a *γ*_0_ being close to $\widehat{\gamma}$, while accepts one being far away from $\widehat{\gamma}$. Although this kind of CI is undesirable, it is also inevitable and can be regarded as a hint of *λ*_2_ being close to 1 [39]. In addition, it should be noted that the ECM algorithm is not applicable when all the family trios are “single daughters”, since the MLE of *θ* may not be uniquely specified under this situation (the details see Additional file 1: Appendix C). However, if the other family trios were collected, then the single daughters can make contribution to the MLE of *θ* in the ECM algorithm together with these trio data (the details see Additional file 1: Appendix D). Finally, we assume that the genotypes’ frequencies in males and females (*p*_*m*_, *g*_0_ and *g*_1_) are unknown and estimate them together with *λ*_1_ and *λ*_2_ in our simulation study and real data application. Alternatively, if we can obtain information on the allele frequencies from the online databases, such as the Allele Frequency Net Database [40] and the UCSC Genome Browser Database [41], then it is unnecessary to re-estimate *p*_*m*_, *g*_0_ and *g*_1_, which will reduce the number of parameters so that the ECM algorithm runs faster.

Note that the ECM algorithm can converge to a local maximum of the log-likelihood function instead of a global maximum [23]. To investigate this, we randomly choose 1000 initial values of *θ* (*θ*_0_) from the parameter space and regard the MLE of *θ* (*θ*_0_) with the maximum log-likelihood among 1000 $\ln L\left(\widehat{\theta}\right)$'s ($\ln L\left({\overset{\sim }{\theta}}_0\right)$'s) as the global MLE of *θ* (*θ*_0_). We conduct a simulation study under the simulation settings with *ρ*=0, *λ*_2_= 1.5 and (*p*_*m*_, *p*_*f*_) = (0.30, 0.30), and the details see Additional file 1: Appendix E. The simulation results (see Additional file 1: Tables S10 and S11) show that the values of $\widehat{\theta}$ and $\ln L\left(\widehat{\theta}\right)$ (${\overset{\sim }{\theta}}_0$ and $\ln L\left({\overset{\sim }{\theta}}_0\right)$) based on one initial value estimated by the method described in Additional file 1: Appendix B are very close to those based on 1000 initial values under all the simulated situations when *N*_2_ (the number of complete family trios) is not too small, such as MP1 and MP2, which may indicate that the ECM algorithm based on the estimated initial value converges towards the global maximum. As for MP3-MP6, except that ${\overset{\sim }{\theta}}_0$ with (*γ*_0_, *γ*) = (1, 2) under MP5 and MP6, ${\overset{\sim }{\theta}}_0$ with (*γ*_0_, *γ*) = (1, 1) and (1, 2) under MP3, and ${\overset{\sim }{\theta}}_0$ with (*γ*_0_, *γ*) = (1, 1), (1, 1.5) and (1, 2) under MP4 may converge to a local maximum, all the other $\widehat{\theta}$ and ${\tilde{\theta}}_0$ results converge to the global maximum. Further, for these seven cases, we try and randomly select ten groups of initial values of *θ*_0_ from the parameter space and regard ${\tilde{\theta}}_0$ with the maximum log-likelihood among ten $\ln L\left({\tilde{\theta}}_0\right)$'s as the final MLE of *θ*_0_. We find that ${\tilde{\theta}}_0$’s based on ten and 1000 initial values are very close to each other under all the seven simulated situations (see Additional file 1: Table S11). So, if *N*_2_ is zero or too small, we recommend using multiple initial values (such as ten) for obtaining the global MLE of *θ*_0_. On the other hand, family trios with both parents are always fortunately collected in the family-based studies in practice.

In future studies, we will extend our proposed method to incorporate covariates by using nuclear families with affected and unaffected offspring. Furthermore, to facilitate the interpretability of the CI of *γ*, we will utilize the prior information, such as the order of the GRRs in females and the information of the presence of association.

## Conclusions

The proposed statistical measure for the skewness of XCI is applicable for complete family trio data or family trio data with some paternal genotypes missing. The likelihood-based CI has an accurate CP under the situations considered. Therefore, our proposed statistical measure is generally recommended in practice for discovering the potential loci which undergo the XCI skewing.

## Additional file

Additional file 1:**Appendix A.** Derivation of *P*(*FMC*| *D*) in Table 1. **Appendix B.** Choice of initial value of *θ* (*θ*_0_) and MLE of *θ* (*θ*_0_) using family trios with missing parental genotypes. **Appendix C.** Inapplicability of ECM algorithm when using only single daughters. **Appendix D.** Contribution of single daughters to estimate of *θ* in ECM algorithm. **Appendix E.** Effect of different initial values of *θ* (*θ*_0_) on ECM algorithm. **Tables S1**–**S3.** The conditional probabilities and conditional expectations for seven types of possible mother-daughter pairs, four types of possible father-daughter pairs and three types of possible single daughters, respectively**. Tables S4**–**S5.** Statistical properties of likelihood-based confidence interval of *γ* against missing pattern (MP) and *γ* with *ρ*=0.05, (*p*_*m*_, *p*_*f*_) = (0.30, 0.30), (0.25, 0.30) and (0.30, 0.25), and *λ*_2_=1.5 and 2, respectively. **Tables S6**–**S9.** Statistical properties of likelihood-based confidence interval of *γ* against missing pattern (MP) and *γ* with (*p*_*m*_, *p*_*f*_)= (0.20, 0.20), (0.15, 0.20) and (0.20, 0.15), *ρ*=0 and 0.05, and *λ*_2_=1.5 and 2, respectively. **Table S10.** Averages of absolute differences of each element of $\widehat{\theta}$ and $\ln L\left(\widehat{\theta}\right)$ between ECM_1_ and ECM_1000_ with *ρ*=0, *λ*_2_= 1.5 and (*p*_*m*_, *p*_*f*_) = (0.30, 0.30) under MP1-MP6. **Table S11.** Averages of absolute differences of each element of ${\tilde{\theta}}_0$ and $\ln L\left({\tilde{\theta}}_0\right)$ between ECM_1_/ECM_10_ and ECM_1000_ with *ρ*=0, *λ*_2_= 1.5 and (*p*_*m*_, *p*_*f*_) = (0.30, 0.30) under MP1-MP6. **Figures S1**–**S2.** Medians of point estimates of *γ* against MP for different *p*_*m*_, *p*_*f*_ and *λ*_2_ values with *ρ*=0 and 0.05, respectively. **Figures S3**–**S6.** Estimated powers of LRT against *γ* with *ρ*=0.05 and (*p*_*m*_, *p*_*f*_) being (0.30, 0.30), (0.25, 0.30) and (0.30, 0.25) under MP1–MP4 and MP5–MP6, and *λ*_2_=1.5 and 2, respectively. **Figures S7**–**S14.** Estimated powers of LRT against *γ* with (*p*_*m*_, *p*_*f*_) being (0.20, 0.20), (0.15, 0.20) and (0.20, 0.15) under MP1–MP4 and MP5–MP6, *ρ*=0 and 0.05, and *λ*_2_=1.5 and 2, respectively. (PDF 2328 kb)

## Abbreviations

CI: Confidence interval
CP: Coverage probability
DP: Proportion of the discontinuous CIs
ECM: Expectation-conditional- maximization
GRR: Genotypic relative risk
LRT: Likelihood ratio test
ML: Left tail error
MLE: Maximum likelihood estimate
MP: Missing patterns
MR: Right tail error
RA: Rheumatoid arthritis
SNP: Single nucleotide polymorphism
XCI: X chromosome inactivation
XMCPDT: Monte Carlo pedigree disequilibrium test on X chromosome
