# Supplementary material for: A statistical measure for the skewness of X chromosome inactivation based on family trios
Source: BMC Genet. 2018 Dec 5;19:109. doi: 10.1186/s12863-018-0694-8 (PMC6282303; doi:10.1186/s12863-018-0694-8)
Supplement: Supplementary file 1 — Appendix A. Derivation of P(FMC| D) in Table 1. Appendix B. Choice of initial value of θ (θ0) and MLE of θ (θ0) using family trios with missing parental genotypes. Appendix C. Inapplicability of ECM algorithm when using only single daughters. Appendix D. Contribution of single daughters to estimate of θ in ECM algorithm. Appendix E. Effect of different initial values of θ (θ0) on ECM algorithm. Tables S1–S3. The conditional probabilities and conditional expectations for seven types of possible mother-daughter pairs, four types of possible father-daughter pairs and three types of possible single daughters, respectively. Tables S4–S5. Statistical properties of likelihood-based confidence interval of γ against missing pattern (MP) and γ with ρ=0.05, (pm, pf) = (0.30, 0.30), (0.25, 0.30) and (0.30, 0.25), and λ2=1.5 and 2, respectively. Tables S6–S9. Statistical properties of likelihood-based confidence interval of γ against missing pattern (MP) and γ with (pm, pf)= (0.20, 0.20), (0.15, 0.20) and (0.20, 0.15), ρ=0 and 0.05, and λ2=1.5 and 2, respectively. Table S10. Averages of absolute differences of each element of $\widehat{\theta}$θ^ and $\ln L\left(\widehat{\theta}\right)$lnLθ^ between ECM1 and ECM1000 with ρ=0, λ2= 1.5 and (pm, pf) = (0.30, 0.30) under MP1-MP6. Table S11. Averages of absolute differences of each element of [file 12863_2018_694_MOESM1_ESM.pdf]

Additional file 1 for

"A statistical measure for the skewness of X chromosome inactivation based on family trios"

Si-Qi Xu[1], Yu Zhang[1], Peng Wang[1], Wei Liu[1], Xian-Bo Wu[2*] and Ji-Yuan Zhou[1*]

[1]State Key Laboratory of Organ Failure Research, Ministry of Education, and Guangdong Provincial Key Laboratory of Tropical Disease Research, Department of Biostatistics, School of Public Health, Southern Medical University, Guangzhou, China [2]Guangdong Provincial Key Laboratory of Tropical Disease Research, Department of Epidemiology, School of Public Health, Southern Medical University, Guangzhou, China

Appendix A: Derivation of $\boldsymbol{P(FMC|D)}$ in Table 1

Here we take $FMC = 122$ as an example to show the deviation of $P(FMC|D)$ and others are similar.

$$P(FMC = 122|D)$$

$$= \frac{P(F = 1, M = 2)\, P(C = 2|F = 1, M = 2)\, P(D|C = 2)}{\sum_{F'M'C'\in\Omega} P(F'M')\, P(C'|F'M')\, P(D|C')}$$

$$= \frac{p_m g_2 f_2}{q_m g_0 f_0 + 0.5 q_m g_1 f_0 + 0.5 q_m g_1 f_1 + q_m g_2 f_1 + p_m g_0 f_1 + 0.5 p_m g_1 f_1 + 0.5 p_m g_1 f_2 + p_m g_2 f_2}$$

$$= \frac{p_m g_2 \lambda_2}{q_m g_0 + 0.5 q_m g_1 + 0.5 q_m g_1 \lambda_1 + q_m g_2 \lambda_1 + p_m g_0 \lambda_1 + 0.5 p_m g_1 \lambda_1 + 0.5 p_m g_1 \lambda_2 + p_m g_2 \lambda_2}$$

$$= \frac{p_m g_2 \lambda_2}{R},$$

where $R = q_m g_0 + 0.5 q_m g_1 (1 + \lambda_1) + q_m g_2 \lambda_1 + p_m g_0 \lambda_1 + 0.5 p_m g_1 (\lambda_1 + \lambda_2) + p_m g_2 \lambda_2$.

Appendix B: Choice of initial value of $\boldsymbol{\theta}$ ($\boldsymbol{\theta_0}$) and MLE of $\boldsymbol{\theta}$ ($\boldsymbol{\theta_0}$) using family trios with missing parental genotypes

Choice of initial value of $\boldsymbol{\theta}$

The initial values of p_m, g_0 and g_1 are estimated as follows:

$$\hat{p}_m^{(0)} = \frac{\#(F = 1)}{\#(F \in \{0,1\})},$$

$$\hat{g}_0^{(0)} = \frac{\#(M = 0)}{\#(M \in \{0,1,2\})}$$

and

$$\hat{g}_1^{(0)} = \frac{\#(M = 1)}{\#(M \in \{0,1,2\})},$$

where $\#$ denotes the counting measure. Notice that $\hat{p}_m^{(0)}$ is the proportion of fathers with genotype A among all the fathers, while $\hat{g}_0^{(0)}$ and $\hat{g}_1^{(0)}$ are the proportions of mothers with genotypes aa and Aa among all the mothers, respectively. Then, $\hat{q}_m^{(0)} = 1 - \hat{p}_m^{(0)}$ and $\hat{g}_2^{(0)} = 1 - \hat{g}_0^{(0)} - \hat{g}_1^{(0)}$.

To obtain the initial values of λ_1 and λ_2, we construct a likelihood function based on the conditional probabilities $P(C|FM, D)$. Here, we only use family trios with both parents. Note that

$$P(C|FM, D) = \frac{P(FMC, D)}{P(FM, D)} = \frac{P(FM)P(C|FM)P(D|C)}{\sum_{C\in\{0,1,2\}} P(FM)P(C'|FM)P(D|C')}$$

$$= \frac{P(C|FM)P(D|C)}{\sum_{C\in\{0,1,2\}} P(C'|FM)P(D|C')},$$

where $P(C|FM)$ for trio type FMC is given in Table 1. $P(D|C)$ and $P(D|C')$ take possible values of f_0, f_1 and f_2, which causes that $P(C|FM, D)$ is a function of λ_1 and λ_2. Then, the log-likelihood function $\ln L_S(\lambda_1, \lambda_2)$ conditional on paternal

genotypes and the event that the daughter is affected is

$$\ln L_S(\lambda_1, \lambda_2) = \sum_{FMC\in\Omega} n_{FMC} \ln P(C|FM, D)$$

$$= n_{010} \ln\left(\frac{1}{1+\lambda_1}\right) + n_{011} \ln\left(\frac{\lambda_1}{1+\lambda_1}\right) + n_{111} \ln\left(\frac{\lambda_1}{\lambda_1+\lambda_2}\right)$$

$$+n_{112} \ln\left(\frac{\lambda_2}{\lambda_1+\lambda_2}\right)$$

$$= (n_{011} + n_{111}) \ln \lambda_1 + n_{112} \ln \lambda_2 - (n_{010} + n_{011}) \ln(1+\lambda_1)$$

$$-(n_{111} + n_{112}) \ln(\lambda_1 + \lambda_2),$$

where the second equality holds by dividing the numerator and denominator of $P(C|FM, D)$ by f_0. By setting the derivatives of the above log-likelihood function, $\partial \ln L_S(\lambda_1, \lambda_2)/\partial\lambda_1$ and $\partial \ln L_S(\lambda_1, \lambda_2)/\partial\lambda_2$, to zero, we get the MLEs of λ_1 and λ_2, and regard them as the initial values of λ_1 and λ_2. So, $\hat{\lambda}_1^{(0)} = n_{011}/n_{010}$ and $\hat{\lambda}_2^{(0)} = (n_{011}n_{112})/(n_{010}n_{111})$. Note that the MLEs of λ_1 and λ_2 based on $\ln L_S(\lambda_1, \lambda_2)$ only use four types of case-parents trios (i.e., 010, 011, 111 and 112). However, to obtain the MLEs of λ_1 and λ_2 further using the case-parents trios of other four types (i.e., 000, 021, 101 and 122) and family trios with missing paternal genotypes, we need to construct a likelihood function based on the probabilities $P(FMC|D)$ in Table 1.

When there are no complete family trios available ($N_2 = 0$), we estimate the initial values of λ_1 and λ_2 by replacing unknown n_{010}, n_{011}, n_{111} and n_{112} values in $\hat{\lambda}_1^{(0)} = n_{011}/n_{010}$ and $\hat{\lambda}_2^{(0)} = (n_{011}n_{112})/(n_{010}n_{111})$ by their respective conditional expectations (see Additional file 1: Tables S1-S3). For example, n_{011} is replaced by

$$E\left(z_{1m,011}\middle|n_{1m,11}\right) + E\left(z_{1f,011}\middle|n_{1f,01}\right) + E\left(z_{0,011}\middle|n_{0,1}\right)$$

$$= n_{1m,11}\hat{q}_m^{(0)} + n_{1f,01} \cdot \frac{0.5\hat{g}_1^{(0)}}{0.5\hat{g}_1^{(0)} + \hat{g}_2^{(0)}} + n_{0,1} \cdot \frac{0.5\hat{q}_m^{(0)}\hat{g}_1^{(0)}}{\hat{p}_m^{(0)}\hat{g}_0^{(0)} + 0.5\hat{g}_1^{(0)} + \hat{q}_m^{(0)}\hat{g}_2^{(0)}},$$

where $\hat{p}_m^{(0)}$, $\hat{q}_m^{(0)} = 1 - \hat{p}_m^{(0)}$, $\hat{g}_0^{(0)}$, $\hat{g}_1^{(0)}$ and $\hat{g}_2^{(0)} = 1 - \hat{g}_0^{(0)} - \hat{g}_1^{(0)}$ are the initial values of p_m, q_m, g_0, g_1 and g_2 mentioned above, respectively.

Choice of initial value of $\boldsymbol{\theta_0}$

Under the null hypothesis H_0: $\gamma = \gamma_0$, the initial values of p_m, g_0 and g_1 are estimated in a way similar to that under the alternative hypothesis. On the other hand, since $\lambda_1 = \gamma_0(\lambda_2 - 1)/2 + 1$ under H_0, $P(C|FM, D)$ is only a function of λ_2. Here, we only use family trios with both parents. Then, the log-likelihood function conditional on paternal genotypes and the event that the daughter is affected turns to be

$$\ln L_{S0}(\lambda_2) = \sum_{FMC \in \Omega} n_{FMC} \ln P(C|FM, D)$$

$$= (n_{011} + n_{111}) \ln\left[\frac{\gamma_0(\lambda_2 - 1)}{2} + 1\right] + n_{112} \ln \lambda_2$$

$$-(n_{010} + n_{011}) \ln\left[\frac{\gamma_0(\lambda_2 - 1)}{2} + 2\right]$$

$$-(n_{111} + n_{112}) \ln\left[\frac{\gamma_0(\lambda_2 - 1)}{2} + \lambda_2 + 1\right].$$

The derivative of $\ln L_{S0}(\lambda_2)$ with respect to λ_2 is

$$\frac{\partial \ln L_{S0}(\lambda_2)}{\partial \lambda_2} = \frac{n_{112}}{\lambda_2} + \frac{\gamma_0}{2}\left[\frac{n_{011} + n_{111}}{\frac{\gamma_0(\lambda_2 - 1)}{2} + 1} - \frac{n_{010} + n_{011}}{\frac{\gamma_0(\lambda_2 - 1)}{2} + 2}\right]$$

$$-\frac{(n_{111} + n_{112})\left(\frac{\gamma_0}{2} + 1\right)}{\frac{\gamma_0(\lambda_2 - 1)}{2} + \lambda_2 + 1}.$$

The MLE of λ_2 can be obtained by solving $\frac{\partial \ln L_{S0}(\lambda_2)}{\partial \lambda_2} = 0$, i.e., the equation

$A\lambda_2{}^3 + B\lambda_2{}^2 + C\lambda_2 + D = 0$, where

$A = -n_{010}(\gamma_0{}^3 + 2\gamma_0{}^2)$,

$B = (2n_{010} - n_{112})\gamma_0{}^3 + 2(n_{011} - n_{010} + n_{111} + n_{112})\gamma_0{}^2$

$+4(n_{011} - n_{010} - n_{111})\gamma_0$,

$C = (2n_{112} - n_{010})\gamma_0{}^3 + 2(2n_{010} - n_{011} - n_{111} - 5n_{112})\gamma_0{}^2$

$+4(n_{011} - n_{010} + 3n_{111} + 3n_{112})\gamma_0 - 16n_{111}$,

$D = -n_{112}(\gamma_0{}^3 + 8\gamma_0{}^2 - 20\gamma_0 + 16)$.

Note that when there are more than one solutions to the above equation, we choose the one which maximizes $\ln L_{S0}(\lambda_2)$ as the MLE of λ_2. Once the MLE of λ_2 is obtained, we use it as the initial value of λ_2 under the null hypothesis. When there are no complete family trios available ($N_2 = 0$), we obtain the initial value of λ_2 in a way similar to that under the alternative hypothesis by replacing the numbers of four types of case-parents trios (i.e., n_{010}, n_{011}, n_{111} and n_{112}) in the equation $A\lambda_2{}^3 + B\lambda_2{}^2 + C\lambda_2 + D = 0$ by their respective conditional expectations.

ECM algorithm under the alternative hypothesis

Let $N_{FMC} = n_{FMC} + z_{1m,FMC} + z_{1f,FMC} + z_{0,FMC}$. In the E-step at iteration (k+1), from Equation (5) and Table 1, the Q function is given by

$$Q(\theta|\hat{\theta}^{(k)}) = A_1^{(k)}\ln(1-p_m) + A_2^{(k)}\ln p_m + A_3^{(k)}\ln g_0 + A_4^{(k)}\ln g_1 + A_5^{(k)}\ln(1-g_0-g_1) + A_6^{(k)}\ln\lambda_1 + A_7^{(k)}\ln\lambda_2 - N\ln R - E_{\hat{\theta}^{(k)}}(N_{010} + N_{011} + N_{111} + N_{112})\ln 2, \quad \text{(A1)}$$

where

$A_1^{(k)} = E_{\hat{\theta}^{(k)}}(N_{000} + N_{010} + N_{011} + N_{021})$,

$A_2^{(k)} = E_{\hat{\theta}^{(k)}}(N_{101} + N_{111} + N_{112} + N_{122}),$

$A_3^{(k)} = E_{\hat{\theta}^{(k)}}(N_{000} + N_{101}),$

$A_4^{(k)} = E_{\hat{\theta}^{(k)}}(N_{010} + N_{011} + N_{111} + N_{112}),$

$A_5^{(k)} = E_{\hat{\theta}^{(k)}}(N_{021} + N_{122}),$

$A_6^{(k)} = E_{\hat{\theta}^{(k)}}(N_{011} + N_{021} + N_{101} + N_{111}),$

$A_7^{(k)} = E_{\hat{\theta}^{(k)}}(N_{112} + N_{122}).$

In the CM-steps, the first order partial derivative of the Q function (A1) with respect to p_m is

$$\frac{\partial Q(\theta|\hat{\theta}^{(k)})}{\partial p_m} = -\frac{A_1^{(k)}}{1-p_m} + \frac{A_2^{(k)}}{p_m} - \frac{NB_1^{(k)}}{p_m B_1^{(k)} + B_2^{(k)}},$$

where

$B_1^{(k)} = \hat{g}_0^{(k)}\left(\hat{\lambda}_1^{(k)} - 1\right) + 0.5\hat{g}_1^{(k)}\left(\hat{\lambda}_2^{(k)} - 1\right) + \hat{g}_2^{(k)}\left(\hat{\lambda}_2^{(k)} - \hat{\lambda}_1^{(k)}\right),$

$B_2^{(k)} = \hat{g}_0^{(k)} + 0.5\hat{g}_1^{(k)}\left(1 + \hat{\lambda}_1^{(k)}\right) + \hat{g}_2^{(k)}\hat{\lambda}_1^{(k)}.$

By solving the equation $\partial Q(\theta|\hat{\theta}^{(k)})/\partial p_m = 0,$

$$\hat{p}_m^{(k+1)} = \frac{A_2^{(k)}B_2^{(k)}}{A_1^{(k)}B_1^{(k)} + NB_2^{(k)}}$$

and $\hat{q}_m^{(k+1)} = 1 - \hat{p}_m^{(k+1)}.$

The first order partial derivative of the Q function (A1) with respect to g_0 is

$$\frac{\partial Q(\theta|\hat{\theta}^{(k)})}{\partial g_0} = \frac{A_3^{(k)}}{g_0} - \frac{A_5^{(k)}}{C_1^{(k)} - g_0} - \frac{NC_2^{(k)}}{g_0 C_2^{(k)} + C_3^{(k)}},$$

where

$C_1^{(k)} = 1 - \hat{g}_1^{(k)},$

$C_2^{(k)} = \hat{q}_m^{(k+1)}\left(1 - \hat{\lambda}_1^{(k)}\right) + \hat{p}_m^{(k+1)}\left(\hat{\lambda}_1^{(k)} - \hat{\lambda}_2^{(k)}\right),$

$C_3^{(k)} = 0.5\hat{g}_1^{(k)}C_2^{(k)} + \hat{q}_m^{(k+1)}\hat{\lambda}_1^{(k)} + \hat{p}_m^{(k+1)}\hat{\lambda}_2^{(k)}.$

$\hat{g}_0{}^{(k+1)}$ can be obtained by solving $\partial Q(\theta|\hat{\theta}^{(k)})/\partial g_0 = 0$, i.e.,

$$(A_3^{(k)} + A_5^{(k)} - N)C_2^{(k)}\left[\hat{g}_0^{(k+1)}\right]^2 + [(A_3^{(k)} + A_5^{(k)})C_3^{(k)}$$
$$+(N - A_3^{(k)})C_1^{(k)}C_2^{(k)}]\hat{g}_0^{(k+1)} - A_3^{(k)}C_1^{(k)}C_3^{(k)} = 0.$$

Note that when there are more than one solutions to the above equation, we choose the one which is closer to $\hat{g}_0^{(k)}$.

The first order partial derivative of the Q function (A1) with respect to g_1 is

$$\frac{\partial Q(\theta|\hat{\theta}^{(k)})}{\partial g_1} = \frac{A_4^{(k)}}{g_1} - \frac{A_5^{(k)}}{D_1^{(k)} - g_1} - \frac{ND_2^{(k)}}{g_1 D_2^{(k)} + D_3^{(k)}},$$

where

$D_1^{(k)} = 1 - \hat{g}_0^{(k+1)}$,

$D_2^{(k)} = 0.5\hat{q}_m^{(k+1)}\left(1 - \hat{\lambda}_1^{(k)}\right) + 0.5\hat{p}_m^{(k+1)}\left(\hat{\lambda}_1^{(k)} - \hat{\lambda}_2^{(k)}\right)$,

$D_3^{(k)} = \hat{g}_0^{(k+1)}D_2^{(k)} + \hat{q}_m^{(k+1)}\hat{\lambda}_1^{(k)} + \hat{p}_m^{(k+1)}\hat{\lambda}_2^{(k)}$.

$\hat{g}_1^{(k+1)}$ can be derived by solving $\partial Q(\theta|\hat{\theta}^{(k)})/\partial g_1 = 0$, i.e.,

$$(A_4^{(k)} + A_5^{(k)} - N)D_2^{(k)}\left[\hat{g}_1^{(k+1)}\right]^2 + [(A_4^{(k)} + A_5^{(k)})D_3^{(k)}$$
$$+(N - A_4^{(k)})D_1^{(k)}D_2^{(k)}]\hat{g}_1^{(k+1)} - A_4^{(k)}D_1^{(k)}D_3^{(k)} = 0.$$

Note that when there are more than one solutions to the above equation, we choose the one which is closer to $\hat{g}_1^{(k)}$. And $\hat{g}_2^{(k+1)} = 1 - \hat{g}_0^{(k+1)} - \hat{g}_1^{(k+1)}$.

The first order partial derivative of the Q function (A1) with respect to λ_1 is

$$\frac{\partial Q(\theta|\hat{\theta}^{(k)})}{\partial \lambda_1} = \frac{A_6^{(k)}}{\lambda_1} - \frac{NE_1^{(k)}}{\lambda_1 E_1^{(k)} + E_2^{(k)}},$$

where

$E_1^{(k)} = \hat{p}_m^{(k+1)}\hat{g}_0^{(k+1)} + 0.5\hat{g}_1^{(k+1)} + \hat{q}_m^{(k+1)}\hat{g}_2^{(k+1)}$,

$E_2^{(k)} = \hat{\lambda}_2^{(k)}\hat{p}_m^{(k+1)}\left(0.5\hat{g}_1^{(k+1)} + \hat{g}_2^{(k+1)}\right) + \hat{q}_m^{(k+1)}\left(\hat{g}_0^{(k+1)} + 0.5\hat{g}_1^{(k+1)}\right)$.

By solving the equation $\partial Q\left(\theta \middle| \hat{\theta}^{(k)}\right)/\partial\lambda_1 = 0$,

$$\hat{\lambda}_1^{(k+1)} = \frac{A_6^{(k)} E_2^{(k)}}{(N - A_6^{(k)})E_1^{(k)}}.$$

The first order partial derivative of the Q function (A1) with respect to λ_2 is

$$\frac{\partial Q\left(\theta \middle| \hat{\theta}^{(k)}\right)}{\partial\lambda_2} = \frac{A_7^{(k)}}{\lambda_2} - \frac{N F_1^{(k)}}{\lambda_2 F_1^{(k)} + F_2^{(k)}},$$

where

$$F_1^{(k)} = \hat{p}_m^{(k+1)}\left(0.5\hat{g}_1^{(k+1)} + \hat{g}_2^{(k+1)}\right),$$

$$F_2^{(k)} = \hat{\lambda}_1^{(k+1)}\left(\hat{p}_m^{(k+1)}\hat{g}_0^{(k+1)} + 0.5\hat{g}_1^{(k+1)} + \hat{q}_m^{(k+1)}\hat{g}_2^{(k+1)}\right) + \hat{q}_m^{(k+1)}\left(\hat{g}_0^{(k+1)} + 0.5\hat{g}_1^{(k+1)}\right).$$

By solving the equation $\partial Q\left(\theta \middle| \hat{\theta}^{(k)}\right)/\partial\lambda_2 = 0$,

$$\hat{\lambda}_2^{(k+1)} = \frac{A_7^{(k)} F_2^{(k)}}{(N - A_7^{(k)})F_1^{(k)}}.$$

ECM algorithm under the null hypothesis

Under the null hypothesis H_0: $\gamma = \gamma_0$, $\lambda_1 = \frac{\gamma_0(\lambda_2 - 1)}{2} + 1$. In the E-step at iteration (k+1), the Q function is given by

$$Q_0\left(\theta_0 \middle| \tilde{\theta}_0^{(k)}\right) = G_1^{(k)}\ln(1 - p_m) + G_2^{(k)}\ln p_m + G_3^{(k)}\ln g_0 + G_4^{(k)}\ln g_1 + G_5^{(k)}\ln(1 - g_0 - g_1) + G_6^{(k)}\ln\left[\frac{\gamma_0(\lambda_2 - 1)}{2} + 1\right] + G_7^{(k)}\ln\lambda_2 - N\ln R - E_{\tilde{\theta}_0^{(k)}}(N_{010} + N_{011} + N_{111} + N_{112})\ln 2, \qquad \text{(A2)}$$

where

$\tilde{\theta}_0^{(k)}$ is the MLE of θ_0 under H_0 at the iteration k,

$$G_1^{(k)} = E_{\tilde{\theta}_0^{(k)}}(N_{000} + N_{010} + N_{011} + N_{021}),$$

$G_2^{(k)} = E_{\tilde{\theta}_0^{(k)}}(N_{101} + N_{111} + N_{112} + N_{122}),$

$G_3^{(k)} = E_{\tilde{\theta}_0^{(k)}}(N_{000} + N_{101}),$

$G_4^{(k)} = E_{\tilde{\theta}_0^{(k)}}(N_{010} + N_{011} + N_{111} + N_{112}),$

$G_5^{(k)} = E_{\tilde{\theta}_0^{(k)}}(N_{021} + N_{122}),$

$G_6^{(k)} = E_{\tilde{\theta}_0^{(k)}}(N_{011} + N_{021} + N_{101} + N_{111}),$

$G_7^{(k)} = E_{\tilde{\theta}_0^{(k)}}(N_{112} + N_{122}).$

Under the null hypothesis, we only need to estimate p_m, g_0, g_1 and λ_2. The formulas of the MLEs of p_m, g_0 and g_1 at iteration (k+1) are similar to those under the alternative hypothesis, and thus we do not display them here for brevity. The MLE of λ_2 at iteration (k+1) is given as follows. The first order partial derivative of the Q function (A2) with respect to λ_2 under H_0 is

$$\frac{\partial Q_0\left(\theta_0 \middle| \tilde{\theta}_0^{(k)}\right)}{\partial \lambda_2} = \frac{G_7^{(k)}}{\lambda_2} + \frac{\gamma_0 G_6^{(k)}}{\gamma_0 \lambda_2 + 2 - \gamma_0} - \frac{N I_1^{(k)}}{\lambda_2 I_1^{(k)} + I_2^{(k)}},$$

where

$$I_1^{(k)} = 2\tilde{p}_m^{(k+1)}\left(0.5\tilde{g}_1^{(k+1)} + \tilde{g}_2^{(k+1)}\right)$$
$$+\gamma_0\left(\tilde{p}_m^{(k+1)}\tilde{g}_0^{(k+1)} + 0.5\tilde{g}_1^{(k+1)} + \tilde{q}_m^{(k+1)}\tilde{g}_2^{(k+1)}\right),$$

$$I_2^{(k)} = (2-\gamma_0)\left(\tilde{p}_m^{(k+1)}\tilde{g}_0^{(k+1)} + 0.5\tilde{g}_1^{(k+1)} + \tilde{q}_m^{(k+1)}\tilde{g}_2^{(k+1)}\right)$$
$$+2\tilde{q}_m^{(k+1)}\left(\tilde{g}_0^{(k+1)} + 0.5\tilde{g}_1^{(k+1)}\right).$$

$\tilde{\lambda}_2^{(k+1)}$ can be obtained by solving $\partial Q_0\left(\theta_0 \middle| \tilde{\theta}_0^{(k)}\right)/\partial \lambda_2 = 0$, i.e.,

$$\gamma_0\left(G_6^{(k)} + G_7^{(k)} - N\right)I_1^{(k)}[\tilde{\lambda}_2^{(k+1)}]^2 + [\gamma_0\left(G_6^{(k)} + G_7^{(k)}\right)I_2^{(k)} + (2 - \gamma_0)(G_7^{(k)} - N)I_1^{(k)}]\tilde{\lambda}_2^{(k+1)} + (2-\gamma_0)\, G_7^{(k)} I_2^{(k)} = 0.$$

Note that when there are more than one solutions to the above equation, we choose the one which is closer to $\tilde{\lambda}_2^{(k)}$.

Appendix C: Inapplicability of ECM algorithm when using only single daughters

When all the families are single daughters, $P(C|D) = \sum_{F\in\{0,1\}} \sum_{M\in\{0,1,2\}} P(FMC|D)$, and from Table 1, the observed log-likelihood function is

$$\begin{aligned}\ln L(\theta) = {} & n_{0,0}[\ln(1-p_m) + \ln(g_0 + 0.5g_1)] \\ & + n_{0,1}\{\ln[p_m g_0 + 0.5g_1 + (1-p_m)(1-g_0-g_1)] + \ln\lambda_1\} \\ & + n_{0,2}[\ln p_m + \ln(1-g_0-0.5g_1) + \ln\lambda_2] - N\ln R. \quad \text{(A3)}\end{aligned}$$

To obtain the MLE of θ, we take the first order partial derivative of Equation (A3) with respect to each element of θ as follows,

$$\frac{\partial \ln L(\theta)}{\partial p_m} = -\frac{n_{0,0}}{1-p_m} + \frac{n_{0,1}(2g_0+g_1-1)}{p_m g_0 + 0.5g_1 + (1-p_m)(1-g_0-g_1)} + \frac{n_{0,2}}{p_m} - \frac{N}{R}[g_0(\lambda_1-1) + 0.5g_1(\lambda_2-1) + (1-g_0-g_1)(\lambda_2-\lambda_1)] = 0,$$

$$\frac{\partial \ln L(\theta)}{\partial g_0} = \frac{n_{0,0}}{g_0+0.5g_1} + \frac{n_{0,1}(2p_m-1)}{p_m g_0 + 0.5g_1 + (1-p_m)(1-g_0-g_1)} - \frac{n_{0,2}}{1-g_0-0.5g_1} - \frac{N}{R}[(1-p_m)(1-\lambda_1) + p_m(\lambda_1-\lambda_2)] = 0,$$

$$\frac{\partial \ln L(\theta)}{\partial g_1} = \frac{0.5n_{0,0}}{g_0+0.5g_1} + \frac{n_{0,1}(p_m-0.5)}{p_m g_0 + 0.5g_1 + (1-p_m)(1-g_0-g_1)} - \frac{0.5n_{0,2}}{1-g_0-0.5g_1} - \frac{0.5N}{R}[(1-p_m)(1-\lambda_1) + p_m(\lambda_1-\lambda_2)] = 0,$$

$$\frac{\partial \ln L(\theta)}{\partial \lambda_1} = \frac{n_{0,1}}{\lambda_1} - \frac{N}{R}[p_m g_0 + 0.5g_1 + (1-p_m)(1-g_0-g_1)] = 0$$

and

$$\frac{\partial \ln L(\theta)}{\partial \lambda_2} = \frac{n_{0,2}}{\lambda_2} - \frac{N}{R}(1-g_0-0.5g_1)p_m = 0.$$

It is found that

$$\frac{\partial \ln L(\theta)}{\partial g_0} = 2\frac{\partial \ln L(\theta)}{\partial g_1}.$$

So, there is no unique solution to the above equation set.

Appendix D: Contribution of single daughters to estimate of θ in ECM algorithm

For simplicity, we only consider a sample consisting of case-parents trios with both parents and single daughters. Then, $A_1^{(k)}$ at iteration (k+1) in the ECM algorithm (see Additional file 1: Appendix B) can be written as follows:

$$\begin{aligned}A_1^{(k)} &= E_{\hat{\theta}^{(k)}}(N_{000} + N_{010} + N_{011} + N_{021}) \\ &= n_{000} + n_{010} + n_{011} + n_{021} + E_{\hat{\theta}^{(k)}}\big(z_{0,000}|n_{0,0}\big) + E_{\hat{\theta}^{(k)}}\big(z_{0,010}|n_{0,0}\big) \\ &\qquad + E_{\hat{\theta}^{(k)}}\big(z_{0,011}|n_{0,1}\big) + E_{\hat{\theta}^{(k)}}\big(z_{0,021}|n_{0,1}\big) \\ &= n_{000} + n_{010} + n_{011} + n_{021} + n_{0,0} + n_{0,1}\frac{\hat{q}_m^{(k)}(0.5\hat{g}_1^{(k)} + \hat{g}_2^{(k)})}{\hat{p}_m^{(k)}\hat{g}_0^{(k)} + 0.5\hat{g}_1^{(k)} + \hat{q}_m^{(k)}\hat{g}_2^{(k)}},\end{aligned}$$

where $E_{\hat{\theta}^{(k)}}\big(z_{0,FMC}|n_{0,C}\big)$'s are given in Additional file 1: Table S3. $A_2^{(k)}$ to $A_7^{(k)}$ can be derived in a way similar to $A_1^{(k)}$. Then,

$$A_2^{(k)} = n_{101} + n_{111} + n_{112} + n_{122} + n_{0,1}\frac{\hat{p}_m^{(k)}(\hat{g}_0^{(k)} + 0.5\hat{g}_1^{(k)})}{\hat{p}_m^{(k)}\hat{g}_0^{(k)} + 0.5\hat{g}_1^{(k)} + \hat{q}_m^{(k)}\hat{g}_2^{(k)}} + n_{0,2},$$

$$A_3^{(k)} = n_{000} + n_{101} + n_{0,0}\frac{\hat{g}_0^{(k)}}{\hat{g}_0^{(k)} + 0.5\hat{g}_1^{(k)}} + n_{0,1}\frac{\hat{p}_m^{(k)}\hat{g}_0^{(k)}\hat{\lambda}_1^{(k)}}{\hat{p}_m^{(k)}\hat{g}_0^{(k)} + 0.5\hat{g}_1^{(k)} + \hat{q}_m^{(k)}\hat{g}_2^{(k)}},$$

$$\begin{aligned}A_4^{(k)} &= n_{010} + n_{011} + n_{111} + n_{112} + n_{0,0}\frac{0.5\hat{g}_1^{(k)}}{\hat{g}_0^{(k)} + 0.5\hat{g}_1^{(k)}} \\ &\qquad + n_{0,1}\frac{0.5\hat{g}_1^{(k)}}{\hat{p}_m^{(k)}\hat{g}_0^{(k)} + 0.5\hat{g}_1^{(k)} + \hat{q}_m^{(k)}\hat{g}_2^{(k)}} + n_{0,2}\frac{0.5\hat{g}_1^{(k)}}{0.5\hat{g}_1^{(k)} + \hat{g}_2^{(k)}},\end{aligned}$$

$$A_5^{(k)} = n_{021} + n_{122} + n_{0,1}\frac{\hat{q}_m^{(k)}\hat{g}_2^{(k)}}{\hat{p}_m^{(k)}\hat{g}_0^{(k)} + 0.5\hat{g}_1^{(k)} + \hat{q}_m^{(k)}\hat{g}_2^{(k)}} + n_{0,2}\frac{\hat{g}_2^{(k)}}{0.5\hat{g}_1^{(k)} + \hat{g}_2^{(k)}},$$

$$A_6^{(k)} = n_{011} + n_{021} + n_{101} + n_{111} + n_{0,1}$$

and $A_7^{(k)} = n_{112} + n_{122} + n_{0,2}$.

On the other hand, since the MLE of p_m at iteration (k+1) involves the values of

$A_1^{(k)}$ and $A_2^{(k)}$ (see Additional file 1: Appendix B), i.e.,

$$\hat{p}_m^{(k+1)} = \frac{A_2^{(k)} B_2^{(k)}}{A_1^{(k)} B_1^{(k)} + N B_2^{(k)}},$$

the numbers of case-parents trios with both parents of eight types (n_{000}, n_{010}, n_{011}, n_{021}, n_{101}, n_{111}, n_{112} and n_{122}) and single daughters of three types ($n_{0,0}$, $n_{0,1}$ and $n_{0,2}$) in $A_1^{(k)}$ and $A_2^{(k)}$ can contribute to $\hat{p}_m^{(k+1)}$. Likewise, the numbers of case-parents trios and single daughters in $A_2^{(k)}$ to $A_7^{(k)}$ can contribute to $\hat{g}_0^{(k+1)}$, $\hat{g}_1^{(k+1)}$, $\hat{\lambda}_1^{(k+1)}$ and $\hat{\lambda}_2^{(k+1)}$, respectively.

Appendix E: Effect of different initial values of $\boldsymbol{\theta}$ ($\boldsymbol{\theta_0}$) on ECM algorithm

Note that the ECM algorithm can converge to a local maximum of the log-likelihood function instead of a global maximum. To investigate this, we randomly choose 1000 initial values of θ (θ_0) from the parameter space and regard the MLE of θ (θ_0) with the maximum log-likelihood among 1000 $\ln L(\hat{\theta})$'s ($\ln L(\tilde{\theta}_0)$'s) as the global MLE of θ (θ_0). The corresponding ECM algorithm is denoted by ECM_{1000}. For easy comparison with ECM_{1000}, the proposed ECM algorithm based on the initial value estimated by the method described in Additional file 1: Appendix B is denoted by ECM_1. As such, if the absolute difference of $\hat{\theta}$ or $\ln L(\hat{\theta})$ ($\tilde{\theta}_0$ or $\ln L(\tilde{\theta}_0)$) between ECM_1 and ECM_{1000} is small, then ECM_1 may converge towards the global maximum. We conduct a simulation study under the simulation settings with $\rho = 0$, $\lambda_2 = 1.5$ and (p_m, p_f) = (0.30, 0.30). We calculate the averages of the absolute differences of $\hat{\theta}$'s ($\ln L(\hat{\theta})$'s) between ECM_1 and ECM_{1000} based on 100 replicates, and those of $\tilde{\theta}_0$'s ($\ln L(\tilde{\theta}_0)$'s), which are given in Tables S10 and S11, respectively. Here, $\overline{\Delta\hat{p}_m}$, $\overline{\Delta\hat{g}_0}$, $\overline{\Delta\hat{g}_1}$, $\overline{\Delta\hat{\lambda}_1}$, $\overline{\Delta\hat{\lambda}_2}$, $\overline{\Delta\ln L(\hat{\theta})}$ and $\overline{\Delta\ln L(\tilde{\theta}_0)}$ denote the averages of the absolute differences of $\hat{p}_m$, $\hat{g}_0$, $\hat{g}_1$, $\hat{\lambda}_1$, $\hat{\lambda}_2$, $\ln L(\hat{\theta})$ and $\ln L(\tilde{\theta}_0)$ between two methods over 100 replicates, respectively. The simulation results show that the values of $\hat{\theta}$ and $\ln L(\hat{\theta})$ ($\tilde{\theta}_0$ and $\ln L(\tilde{\theta}_0)$) based on one initial value estimated by the method described in Additional file 1: Appendix B are very close to those based on 1000 initial values under all the simulated situations when N_2 (the number of complete family trios) is not too small, such as MP1 and MP2, which may indicate

that the ECM algorithm based on the estimated initial value converges towards the global maximum. As for MP3-MP6, except that $\tilde{\theta}_0$ with $(\gamma_0,\ \gamma) = (1, 2)$ under MP5 and MP6, $\tilde{\theta}_0$ with $(\gamma_0,\ \gamma) = (1, 1)$ and $(1, 2)$ under MP3, and $\tilde{\theta}_0$ with $(\gamma_0,\ \gamma) = (1, 1)$, $(1, 1.5)$ and $(1, 2)$ under MP4 may converge to a local maximum, all the other $\hat{\theta}$ and $\tilde{\theta}_0$ results converge to the global maximum. Further, for these seven cases, we try and randomly select ten groups of initial values of θ_0 from the parameter space and regard $\tilde{\theta}_0$ with the maximum log-likelihood among ten $\ln L(\tilde{\theta}_0)$'s as the final MLE of θ_0. The corresponding ECM is denoted by ECM_{10}. We find that $\tilde{\theta}_0$'s based on ten and 1000 initial values are very close to each other under all the seven simulated situations (see Table S11).

Table S1 Seven types of possible mother-daughter pairs, and the corresponding conditional probabilities and conditional expectations

MC	$F\|MC$	$z_{1m,FMC}$	$n_{1m,MC}$	$P(F\|MC,D)$	$E(z_{1m,FMC}\|n_{1m,MC})$
00	0	$z_{1m,000}$	$n_{1m,00} = z_{1m,000}$	1	$n_{1m,00}$
01	1	$z_{1m,101}$	$n_{1m,01} = z_{1m,101}$	1	$n_{1m,01}$
10	0	$z_{1m,010}$	$n_{1m,10} = z_{1m,010}$	1	$n_{1m,10}$
11	$\begin{cases}0\\1\end{cases}$	$z_{1m,011}$<br>$z_{1m,111}$	$n_{1m,11} = z_{1m,011} + z_{1m,111}$	q_m<br>p_m	$n_{1m,11}q_m$<br>$n_{1m,11}p_m$
12	1	$z_{1m,112}$	$n_{1m,12} = z_{1m,112}$	1	$n_{1m,12}$
21	0	$z_{1m,021}$	$n_{1m,21} = z_{1m,021}$	1	$n_{1m,21}$
22	1	$z_{1m,122}$	$n_{1m,22} = z_{1m,122}$	1	$n_{1m,22}$

Table S2 Four types of possible father-daughter pairs, and the corresponding conditional probabilities and conditional expectations

FC	$M\|FC$	$z_{1f,FMC}$	$n_{1f,FC}$	$P(M\|FC,D)$	$E(z_{1f,FMC}\|n_{1f,FC})$
00	0	$z_{1f,000}$	$n_{1f,00}=z_{1f,000}+z_{1f,010}$	$g_0/(g_0+0.5g_1)$	$n_{1f,00}g_0/(g_0+0.5g_1)$
	1	$z_{1f,010}$		$0.5g_1/(g_0+0.5g_1)$	$0.5n_{1f,00}g_1/(g_0+0.5g_1)$
01	1	$z_{1f,011}$	$n_{1f,01}=z_{1f,011}+z_{1f,021}$	$0.5g_1/(0.5g_1+g_2)$	$0.5n_{1f,01}g_1/(0.5g_1+g_2)$
	2	$z_{1f,021}$		$g_2/(0.5g_1+g_2)$	$n_{1f,01}g_2/(0.5g_1+g_2)$
11	0	$z_{1f,101}$	$n_{1f,11}=z_{1f,101}+z_{1f,111}$	$g_0/(g_0+0.5g_1)$	$n_{1f,11}g_0/(g_0+0.5g_1)$
	1	$z_{1f,111}$		$0.5g_1/(g_0+0.5g_1)$	$0.5n_{1f,11}g_1/(g_0+0.5g_1)$
12	1	$z_{1f,112}$	$n_{1f,12}=z_{1f,112}+z_{1f,122}$	$0.5g_1/(0.5g_1+g_2)$	$0.5n_{1f,12}g_1/(0.5g_1+g_2)$
	2	$z_{1f,122}$		$g_2/(0.5g_1+g_2)$	$n_{1f,12}g_2/(0.5g_1+g_2)$

Table S3 Three types of possible single daughters, and the corresponding conditional probabilities and conditional expectations

C	$FM\|C$	$z_{0,FMC}$	$n_{0,C}$	$P(FM\|C,D)$	$E(z_{0,FMC}\|n_{0,C})$
0	00	$z_{0,000}$	$n_{0,0}=z_{0,000}+z_{0,010}$	$g_0/(g_0+0.5g_1)$	$n_{0,0}g_0/(g_0+0.5g_1)$
	01	$z_{0,010}$		$0.5g_1/(g_0+0.5g_1)$	$0.5n_{0,0}g_1/(g_0+0.5g_1)$
1	01	$z_{0,011}$	$n_{0,1}=z_{0,011}+z_{0,021}$ $+z_{0,101}+z_{0,111}$	$0.5q_mg_1/(p_mg_0+0.5g_1+q_mg_2)$	$0.5n_{0,1}q_mg_1/(p_mg_0+0.5g_1+q_mg_2)$
	02	$z_{0,021}$		$q_mg_2/(p_mg_0+0.5g_1+q_mg_2)$	$n_{0,1}q_mg_2/(p_mg_0+0.5g_1+q_mg_2)$
	10	$z_{0,101}$		$p_mg_0/(p_mg_0+0.5g_1+q_mg_2)$	$n_{0,1}p_mg_0/(p_mg_0+0.5g_1+q_mg_2)$
	11	$z_{0,111}$		$0.5p_mg_1/(p_mg_0+0.5g_1+q_mg_2)$	$0.5n_{0,1}p_mg_1/(p_mg_0+0.5g_1+q_mg_2)$
2	11	$z_{0,112}$	$n_{0,2}=z_{0,112}+z_{0,122}$	$0.5g_1/(0.5g_1+g_2)$	$0.5n_{0,2}g_1/(0.5g_1+g_2)$
	12	$z_{0,122}$		$g_2/(0.5g_1+g_2)$	$g_2n_{0,2}/(0.5g_1+g_2)$

Table S4 Statistical properties of likelihood-based confidence interval of γ against missing pattern (MP) and γ with $\rho = 0.05$, $\lambda_2 = 1.5$, and $(p_m,\ p_f)$ being (0.30, 0.30), (0.25, 0.30) and (0.30, 0.25)[a]

		$(p_m,\ p_f)$ = (0.30, 0.30)			$(p_m,\ p_f)$ = (0.25, 0.30)			$(p_m,\ p_f)$ = (0.30, 0.25)		
MP	γ	CP (%)	ML/(ML +MR)	DP	CP (%)	ML/(ML +MR)	DP	CP (%)	ML/(ML +MR)	DP
1	0	94.50	1	0.103	94.57	1	0.106	94.67	1	0.111
	0.5	94.53	0.53	0.037	94.62	0.51	0.033	95.12	0.55	0.038
	1	94.70	0.30	0.021	94.64	0.33	0.024	95.01	0.34	0.026
	1.5	94.90	0.24	0.058	94.70	0.23	0.060	95.01	0.26	0.066
	2	94.89	0	0.067	95.06	0	0.064	95.36	0	0.074
2	0	95.12	1	0.175	95.15	1	0.175	94.78	1	0.176
	0.5	95.34	0.56	0.067	95.30	0.53	0.055	95.17	0.65	0.066
	1	94.81	0.16	0.036	94.69	0.20	0.041	95.00	0.18	0.039
	1.5	94.83	0.12	0.113	94.84	0.12	0.112	94.59	0.09	0.121
	2	95.06	0	0.145	95.15	0	0.132	94.66	0	0.154
3	0	94.88	1	0.395	95.08	1	0.368	94.86	1	0.382
	0.5	95.66	0.76	0.145	95.86	0.67	0.125	95.64	0.78	0.146
	1	95.35	0.02	0.068	95.52	0	0.072	95.53	0.03	0.070
	1.5	94.72	0	0.237	95.04	0	0.234	95.05	0	0.219
	2	94.49	0	0.426	94.99	0	0.405	94.87	0	0.404
4	0	94.27	1	0.520	94.70	1	0.482	94.57	1	0.496
	0.5	95.00	0.91	0.190	95.42	0.83	0.162	95.24	0.90	0.176
	1	94.90	0.05	0.068	95.01	0.03	0.070	94.15	0.04	0.071
	1.5	94.91	0	0.218	94.89	0	0.228	94.94	0	0.191
	2	94.50	0	0.444	94.69	0	0.453	94.63	0	0.396
5	0	94.94	1	0.230	94.88	1	0.219	95.04	1	0.229
	0.5	95.42	0.64	0.086	95.52	0.55	0.069	95.81	0.71	0.084
	1	95.45	0.11	0.051	95.11	0.11	0.055	95.79	0.09	0.046
	1.5	94.82	0.05	0.157	95.08	0.04	0.160	94.85	0.04	0.156
	2	95.18	0	0.237	94.97	0	0.204	94.84	0	0.246
6	0	95.20	1	0.314	95.12	1	0.285	94.78	1	0.312
	0.5	95.21	0.72	0.118	95.26	0.66	0.104	95.88	0.77	0.112
	1	95.17	0.06	0.061	95.31	0.03	0.065	95.28	0.02	0.059
	1.5	94.82	0.01	0.205	95.06	0.02	0.193	94.73	0.01	0.192
	2	94.90	0	0.313	94.49	0	0.293	94.76	0	0.314

[a]The simulations are conducted under 10,000 replicates and 5% significance level

Table S5 Statistical properties of likelihood-based confidence interval of γ against missing pattern (MP) and γ with $\rho = 0.05$, $\lambda_2 = 2$, and (p_m, p_f) being (0.30, 0.30), (0.25, 0.30) and (0.30, 0.25)[a]

		(p_m, p_f) = (0.30, 0.30)			(p_m, p_f) = (0.25, 0.30)			(p_m, p_f) = (0.30, 0.25)		
MP	γ	CP (%)	ML/(ML +MR)	DP	CP (%)	ML/(ML +MR)	DP	CP (%)	ML/(ML +MR)	DP
1	0	94.60	0.92	0.027	94.44	0.91	0.033	94.31	0.94	0.033
	0.5	94.70	0.38	0.007	94.98	0.40	0.007	94.95	0.41	0.009
	1	94.82	0.42	0.004	94.86	0.40	0.006	95.00	0.41	0.007
	1.5	95.05	0.42	0.006	95.00	0.44	0.006	95.01	0.43	0.009
	2	95.01	0.02	0.025	95.06	0	0.024	95.26	0	0.023
2	0	95.13	0.99	0.036	95.09	1	0.044	94.63	1	0.044
	0.5	95.27	0.40	0.031	95.16	0.35	0.024	95.11	0.38	0.030
	1	94.78	0.35	0.016	95.08	0.37	0.020	95.22	0.29	0.024
	1.5	94.76	0.42	0.032	95.17	0.41	0.026	95.02	0.39	0.039
	2	95.00	0	0.027	94.78	0	0.028	95.16	0	0.028
3	0	94.95	1	0.188	95.11	1	0.209	95.16	1	0.197
	0.5	95.41	0.32	0.176	96.00	0.31	0.143	95.12	0.38	0.177
	1	95.52	0.01	0.109	95.56	0.02	0.112	95.47	0.01	0.097
	1.5	95.02	0.01	0.314	95.24	0.02	0.287	94.62	0.01	0.326
	2	94.96	0	0.213	94.73	0	0.169	95.25	0	0.248
4	0	94.52	1	0.414	94.50	1	0.431	94.47	1	0.426
	0.5	95.04	0.49	0.276	94.64	0.36	0.239	94.86	0.49	0.276
	1	95.27	0.01	0.125	95.26	0	0.149	95.40	0.01	0.105
	1.5	94.82	0	0.459	94.74	0	0.465	94.60	0	0.404
	2	94.37	0	0.459	94.81	0	0.433	94.36	0	0.482
5	0	94.93	1	0.053	95.07	1	0.066	94.92	1	0.061
	0.5	95.00	0.37	0.061	95.15	0.34	0.050	95.48	0.39	0.063
	1	95.05	0.22	0.044	94.95	0.25	0.049	95.47	0.21	0.042
	1.5	94.74	0.21	0.093	94.76	0.29	0.076	94.68	0.21	0.105
	2	94.87	0	0.042	94.79	0	0.033	94.76	0	0.056
6	0	95.24	1	0.097	94.97	1	0.117	95.06	1	0.115
	0.5	95.86	0.36	0.106	95.38	0.35	0.090	95.39	0.31	0.105
	1	95.23	0.09	0.072	95.14	0.10	0.073	95.04	0.07	0.066
	1.5	94.73	0.09	0.156	94.95	0.11	0.141	95.23	0.11	0.174
	2	95.06	0	0.069	94.63	0	0.064	95.01	0	0.093

[a]The simulations are conducted under 10,000 replicates and 5% significance level

Table S6 Statistical properties of likelihood-based confidence interval of γ against missing pattern (MP) and γ with $\rho = 0$, $\lambda_2 = 1.5$, and (p_m, p_f) being (0.20, 0.20), (0.15, 0.20) and (0.20, 0.15)[a]

		(p_m, p_f) = (0.20, 0.20)			(p_m, p_f) = (0.15, 0.20)			(p_m, p_f) = (0.20, 0.15)		
MP	γ	CP (%)	ML/(ML +MR)	DP	CP (%)	ML/(ML +MR)	DP	CP(%)	ML/(ML +MR)	DP
1	0	94.95	1	0.103	94.97	1	0.094	94.82	1	0.104
	0.5	95.36	0.50	0.028	95.34	0.45	0.026	95.09	0.52	0.032
	1	94.92	0.24	0.029	94.82	0.26	0.032	94.83	0.23	0.034
	1.5	95.00	0.20	0.066	95.01	0.21	0.060	94.73	0.16	0.075
	2	95.10	0	0.074	94.85	0	0.064	95.03	0	0.086
2	0	94.88	1	0.150	94.94	1	0.129	94.88	1	0.136
	0.5	94.72	0.51	0.048	95.34	0.53	0.034	95.31	0.59	0.045
	1	94.93	0.19	0.043	94.98	0.16	0.046	95.21	0.18	0.042
	1.5	94.58	0.10	0.107	95.01	0.11	0.101	95.14	0.07	0.103
	2	94.82	0	0.135	94.99	0	0.123	94.82	0	0.145
3	0	95.00	1	0.278	94.98	1	0.219	94.79	1	0.243
	0.5	95.72	0.65	0.094	95.67	0.53	0.068	95.70	0.77	0.088
	1	95.58	0.02	0.071	95.01	0.02	0.069	95.13	0.03	0.066
	1.5	95.09	0	0.181	94.96	0.01	0.177	94.69	0	0.169
	2	95.18	0	0.334	94.89	0	0.292	94.74	0	0.302
4	0	94.93	1	0.365	94.92	1	0.304	94.84	1	0.327
	0.5	95.21	0.78	0.130	95.37	0.69	0.105	95.40	0.80	0.129
	1	94.78	0.08	0.070	95.17	0.03	0.073	93.67	0.11	0.083
	1.5	94.94	0	0.179	95.01	0	0.187	95.28	0	0.189
	2	94.82	0	0.373	94.87	0	0.356	95.01	0	0.366
5	0	94.75	1	0.173	95.10	1	0.145	94.96	1	0.154
	0.5	95.58	0.61	0.052	95.15	0.52	0.045	95.78	0.61	0.052
	1	95.56	0.13	0.047	95.11	0.13	0.049	95.17	0.12	0.046
	1.5	95.06	0.06	0.128	94.79	0.08	0.121	95.26	0.05	0.110
	2	94.96	0	0.190	95.01	0	0.152	95.10	0	0.185
6	0	94.68	1	0.221	94.92	1	0.179	94.77	1	0.207
	0.5	95.76	0.63	0.077	95.18	0.59	0.062	95.62	0.64	0.074
	1	95.01	0.04	0.058	95.24	0.06	0.061	95.10	0.03	0.060
	1.5	94.69	0.02	0.167	95.04	0.02	0.159	94.79	0.01	0.156
	2	94.55	0	0.270	94.94	0	0.239	94.95	0	0.266

[a]The simulations are conducted under 10,000 replicates and 5% significance level

Table S7 Statistical properties of likelihood-based confidence interval of γ against missing pattern (MP) and γ with $\rho = 0$, $\lambda_2 = 2$, and (p_m, p_f) being (0.20, 0.20), (0.15, 0.20) and (0.20, 0.15)[a]

		(p_m, p_f) = (0.20, 0.20)			(p_m, p_f) = (0.15, 0.20)			(p_m, p_f) = (0.20, 0.15)		
MP	γ	CP (%)	ML/(ML +MR)	DP	CP (%)	ML/(ML +MR)	DP	CP(%)	ML/(ML +MR)	DP
1	0	94.62	0.96	0.049	95.02	0.95	0.058	94.67	0.98	0.062
	0.5	94.88	0.38	0.008	95.17	0.36	0.007	95.00	0.39	0.013
	1	95.22	0.40	0.010	94.85	0.42	0.010	94.97	0.36	0.013
	1.5	95.00	0.45	0.010	94.87	0.47	0.010	94.76	0.34	0.015
	2	94.87	0	0.028	95.01	0	0.027	94.94	0	0.025
2	0	94.82	1	0.068	95.13	0.99	0.091	94.84	1	0.092
	0.5	95.17	0.38	0.024	95.09	0.34	0.021	94.98	0.37	0.031
	1	94.68	0.31	0.028	95.29	0.32	0.030	94.91	0.28	0.034
	1.5	94.70	0.38	0.032	94.94	0.35	0.028	94.71	0.33	0.055
	2	94.44	0	0.034	95.00	0	0.028	94.55	0	0.040
3	0	94.87	1	0.229	94.71	1	0.239	95.04	1	0.256
	0.5	95.45	0.30	0.113	95.54	0.25	0.080	95.49	0.34	0.110
	1	95.17	0.03	0.085	95.58	0.04	0.088	94.79	0.03	0.082
	1.5	95.08	0.03	0.244	95.03	0.05	0.208	94.78	0.01	0.254
	2	95.21	0	0.185	95.31	0	0.140	94.68	0	0.250
4	0	94.73	1	0.437	94.81	1	0.421	94.77	1	0.459
	0.5	94.70	0.42	0.187	94.27	0.37	0.137	94.79	0.44	0.176
	1	94.99	0.01	0.099	95.17	0.01	0.110	93.50	0.11	0.096
	1.5	94.55	0	0.346	94.98	0	0.340	94.87	0	0.352
	2	94.58	0	0.410	95.04	0	0.374	94.90	0	0.472
5	0	94.98	1	0.090	95.09	1	0.109	95.30	1	0.112
	0.5	95.29	0.35	0.036	95.54	0.39	0.026	95.73	0.37	0.038
	1	94.89	0.24	0.041	95.02	0.31	0.042	95.37	0.22	0.041
	1.5	94.92	0.27	0.070	94.97	0.29	0.051	94.57	0.23	0.093
	2	95.10	0	0.042	95.34	0	0.034	94.86	0	0.062
6	0	94.77	1	0.158	94.95	1	0.172	94.81	1	0.194
	0.5	95.71	0.24	0.075	95.50	0.29	0.054	95.86	0.28	0.079
	1	95.11	0.10	0.065	94.99	0.13	0.067	94.97	0.06	0.073
	1.5	94.72	0.10	0.142	94.71	0.16	0.130	94.70	0.08	0.183
	2	94.49	0	0.075	94.67	0	0.069	94.92	0	0.122

[a]The simulations are conducted under 10,000 replicates and 5% significance level

Table S8 Statistical properties of likelihood-based confidence interval of γ against missing pattern (MP) and γ with $\rho = 0.05$, $\lambda_2 = 1.5$, and $(p_m,\ p_f)$ being (0.20, 0.20), (0.15, 0.20) and (0.20, 0.15)[a]

		$(p_m,\ p_f)$ = (0.20, 0.20)			$(p_m,\ p_f)$ = (0.15, 0.20)			$(p_m,\ p_f)$ = (0.20, 0.15)		
MP	γ	CP (%)	ML/(ML +MR)	DP	CP (%)	ML/(ML +MR)	DP	CP(%)	ML/(ML +MR)	DP
1	0	94.85	1	0.106	94.92	1	0.097	94.95	1	0.106
	0.5	95.26	0.49	0.033	95.30	0.44	0.028	95.24	0.53	0.036
	1	95.20	0.26	0.030	94.79	0.28	0.035	94.84	0.22	0.034
	1.5	94.94	0.24	0.067	95.00	0.23	0.065	95.00	0.17	0.078
	2	94.88	0	0.081	94.94	0	0.070	94.72	0	0.096
2	0	94.90	1	0.158	94.87	1	0.137	95.17	1	0.144
	0.5	95.16	0.53	0.051	95.27	0.53	0.039	95.37	0.60	0.049
	1	94.94	0.15	0.044	95.04	0.14	0.048	95.17	0.15	0.046
	1.5	94.58	0.09	0.108	95.12	0.12	0.109	94.90	0.07	0.109
	2	94.83	0	0.151	95.20	0	0.128	94.74	0	0.157
3	0	94.87	1	0.284	95.01	1	0.234	94.90	1	0.254
	0.5	95.59	0.71	0.101	95.58	0.57	0.077	95.58	0.75	0.093
	1	95.45	0.02	0.074	95.20	0.03	0.072	95.42	0.04	0.067
	1.5	94.98	0	0.195	95.10	0	0.184	94.52	0	0.170
	2	94.80	0	0.347	95.09	0	0.314	94.77	0	0.322
4	0	94.89	1	0.383	94.91	1	0.310	94.87	1	0.331
	0.5	95.18	0.80	0.134	95.16	0.69	0.106	95.44	0.80	0.128
	1	94.93	0.05	0.071	94.86	0.05	0.074	93.58	0.12	0.088
	1.5	94.81	0	0.184	94.95	0	0.192	95.21	0	0.194
	2	94.80	0	0.374	94.94	0	0.361	94.64	0	0.370
5	0	95.15	1	0.179	95.26	1	0.152	94.66	1	0.162
	0.5	95.45	0.65	0.055	95.68	0.50	0.045	95.51	0.61	0.056
	1	95.42	0.12	0.052	95.19	0.13	0.052	95.55	0.08	0.049
	1.5	94.77	0.08	0.131	95.04	0.09	0.122	95.10	0.04	0.116
	2	94.96	0	0.199	94.84	0	0.157	95.06	0	0.201
6	0	95.03	1	0.242	94.97	1	0.194	94.92	1	0.219
	0.5	95.83	0.56	0.081	95.49	0.58	0.067	95.53	0.65	0.080
	1	95.28	0.03	0.063	95.30	0.06	0.062	95.41	0.04	0.064
	1.5	94.76	0	0.167	95.03	0.02	0.168	95.13	0	0.157
	2	94.54	0	0.292	95.05	0	0.257	94.75	0	0.277

[a]The simulations are conducted under 10,000 replicates and 5% significance level

Table S9 Statistical properties of likelihood-based confidence interval of γ against missing pattern (MP) and γ with $\rho = 0.05$, $\lambda_2 = 2$, and $(p_m,\ p_f)$ being (0.20, 0.20), (0.15, 0.20) and (0.20, 0.15)[a]

		$(p_m,\ p_f)$ = (0.20, 0.20)			$(p_m,\ p_f)$ = (0.15, 0.20)			$(p_m,\ p_f)$ = (0.20, 0.15)		
MP	γ	CP (%)	ML/(ML +MR)	DP	CP (%)	ML/(ML +MR)	DP	CP(%)	ML/(ML +MR)	DP
1	0	94.38	0.98	0.052	94.60	0.97	0.065	94.81	0.99	0.065
	0.5	94.96	0.38	0.010	95.10	0.41	0.010	95.06	0.39	0.014
	1	95.13	0.46	0.012	95.32	0.48	0.015	95.11	0.38	0.015
	1.5	95.07	0.44	0.011	94.92	0.46	0.010	94.72	0.35	0.018
	2	94.67	0	0.027	94.96	0	0.025	94.89	0	0.025
2	0	94.68	1	0.078	94.71	1	0.096	95.31	1	0.102
	0.5	94.95	0.38	0.030	95.07	0.37	0.021	95.28	0.39	0.034
	1	94.80	0.31	0.032	95.07	0.33	0.033	95.19	0.24	0.036
	1.5	94.64	0.36	0.041	95.08	0.32	0.034	94.83	0.27	0.061
	2	94.83	0	0.035	94.71	0	0.031	94.67	0	0.043
3	0	94.88	1	0.251	94.93	1	0.256	94.91	1	0.283
	0.5	95.07	0.27	0.123	95.40	0.23	0.082	95.75	0.34	0.118
	1	94.91	0.02	0.091	95.29	0.03	0.097	94.60	0.04	0.089
	1.5	95.01	0.01	0.271	95.34	0.03	0.230	94.75	0.01	0.276
	2	95.13	0	0.214	95.16	0	0.165	94.53	0	0.290
4	0	94.74	1	0.466	94.91	1	0.446	94.72	1	0.479
	0.5	94.49	0.41	0.198	94.51	0.35	0.145	94.55	0.46	0.184
	1	94.92	0.03	0.098	95.25	0.03	0.109	93.19	0.14	0.101
	1.5	94.79	0	0.354	94.93	0	0.356	95.08	0	0.362
	2	94.23	0	0.438	94.81	0	0.396	94.88	0	0.497
5	0	95.06	1	0.099	95.16	1	0.113	94.89	1	0.124
	0.5	95.17	0.37	0.040	95.42	0.38	0.029	95.60	0.40	0.047
	1	94.84	0.24	0.045	94.96	0.27	0.047	95.16	0.20	0.045
	1.5	94.82	0.24	0.082	94.95	0.27	0.058	94.74	0.20	0.103
	2	94.83	0	0.049	95.25	0	0.034	95.01	0	0.071
6	0	94.98	1	0.181	95.01	1	0.192	95.03	1	0.216
	0.5	95.67	0.27	0.087	95.62	0.27	0.062	95.73	0.31	0.090
	1	94.92	0.06	0.071	94.99	0.08	0.075	95.11	0.06	0.074
	1.5	94.40	0.09	0.173	94.89	0.13	0.151	94.69	0.06	0.204
	2	94.44	0	0.096	94.47	0	0.085	94.76	0	0.148

[a]The simulations are conducted under 10,000 replicates and 5% significance level

Table S10 Averages of absolute differences of each element of $\hat{\theta}$ and $\ln L(\hat{\theta})$ between ECM_1 and ECM_{1000} with $\rho = 0$, $\lambda_2 =$ 1.5 and (p_m, p_f) = (0.30, 0.30) under MP1-MP6

Missing pattern	γ	$\overline{\Delta\hat{p}_m}$	$\overline{\Delta\hat{g}_0}$	$\overline{\Delta\hat{g}_1}$	$\overline{\Delta\hat{\lambda}_1}$	$\overline{\Delta\hat{\lambda}_2}$	$\overline{\Delta\ln L(\hat{\theta})}$
MP1	0	6.73E-08	6.63E-08	4.39E-08	2.82E-07	8.02E-07	7.25E-13
	0.5	6.94E-08	7.20E-08	4.84E-08	3.37E-07	8.63E-07	1.01E-12
	1	6.79E-08	7.08E-08	4.75E-08	3.59E-07	8.12E-07	6.96E-13
	1.5	6.94E-08	7.64E-08	5.26E-08	4.23E-07	8.98E-07	6.98E-13
	2	6.54E-08	7.13E-08	4.90E-08	4.21E-07	8.01E-07	7.19E-13
MP2	0	1.08E-07	1.05E-07	6.74E-08	4.42E-07	1.29E-06	7.34E-13
	0.5	1.31E-07	1.31E-07	8.55E-08	6.00E-07	1.57E-06	7.25E-13
	1	1.13E-07	1.18E-07	7.89E-08	6.04E-07	1.43E-06	7.53E-13
	1.5	1.04E-07	1.10E-07	7.35E-08	6.04E-07	1.27E-06	6.98E-13
	2	1.22E-07	1.34E-07	9.23E-08	8.14E-07	1.64E-06	7.28E-13
MP3	0	3.37E-07	3.21E-07	1.98E-07	1.33E-06	4.08E-06	1.17E-12
	0.5	3.69E-07	3.60E-07	2.27E-07	1.67E-06	4.71E-06	8.69E-13
	1	4.05E-07	4.06E-07	2.63E-07	2.04E-06	5.27E-06	9.03E-13
	1.5	3.98E-07	4.08E-07	2.61E-07	2.26E-06	5.11E-06	8.53E-13
	2	4.20E-07	4.26E-07	2.71E-07	2.50E-06	5.22E-06	7.98E-13
MP4	0	6.68E-07	6.63E-07	4.29E-07	2.89E-06	1.01E-05	1.63E-12
	0.5	7.32E-07	6.92E-07	4.31E-07	3.14E-06	8.50E-06	2.04E-12
	1	1.93E-06	2.76E-06	2.30E-06	4.41E-05	2.98E-04	2.66E-08
	1.5	1.98E-06	2.77E-06	2.29E-06	4.37E-05	2.69E-04	2.31E-08
	2	1.87E-06	2.52E-06	2.05E-06	3.73E-05	1.85E-04	1.49E-08
MP5	0	1.38E-07	1.10E-07	7.23E-08	5.52E-07	1.64E-06	7.25E-13
	0.5	1.49E-07	1.20E-07	7.81E-08	6.59E-07	1.74E-06	7.09E-13
	1	1.80E-07	1.43E-07	9.03E-08	8.16E-07	1.93E-06	6.62E-13
	1.5	2.10E-07	1.73E-07	1.14E-07	1.09E-06	2.35E-06	7.66E-13
	2	1.66E-07	1.41E-07	9.22E-08	9.59E-07	1.92E-06	7.55E-13
MP6	0	2.76E-07	3.39E-07	2.24E-07	1.27E-06	4.00E-06	7.45E-13
	0.5	2.50E-07	3.12E-07	2.05E-07	1.26E-06	3.41E-06	7.98E-13
	1	2.14E-07	2.69E-07	1.81E-07	1.25E-06	3.24E-06	8.57E-13
	1.5	2.56E-07	3.20E-07	2.09E-07	1.59E-06	3.58E-06	8.07E-13
	2	3.07E-07	3.87E-07	2.57E-07	2.02E-06	4.10E-06	8.44E-13

Table S11 Averages of absolute differences of each element of $\tilde{\theta}_0$ and $\ln L(\tilde{\theta}_0)$ between ECM_1/ECM_{10} and ECM_{1000} with $\rho = 0$, $\lambda_2 = 1.5$ and (p_m, p_f) = (0.30, 0.30) under MP1-MP6

Missing pattern	Methods	γ_0	γ	$\overline{\Delta\tilde{p}_m}$	$\overline{\Delta\tilde{g}_0}$	$\overline{\Delta\tilde{g}_1}$	$\overline{\Delta\tilde{\lambda}_2}$	$\overline{\Delta\ln L(\tilde{\theta}_0)}$
MP1	ECM_1 vs. ECM_{1000}	0	0	2.45E-08	8.74E-08	6.81E-08	4.32E-07	1.39E-12
	ECM_1 vs. ECM_{1000}		0.5	2.24E-08	7.37E-08	5.65E-08	3.11E-07	1.39E-12
	ECM_1 vs. ECM_{1000}		1	1.74E-08	5.22E-08	3.92E-08	1.92E-07	1.63E-12
	ECM_1 vs. ECM_{1000}		1.5	1.68E-08	4.80E-08	3.56E-08	1.56E-07	1.79E-12
	ECM_1 vs. ECM_{1000}		2	1.37E-08	3.50E-08	2.51E-08	1.00E-07	1.68E-12
	ECM_1 vs. ECM_{1000}	1	0	5.52E-08	6.82E-08	4.71E-08	5.27E-07	9.46E-13
	ECM_1 vs. ECM_{1000}		0.5	4.42E-08	5.13E-08	3.50E-08	4.57E-07	8.53E-13
	ECM_1 vs. ECM_{1000}		1	5.93E-08	6.74E-08	4.66E-08	6.73E-07	7.94E-13
	ECM_1 vs. ECM_{1000}		1.5	5.33E-08	6.19E-08	4.35E-08	6.95E-07	6.59E-13
	ECM_1 vs. ECM_{1000}		2	5.49E-08	6.36E-08	4.52E-08	7.91E-07	6.34E-13
	ECM_1 vs. ECM_{1000}	2	0	6.30E-08	7.54E-08	4.96E-08	2.80E-07	1.83E-12
	ECM_1 vs. ECM_{1000}		0.5	5.90E-08	7.94E-08	5.50E-08	3.24E-07	1.90E-12
	ECM_1 vs. ECM_{1000}		1	4.70E-08	6.85E-08	4.85E-08	3.00E-07	1.59E-12
	ECM_1 vs. ECM_{1000}		1.5	5.34E-08	8.33E-08	6.13E-08	4.02E-07	1.65E-12
	ECM_1 vs. ECM_{1000}		2	4.94E-08	8.09E-08	6.04E-08	4.29E-07	1.47E-12
MP2	ECM_1 vs. ECM_{1000}	0	0	2.39E-08	1.40E-07	1.15E-07	5.77E-07	1.75E-12
	ECM_1 vs. ECM_{1000}		0.5	2.16E-08	1.12E-07	8.99E-08	3.91E-07	2.31E-12
	ECM_1 vs. ECM_{1000}		1	2.11E-08	1.01E-07	8.02E-08	3.00E-07	3.18E-12
	ECM_1 vs. ECM_{1000}		1.5	2.05E-08	8.34E-08	6.42E-08	2.16E-07	3.33E-12
	ECM_1 vs. ECM_{1000}		2	2.30E-08	8.29E-08	6.18E-08	1.87E-07	4.30E-12
	ECM_1 vs. ECM_{1000}	1	0	9.13E-08	1.13E-07	7.68E-08	8.14E-07	1.14E-12
	ECM_1 vs. ECM_{1000}		0.5	1.05E-07	1.18E-07	7.82E-08	1.01E-06	1.04E-12
	ECM_1 vs. ECM_{1000}		1	8.89E-08	1.01E-07	6.87E-08	1.02E-06	7.12E-13
	ECM_1 vs. ECM_{1000}		1.5	1.03E-07	1.12E-07	7.77E-08	1.30E-06	7.21E-13
	ECM_1 vs. ECM_{1000}		2	9.83E-08	1.12E-07	7.88E-08	1.50E-06	5.75E-13
	ECM_1 vs. ECM_{1000}	2	0	8.14E-08	9.54E-08	6.01E-08	3.35E-07	2.78E-12
	ECM_1 vs. ECM_{1000}		0.5	1.02E-07	1.34E-07	8.99E-08	5.14E-07	2.92E-12
	ECM_1 vs. ECM_{1000}		1	8.20E-08	1.25E-07	9.01E-08	5.24E-07	2.24E-12
	ECM_1 vs. ECM_{1000}		1.5	7.36E-08	1.19E-07	8.77E-08	5.51E-07	2.19E-12
	ECM_1 vs. ECM_{1000}		2	6.81E-08	1.20E-07	9.13E-08	6.18E-07	1.97E-12
MP3	ECM_1 vs. ECM_{1000}	0	0	1.11E-08	3.98E-07	3.75E-07	1.24E-06	5.94E-12
	ECM_1 vs. ECM_{1000}		0.5	1.72E-08	4.15E-07	3.92E-07	1.01E-06	9.43E-12
	ECM_1 vs. ECM_{1000}		1	2.00E-08	3.41E-07	3.22E-07	6.96E-07	1.23E-11
	ECM_1 vs. ECM_{1000}		1.5	1.49E-08	2.11E-07	2.00E-07	3.56E-07	1.70E-11
	ECM_1 vs. ECM_{1000}		2	2.47E-08	2.77E-07	2.70E-07	3.84E-07	2.56E-11
	ECM_1 vs. ECM_{1000}	1	0	1.98E-07	2.60E-07	1.82E-07	1.80E-06	2.61E-12
	ECM_1 vs. ECM_{1000}		0.5	2.26E-07	2.55E-07	1.69E-07	2.05E-06	1.68E-12
	ECM_1 vs. ECM_{1000}		1	1.60E-03	1.22E-03	6.50E-04	1.22E-02	9.21E-04
	**ECM_{10} vs. ECM_{1000}**		**1**	**2.47E-07**	**2.68E-07**	**1.76E-07**	**2.68E-06**	**3.79E-13**

	ECM_1 vs. ECM_{1000}		1.5	2.84E-07	3.01E-07	2.02E-07	3.50E-06	1.11E-12
	ECM_1 vs. ECM_{1000}		2	1.20E-02	9.25E-03	3.93E-03	1.08E-01	3.13E-02
	**ECM_{10} vs. ECM_{1000}**		**2**	**2.44E-07**	**2.75E-07**	**1.95E-07**	**3.43E-06**	**7.26E-13**
	ECM_1 vs. ECM_{1000}	2	0	2.56E-07	2.95E-07	1.78E-07	9.27E-07	2.24E-11
	ECM_1 vs. ECM_{1000}		0.5	2.01E-07	3.13E-07	2.21E-07	9.85E-07	1.63E-11
	ECM_1 vs. ECM_{1000}		1	1.80E-07	3.23E-07	2.48E-07	1.11E-06	1.15E-11
	ECM_1 vs. ECM_{1000}		1.5	1.57E-07	3.30E-07	2.68E-07	1.29E-06	6.57E-12
	ECM_1 vs. ECM_{1000}		2	1.45E-07	3.15E-07	2.62E-07	1.37E-06	5.04E-12
MP4	ECM_1 vs. ECM_{1000}	0	0	2.43E-07	1.24E-06	1.25E-06	2.01E-06	5.10E-11
	ECM_1 vs. ECM_{1000}		0.5	1.86E-07	1.08E-06	1.12E-06	1.44E-06	7.66E-11
	ECM_1 vs. ECM_{1000}		1	1.69E-07	1.05E-06	1.07E-06	1.21E-06	6.63E-11
	ECM_1 vs. ECM_{1000}		1.5	1.54E-07	1.03E-06	1.06E-06	1.02E-06	8.65E-11
	ECM_1 vs. ECM_{1000}		2	1.24E-07	8.94E-07	9.18E-07	7.73E-07	7.99E-11
	ECM_1 vs. ECM_{1000}	1	0	2.70E-07	4.52E-07	3.43E-07	2.64E-06	5.11E-12
	ECM_1 vs. ECM_{1000}		0.5	3.57E-07	4.67E-07	3.29E-07	3.48E-06	3.33E-12
	ECM_1 vs. ECM_{1000}		1	3.66E-03	2.83E-03	1.68E-03	2.94E-02	2.53E-03
	**ECM_{10} vs. ECM_{1000}**		**1**	**4.20E-07**	**5.44E-07**	**3.81E-07**	**4.64E-06**	**3.83E-12**
	ECM_1 vs. ECM_{1000}		1.5	1.25E-02	9.42E-03	4.41E-03	1.09E-01	9.96E-03
	**ECM_{10} vs. ECM_{1000}**		**1.5**	**3.88E-07**	**5.31E-07**	**3.87E-07**	**4.85E-06**	**1.07E-11**
	ECM_1 vs. ECM_{1000}		2	3.34E-02	2.63E-02	1.18E-02	3.32E-01	5.94E-02
	**ECM_{10} vs. ECM_{1000}**		**2**	**1.47E-07**	**3.20E-07**	**2.83E-07**	**3.07E-06**	**1.51E-11**
	ECM_1 vs. ECM_{1000}	2	0	2.32E-07	1.17E-06	1.11E-06	1.55E-06	2.31E-10
	ECM_1 vs. ECM_{1000}		0.5	1.76E-07	9.57E-07	8.85E-07	1.58E-06	1.33E-10
	ECM_1 vs. ECM_{1000}		1	1.71E-07	9.99E-07	9.38E-07	2.03E-06	7.79E-11
	ECM_1 vs. ECM_{1000}		1.5	1.48E-07	9.17E-07	8.62E-07	2.17E-06	4.04E-11
	ECM_1 vs. ECM_{1000}		2	1.54E-07	9.52E-07	8.97E-07	2.70E-06	2.99E-11
MP5	ECM_1 vs. ECM_{1000}	0	0	2.72E-08	1.08E-07	8.73E-08	4.95E-07	1.79E-12
	ECM_1 vs. ECM_{1000}		0.5	2.83E-08	9.55E-08	7.54E-08	3.74E-07	2.16E-12
	ECM_1 vs. ECM_{1000}		1	3.07E-08	8.85E-08	6.82E-08	3.02E-07	2.23E-12
	ECM_1 vs. ECM_{1000}		1.5	3.33E-08	8.29E-08	6.24E-08	2.49E-07	3.29E-12
	ECM_1 vs. ECM_{1000}		2	2.25E-08	4.70E-08	3.39E-08	1.33E-07	3.02E-12
	ECM_1 vs. ECM_{1000}	1	0	8.78E-08	8.23E-08	5.44E-08	7.43E-07	9.50E-13
	ECM_1 vs. ECM_{1000}		0.5	1.20E-07	1.06E-07	7.01E-08	1.09E-06	8.78E-13
	ECM_1 vs. ECM_{1000}		1	1.31E-07	1.11E-07	7.13E-08	1.27E-06	8.32E-13
	ECM_1 vs. ECM_{1000}		1.5	1.12E-07	1.02E-07	6.97E-08	1.37E-06	6.75E-13
	ECM_1 vs. ECM_{1000}		2	2.90E-03	1.89E-03	9.26E-04	2.88E-02	4.51E-02
	**ECM_{10} vs. ECM_{1000}**		**2**	**1.54E-07**	**1.25E-07**	**7.83E-08**	**1.71E-06**	**3.23E-13**
	ECM_1 vs. ECM_{1000}	2	0	1.26E-07	1.05E-07	6.40E-08	4.60E-07	2.74E-12
	ECM_1 vs. ECM_{1000}		0.5	1.29E-07	1.23E-07	8.14E-08	5.64E-07	1.89E-12
	ECM_1 vs. ECM_{1000}		1	1.02E-07	1.08E-07	7.43E-08	5.41E-07	1.79E-12
	ECM_1 vs. ECM_{1000}		1.5	9.80E-08	1.17E-07	8.42E-08	6.25E-07	1.67E-12
	ECM_1 vs. ECM_{1000}		2	8.19E-08	1.09E-07	8.06E-08	6.57E-07	1.46E-12
MP6	ECM_1 vs. ECM_{1000}	0	0	4.14E-08	2.83E-07	2.75E-07	1.07E-06	4.38E-12
	ECM_1 vs. ECM_{1000}		0.5	5.11E-08	3.18E-07	3.14E-07	9.19E-07	6.24E-12

ECM_1 vs. ECM_{1000}		1	4.41E-08	2.60E-07	2.61E-07	6.21E-07	8.27E-12
ECM_1 vs. ECM_{1000}		1.5	3.53E-08	1.99E-07	2.06E-07	3.74E-07	1.47E-11
ECM_1 vs. ECM_{1000}		2	4.47E-08	2.29E-07	2.40E-07	3.57E-07	2.13E-11
ECM_1 vs. ECM_{1000}	1	0	1.63E-07	2.41E-07	1.69E-07	1.58E-06	2.48E-12
ECM_1 vs. ECM_{1000}		0.5	1.78E-07	2.42E-07	1.70E-07	1.82E-06	1.51E-12
ECM_1 vs. ECM_{1000}		1	2.30E-07	2.95E-07	1.95E-07	2.56E-06	1.22E-12
ECM_1 vs. ECM_{1000}		1.5	2.09E-07	2.53E-07	1.68E-07	2.57E-06	8.75E-13
ECM_1 vs. ECM_{1000}		2	1.20E-03	1.12E-03	5.08E-04	9.17E-03	1.22E-03
**ECM_{10} vs. ECM_{1000}**		**2**	**1.98E-07**	**2.63E-07**	**1.88E-07**	**3.22E-06**	**6.14E-13**
ECM_1 vs. ECM_{1000}	2	0	2.03E-07	2.93E-07	1.77E-07	8.42E-07	1.96E-11
ECM_1 vs. ECM_{1000}		0.5	1.82E-07	3.12E-07	2.23E-07	9.81E-07	2.28E-11
ECM_1 vs. ECM_{1000}		1	1.58E-07	3.09E-07	2.43E-07	1.08E-06	1.28E-11
ECM_1 vs. ECM_{1000}		1.5	1.51E-07	3.09E-07	2.54E-07	1.20E-06	7.11E-12
ECM_1 vs. ECM_{1000}		2	1.56E-07	3.37E-07	2.87E-07	1.53E-06	6.03E-12

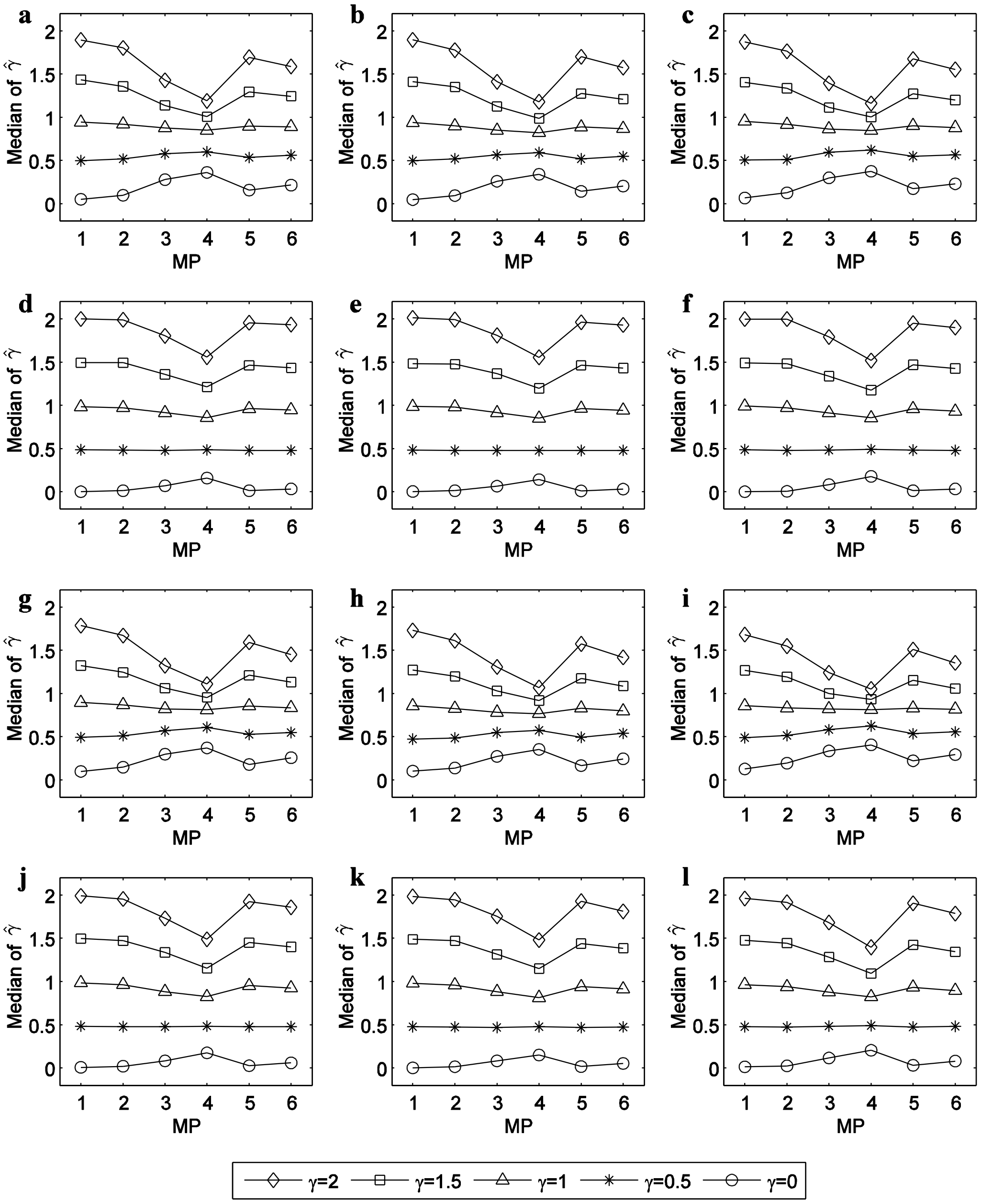

Fig. S1 Medians of point estimates of γ against MP with $\rho = 0$ for different p_m, p_f and values. The results are based on 10,000 replicates. **a** (p_m, p_f) = (0.30, 0.30) and λ_2 =1.5; **b** (p_m, p_f) = (0.25, 0.30) and $\lambda_2 = 1.5$; **c** (p_m, p_f) = (0.30, 0.25) and $\lambda_2 = 1.5$; **d** (p_m, p_f) = (0.30, 0.30) and $\lambda_2 = 2$; **e** (p_m, p_f) = (0.25, 0.30) and $\lambda_2 = 2$; **f** (p_m, p_f) = (0.30, 0.25) and $\lambda_2 = 2$; **g** (p_m, p_f) = (0.20, 0.20) and $\lambda_2 = 1.5$; **h** (p_m, p_f) = (0.15, 0.20) and $\lambda_2 = 1.5$; **i** (p_m, p_f) = (0.20, 0.15) and $\lambda_2 = 1.5$; **j** (p_m, p_f) = (0.20, 0.20) and $\lambda_2 = 2$; **k** (p_m, p_f) = (0.15, 0.20) and $\lambda_2 = 2$; **l** (p_m, p_f) = (0.20, 0.15) and $\lambda_2 = 2$

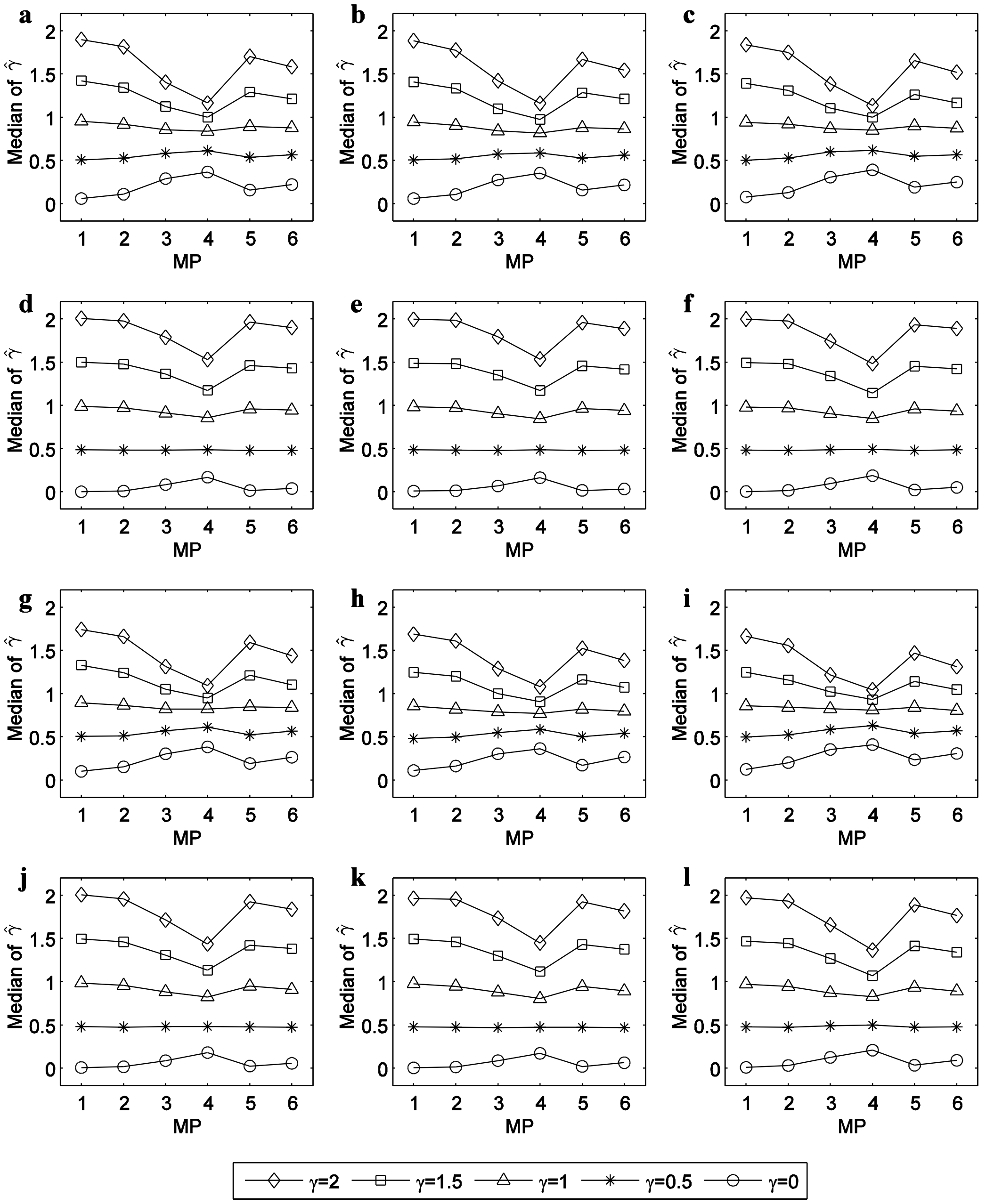

Fig. S2 Medians of point estimates of γ against MP with $\rho = 0.05$ for different p_m, p_f and λ_2 values. The results are based on 10,000 replicates. **a** (p_m, p_f) = (0.30, 0.30) and $\lambda_2 = 1.5$; **b** (p_m, p_f) = (0.25, 0.30) and $\lambda_2 = 1.5$; **c** (p_m, p_f) = (0.30, 0.25) and $\lambda_2 = 1.5$; **d** (p_m, p_f) = (0.30, 0.30) and $\lambda_2 = 2$; **e** (p_m, p_f) = (0.25, 0.30) and $\lambda_2 = 2$; **f** (p_m, p_f) = (0.30, 0.25) and $\lambda_2 = 2$; **g** (p_m, p_f) = (0.20, 0.20) and $\lambda_2 = 1.5$; **h** (p_m, p_f) = (0.15, 0.20) and $\lambda_2 = 1.5$; **i** (p_m, p_f) = (0.20, 0.15) and $\lambda_2 = 1.5$; **j** (p_m, p_f) = (0.20, 0.20) and $\lambda_2 = 2$; **k** (p_m, p_f) = (0.15, 0.20) and $\lambda_2 = 2$; **l** (p_m, p_f) = (0.20, 0.15) and $\lambda_2 = 2$

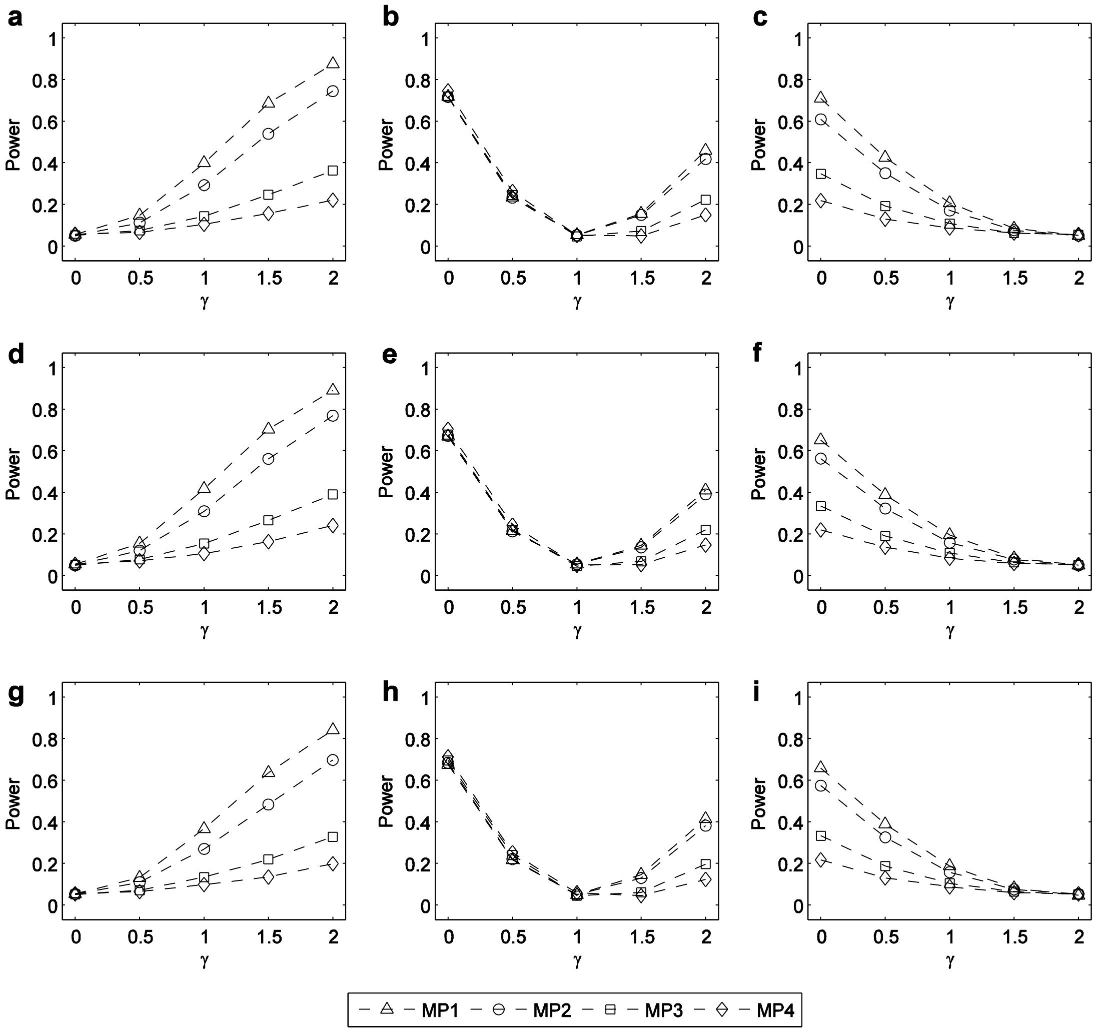

Fig. S3 Estimated powers of LRT against γ under MP1–MP4 with ρ = 0.05 and λ_2 = 1.5. The results are based on 10,000 replicates and 5% significance level. **a** (p_m, p_f) = (0.30, 0.30) and $\gamma_0 = 0$; **b** (p_m, p_f) = (0.30, 0.30) and $\gamma_0 = 1$; **c** (p_m, p_f) = (0.30, 0.30) and $\gamma_0 = 2$; **d** (p_m, p_f) = (0.25, 0.30) and $\gamma_0 = 0$; **e** (p_m, p_f) = (0. 25, 0.30) and $\gamma_0 = 1$; **f** (p_m, p_f) = (0.25, 0.30) and $\gamma_0 = 2$; **g** (p_m, p_f) = (0.30, 0.25) and $\gamma_0 = 0$; **h** (p_m, p_f) = (0.30, 0.25) and $\gamma_0 = 1$; **i** (p_m, p_f) = (0.30, 0.25) and $\gamma_0 = 2$. Note that $\gamma_0 = 0$, 1 and 2 represent XCI skewing completely against mutant allele, random XCI and XCI skewing completely toward mutant allele, respectively.

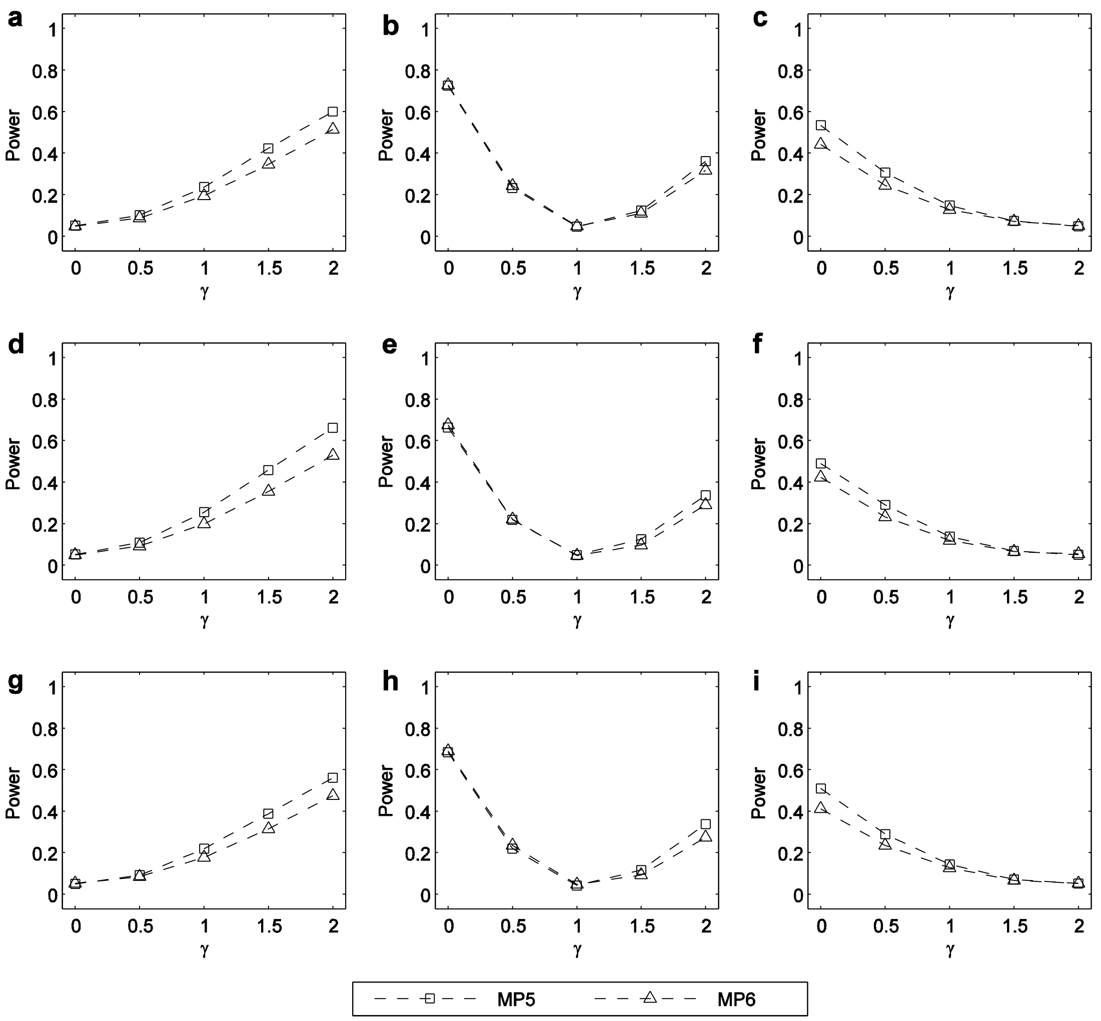

Fig. S4 Estimated powers of LRT against γ under MP5 and MP6 with $\rho = 0.05$ and $\lambda_2 = 1.5$. The results are based on 10,000 replicates and 5% significance level. **a** (p_m, p_f) = (0.30, 0.30) and $\gamma_0 = 0$; **b** (p_m, p_f) = (0.30, 0.30) and $\gamma_0 = 1$; **c** (p_m, p_f) = (0.30, 0.30) and $\gamma_0 = 2$; **d** (p_m, p_f) = (0.25, 0.30) and $\gamma_0 = 0$; **e** (p_m, p_f) = (0. 25, 0.30) and $\gamma_0 = 1$; **f** (p_m, p_f) = (0.25, 0.30) and $\gamma_0 = 2$; **g** (p_m, p_f) = (0.30, 0.25) and $\gamma_0 = 0$; **h** (p_m, p_f) = (0.30, 0.25) and $\gamma_0 = 1$; **i** (p_m, p_f) = (0.30, 0.25) and $\gamma_0 = 2$. Note that $\gamma_0 = 0$, 1 and 2 represent XCI skewing completely against mutant allele, random XCI and XCI skewing completely toward mutant allele, respectively.

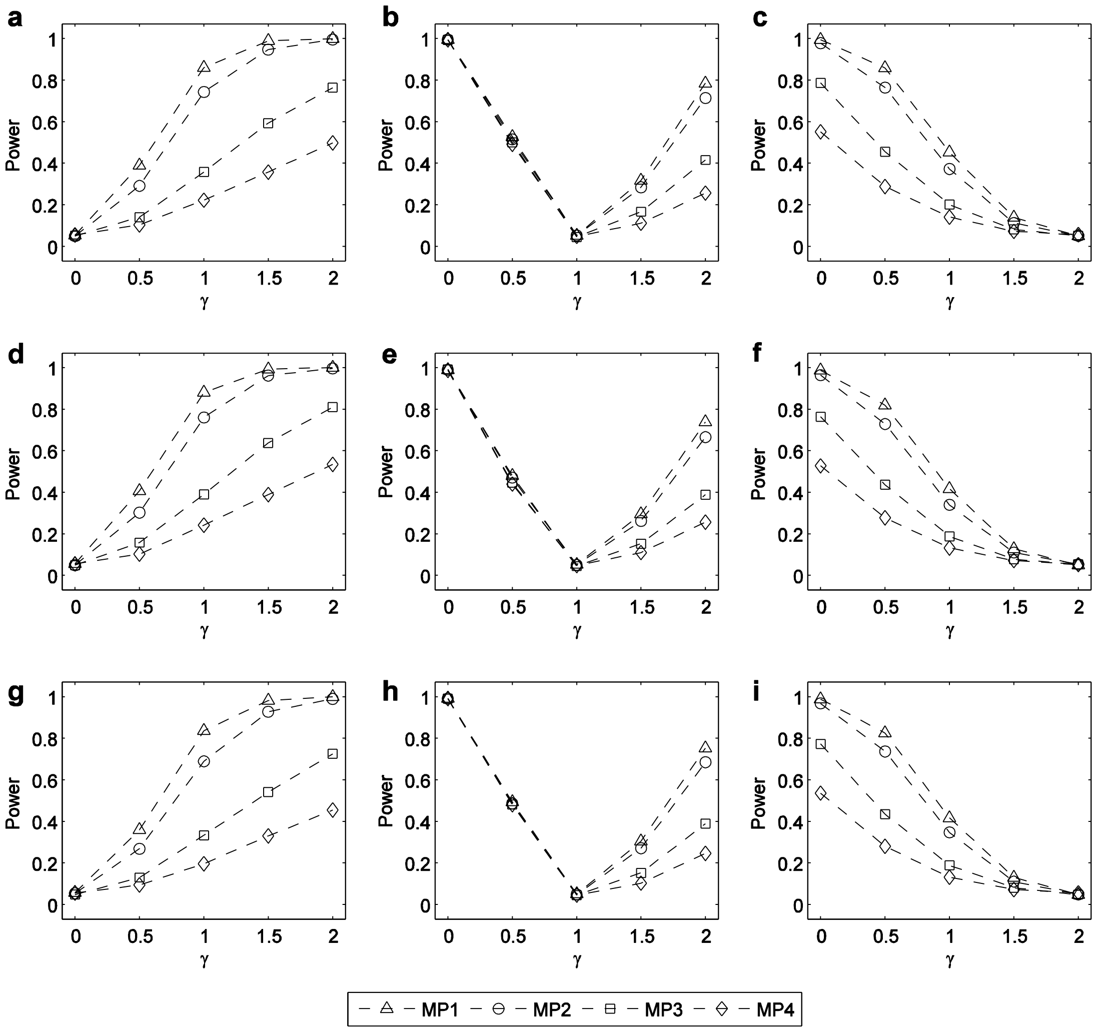

Fig. S5 Estimated powers of LRT against γ under MP1–MP4 with $\rho = 0.05$ and $\lambda_2 = 2$. The results are based on 10,000 replicates and 5% significance level. **a** (p_m, p_f) = (0.30, 0.30) and $\gamma_0 = 0$; **b** (p_m, p_f) = (0.30, 0.30) and $\gamma_0 = 1$; **c** (p_m, p_f) = (0.30, 0.30) and $\gamma_0 = 2$; **d** (p_m, p_f) = (0.25, 0.30) and $\gamma_0 = 0$; **e** (p_m, p_f) = (0. 25, 0.30) and $\gamma_0 = 1$; **f** (p_m, p_f) = (0.25, 0.30) and $\gamma_0 = 2$; **g** (p_m, p_f) = (0.30, 0.25) and $\gamma_0 = 0$; **h** (p_m, p_f) = (0.30, 0.25) and $\gamma_0 = 1$; **i** (p_m, p_f) = (0.30, 0.25) and $\gamma_0 = 2$. Note that $\gamma_0 = 0$, 1 and 2 represent XCI skewing completely against mutant allele, random XCI and XCI skewing completely toward mutant allele, respectively.

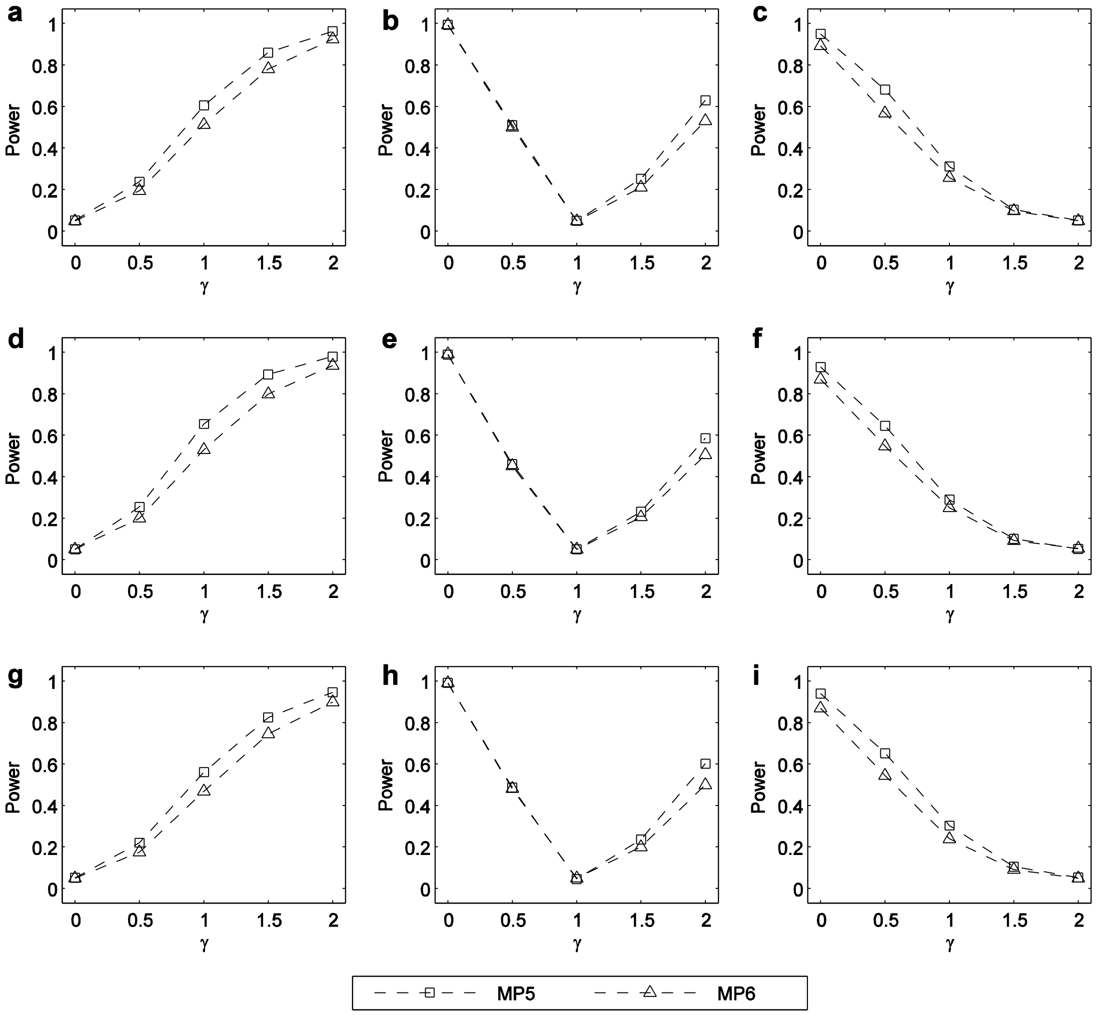

Fig. S6 Estimated powers of LRT against γ under MP5 and MP6 with $\rho = 0.05$ and $\lambda_2 = 2$. The results are based on 10,000 replicates and 5% significance level. **a** (p_m, p_f) = (0.30, 0.30) and $\gamma_0 = 0$; **b** (p_m, p_f) = (0.30, 0.30) and $\gamma_0 = 1$; **c** (p_m, p_f) = (0.30, 0.30) and $\gamma_0 = 2$; **d** (p_m, p_f) = (0.25, 0.30) and $\gamma_0 = 0$; **e** (p_m, p_f) = (0. 25, 0.30) and $\gamma_0 = 1$; **f** (p_m, p_f) = (0.25, 0.30) and $\gamma_0 = 2$; **g** (p_m, p_f) = (0.30, 0.25) and $\gamma_0 = 0$; **h** (p_m, p_f) = (0.30, 0.25) and $\gamma_0 = 1$; **i** (p_m, p_f) = (0.30, 0.25) and $\gamma_0 = 2$. Note that $\gamma_0 = 0$, 1 and 2 represent XCI skewing completely against mutant allele, random XCI and XCI skewing completely toward mutant allele, respectively.

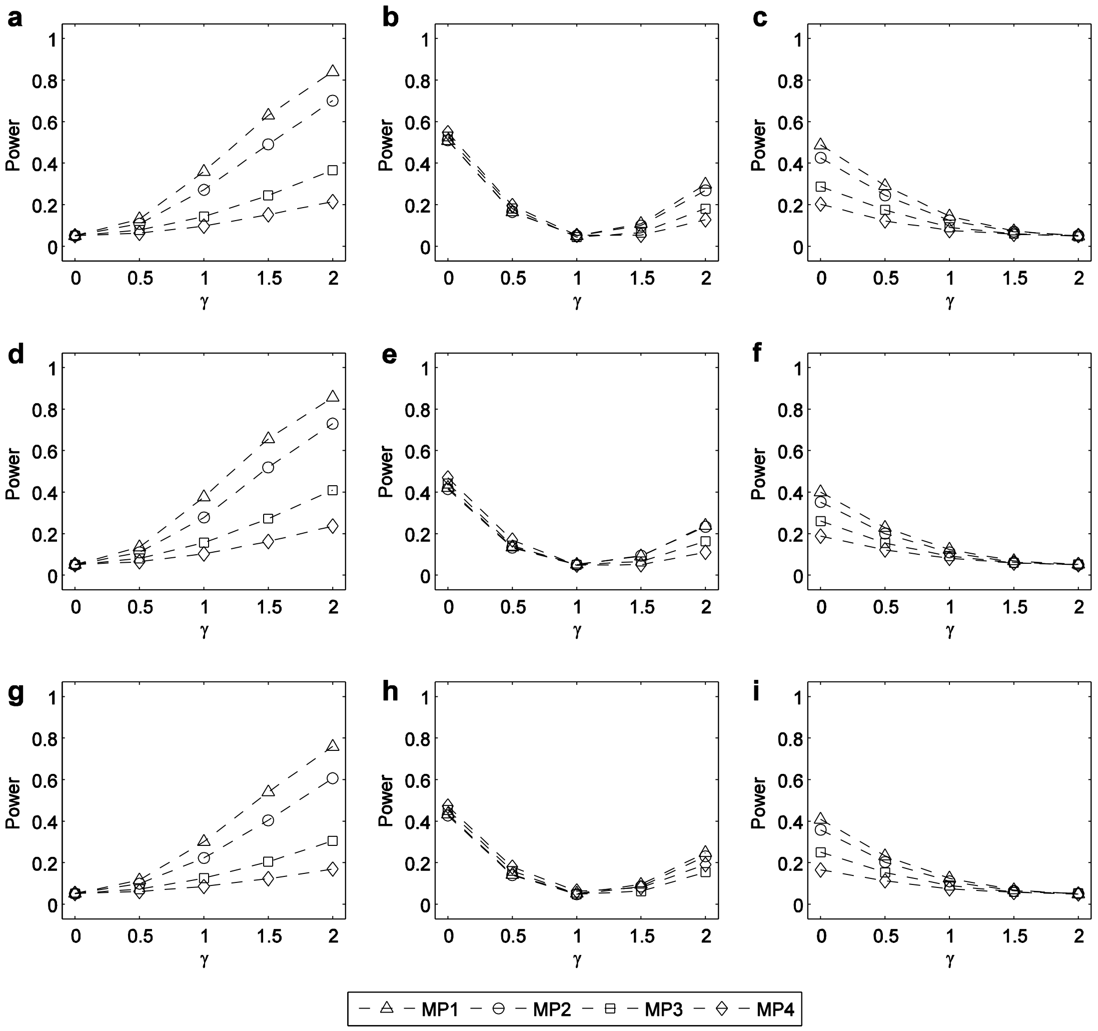

Fig. S7 Estimated powers of LRT against γ under MP1–MP4 with $\rho = 0$ and $\lambda_2 = 1.5$. The results are based on 10,000 replicates and 5% significance level. **a** (p_m, p_f) = (0.20, 0.20) and $\gamma_0 = 0$; **b** (p_m, p_f) = (0.20, 0.20) and $\gamma_0 = 1$; **c** (p_m, p_f) = (0.20, 0.20) and $\gamma_0 = 2$; **d** (p_m, p_f) = (0.15, 0.20) and $\gamma_0 = 0$; **e** (p_m, p_f) = (0.15, 0.20) and $\gamma_0 = 1$; **f** (p_m, p_f) = (0.15, 0.20) and $\gamma_0 = 2$; **g** (p_m, p_f) = (0.20, 0.15) and $\gamma_0 = 0$; **h** (p_m, p_f) = (0.20, 0.15) and $\gamma_0 = 1$; **i** (p_m, p_f) = (0.20, 0.15) and $\gamma_0 = 2$. Note that $\gamma_0 = 0$, 1 and 2 represent XCI skewing completely against mutant allele, random XCI and XCI skewing completely toward mutant allele, respectively.

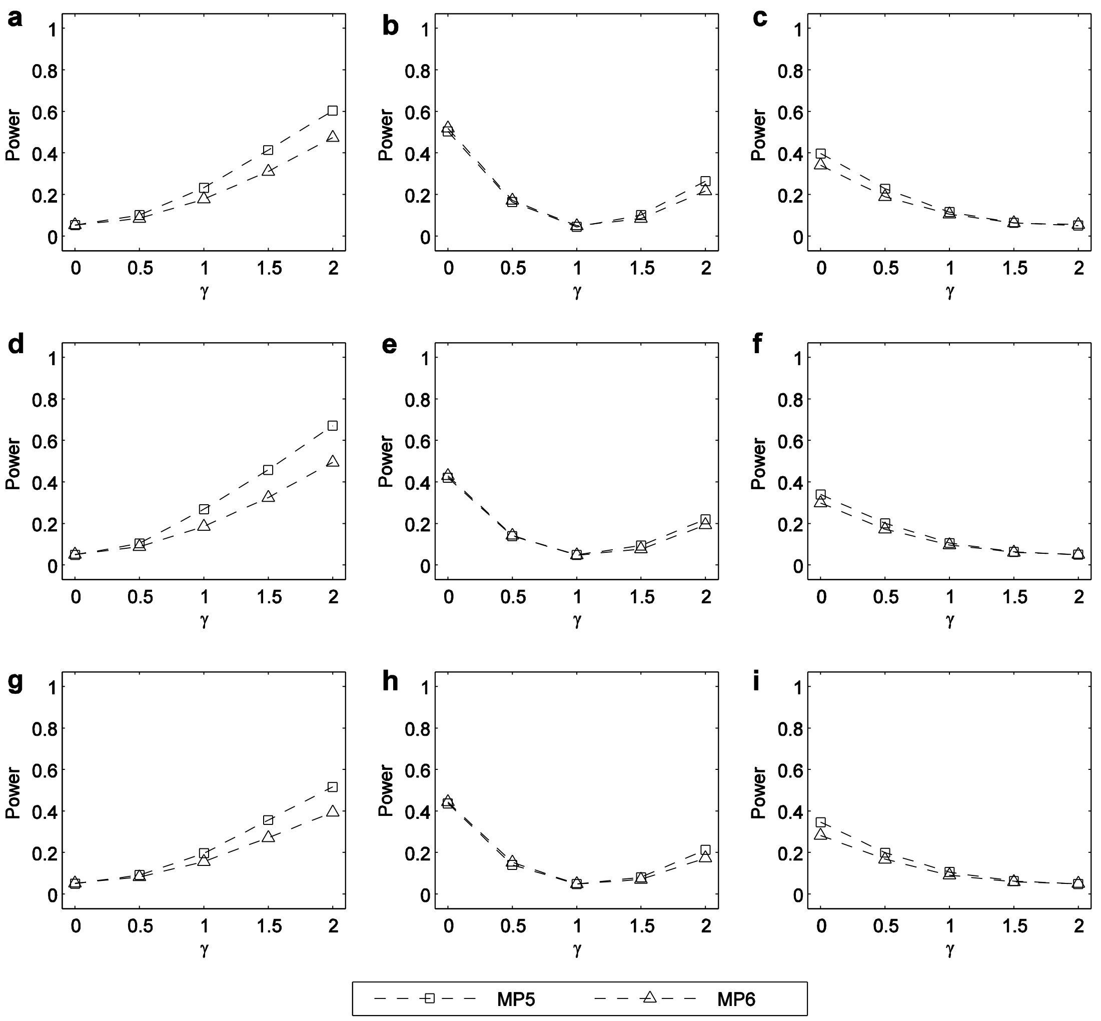

Fig. S8 Estimated powers of LRT against γ under MP5 and MP6 with $\rho = 0$ and $\lambda_2 =$ 1.5. The results are based on 10,000 replicates and 5% significance level. **a** (p_m, p_f) = (0.20, 0.20) and $\gamma_0 = 0$; **b** (p_m, p_f) = (0.20, 0.20) and $\gamma_0 = 1$; **c** (p_m, p_f) = (0.20, 0.20) and $\gamma_0 = 2$; **d** (p_m, p_f) = (0.15, 0.20) and $\gamma_0 = 0$; **e** (p_m, p_f) = (0.15, 0.20) and $\gamma_0 = 1$; **f** (p_m, p_f) = (0.15, 0.20) and $\gamma_0 = 2$; **g** (p_m, p_f) = (0.20, 0.15) and $\gamma_0 = 0$; **h** (p_m, p_f) = (0.20, 0.15) and $\gamma_0 = 1$; **i** (p_m, p_f) = (0.20, 0.15) and $\gamma_0 = 2$. Note that $\gamma_0 = 0$, 1 and 2 represent XCI skewing completely against mutant allele, random XCI and XCI skewing completely toward mutant allele, respectively.

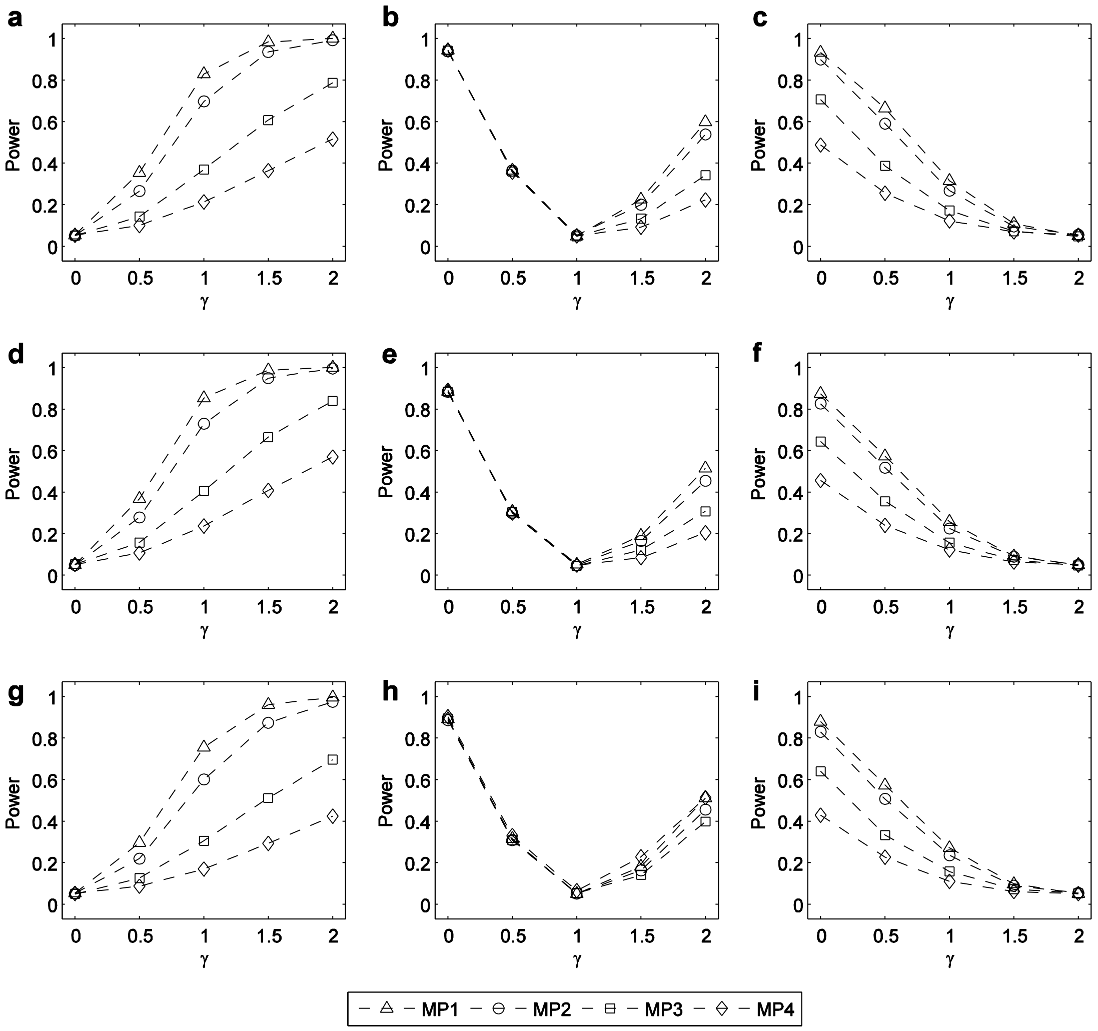

Fig. S9 Estimated powers of LRT against γ under MP1–MP4 with $\rho = 0$ and $\lambda_2 = 2$. The results are based on 10,000 replicates and 5% significance level. **a** (p_m, p_f) = (0.20, 0.20) and $\gamma_0 = 0$; **b** (p_m, p_f) = (0.20, 0.20) and $\gamma_0 = 1$; **c** (p_m, p_f) = (0.20, 0.20) and $\gamma_0 = 2$; **d** (p_m, p_f) = (0.15, 0.20) and $\gamma_0 = 0$; **e** (p_m, p_f) = (0.15, 0.20) and $\gamma_0 = 1$; **f** (p_m, p_f) = (0.15, 0.20) and $\gamma_0 = 2$; **g** (p_m, p_f) = (0.20, 0.15) and $\gamma_0 = 0$; **h** (p_m, p_f) = (0.20, 0.15) and $\gamma_0 = 1$; **i** (p_m, p_f) = (0.20, 0.15) and $\gamma_0 = 2$. Note that $\gamma_0 = 0$, 1 and 2 represent XCI skewing completely against mutant allele, random XCI and XCI skewing completely toward mutant allele, respectively.

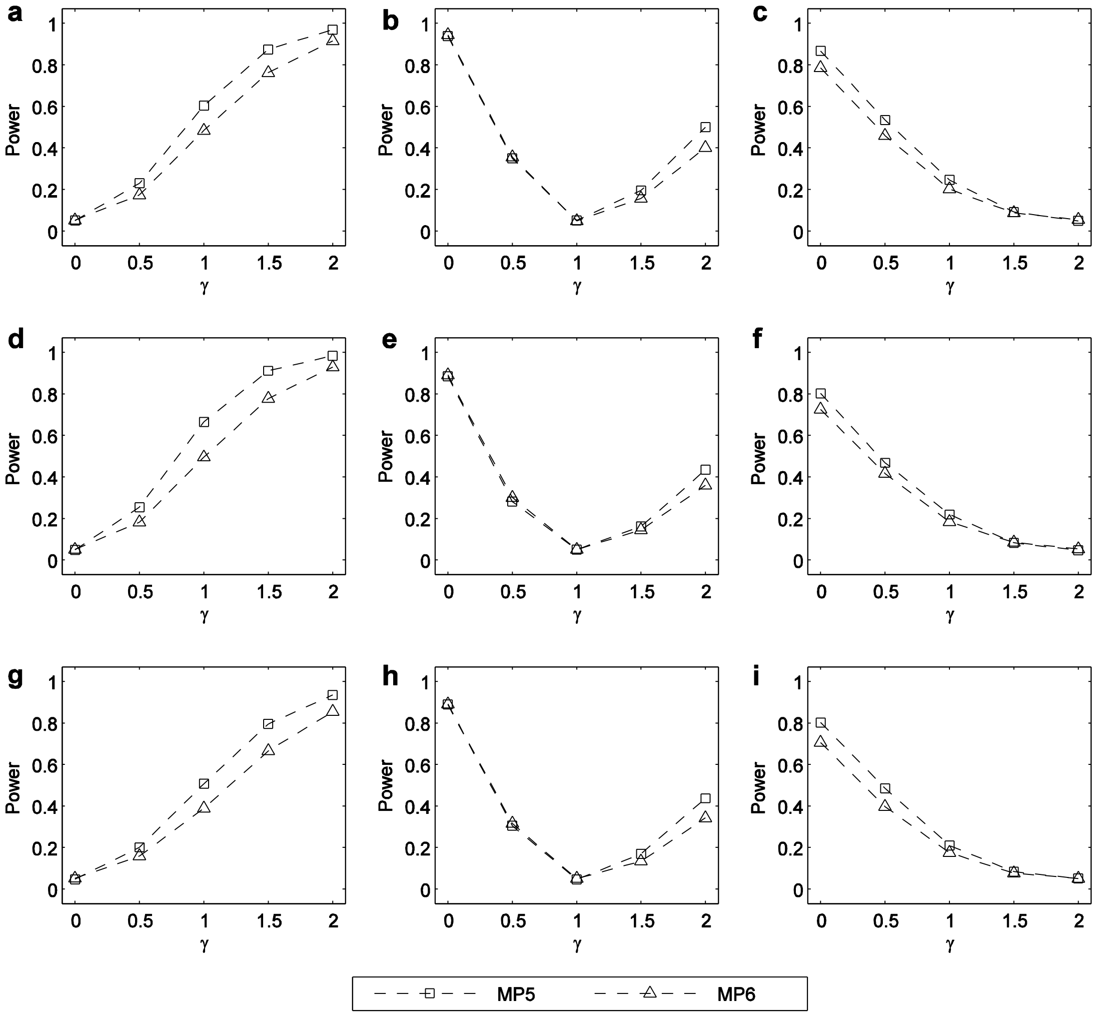

Fig. S10 Estimated powers of LRT against γ under MP5 and MP6 with $\rho = 0$ and $\lambda_2 = 2$. The results are based on 10,000 replicates and 5% significance level. **a** (p_m, p_f) = (0.20, 0.20) and $\gamma_0 = 0$; **b** (p_m, p_f) = (0.20, 0.20) and $\gamma_0 = 1$; **c** (p_m, p_f) = (0.20, 0.20) and $\gamma_0 = 2$; **d** (p_m, p_f) = (0.15, 0.20) and $\gamma_0 = 0$; **e** (p_m, p_f) = (0.15, 0.20) and $\gamma_0 = 1$; **f** (p_m, p_f) = (0.15, 0.20) and $\gamma_0 = 2$; **g** (p_m, p_f) = (0.20, 0.15) and $\gamma_0 = 0$; **h** (p_m, p_f) = (0.20, 0.15) and $\gamma_0 = 1$; **i** (p_m, p_f) = (0.20, 0.15) and $\gamma_0 = 2$. Note that $\gamma_0 = 0$, 1 and 2 represent XCI skewing completely against mutant allele, random XCI and XCI skewing completely toward mutant allele, respectively.

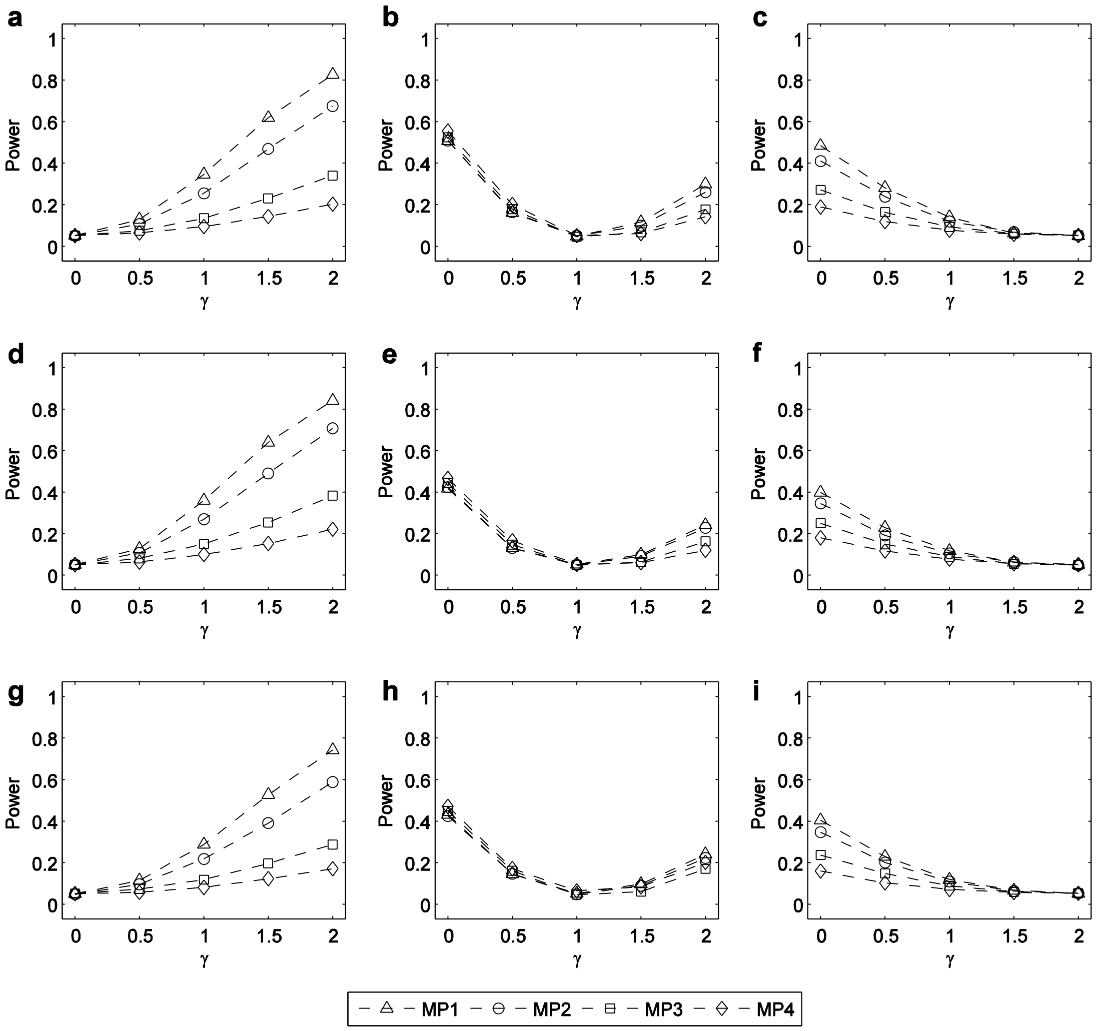

Fig. S11 Estimated powers of LRT against γ under MP1–MP4 with $\rho = 0.05$ and $\lambda_2 = 1.5$. The results are based on 10,000 replicates and 5% significance level. **a** (p_m, p_f) = (0.20, 0.20) and $\gamma_0 = 0$; **b** (p_m, p_f) = (0.20, 0.20) and $\gamma_0 = 1$; **c** (p_m, p_f) = (0.20, 0.20) and $\gamma_0 = 2$; **d** (p_m, p_f) = (0.15, 0.20) and $\gamma_0 = 0$; **e** (p_m, p_f) = (0.15, 0.20) and $\gamma_0 = 1$; **f** (p_m, p_f) = (0.15, 0.20) and $\gamma_0 = 2$; **g** (p_m, p_f) = (0.20, 0.15) and $\gamma_0 = 0$; **h** (p_m, p_f) = (0.20, 0.15) and $\gamma_0 = 1$; **i** (p_m, p_f) = (0.20, 0.15) and $\gamma_0 = 2$. Note that $\gamma_0 = 0$, 1 and 2 represent XCI skewing completely against mutant allele, random XCI and XCI skewing completely toward mutant allele, respectively.

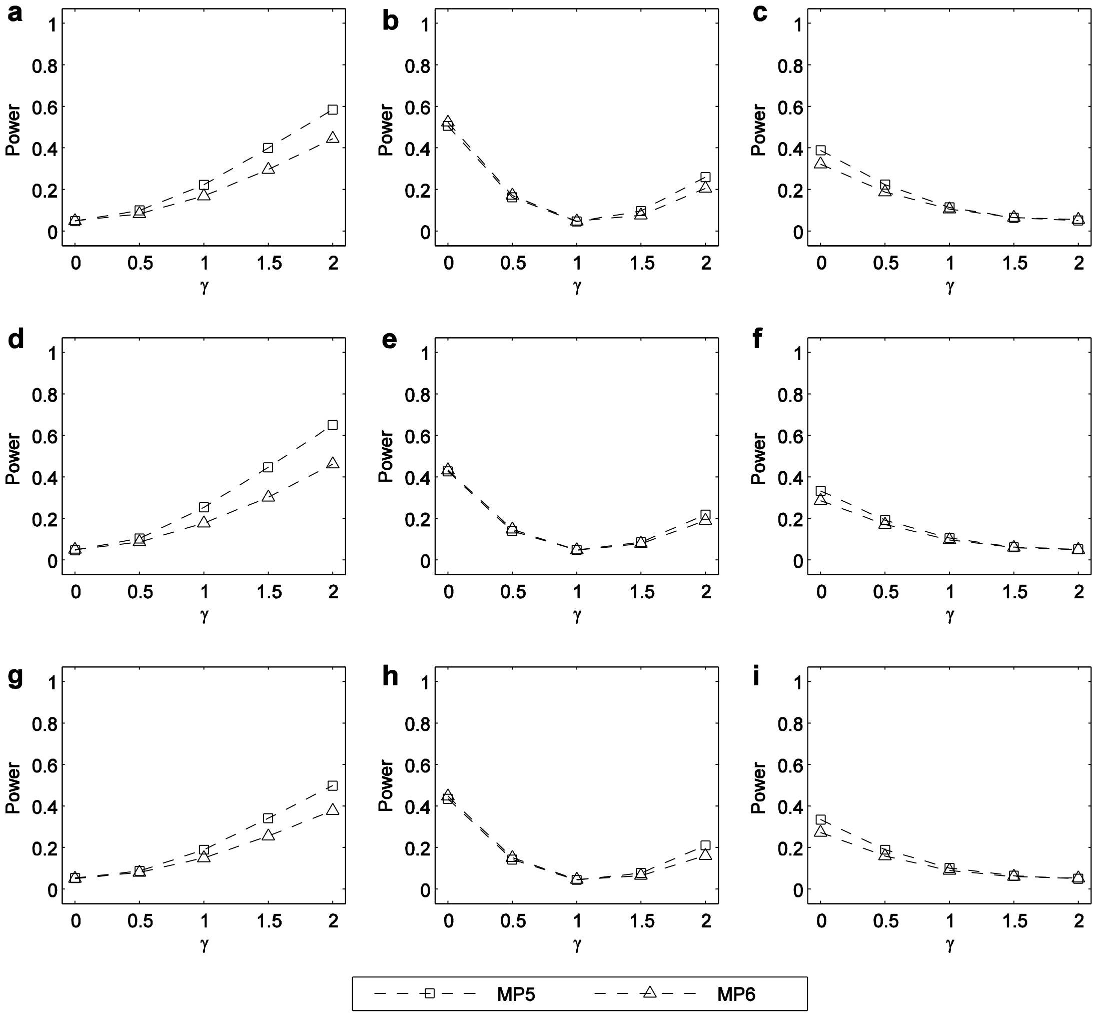

Fig. S12 Estimated powers of LRT against γ under MP5 and MP6 with $\rho = 0.05$ and $\lambda_2 = 1.5$. The results are based on 10,000 replicates and 5% significance level. **a** (p_m, p_f) = (0.20, 0.20) and $\gamma_0 = 0$; **b** (p_m, p_f) = (0.20, 0.20) and $\gamma_0 = 1$; **c** (p_m, p_f) = (0.20, 0.20) and $\gamma_0 = 2$; **d** (p_m, p_f) = (0.15, 0.20) and $\gamma_0 = 0$; **e** (p_m, p_f) = (0.15, 0.20) and $\gamma_0 = 1$; **f** (p_m, p_f) = (0.15, 0.20) and $\gamma_0 = 2$; **g** (p_m, p_f) = (0.20, 0.15) and $\gamma_0 = 0$; **h** (p_m, p_f) = (0.20, 0.15) and $\gamma_0 = 1$; **i** (p_m, p_f) = (0.20, 0.15) and $\gamma_0 = 2$. Note that $\gamma_0 = 0$, 1 and 2 represent XCI skewing completely against mutant allele, random XCI and XCI skewing completely toward mutant allele, respectively.

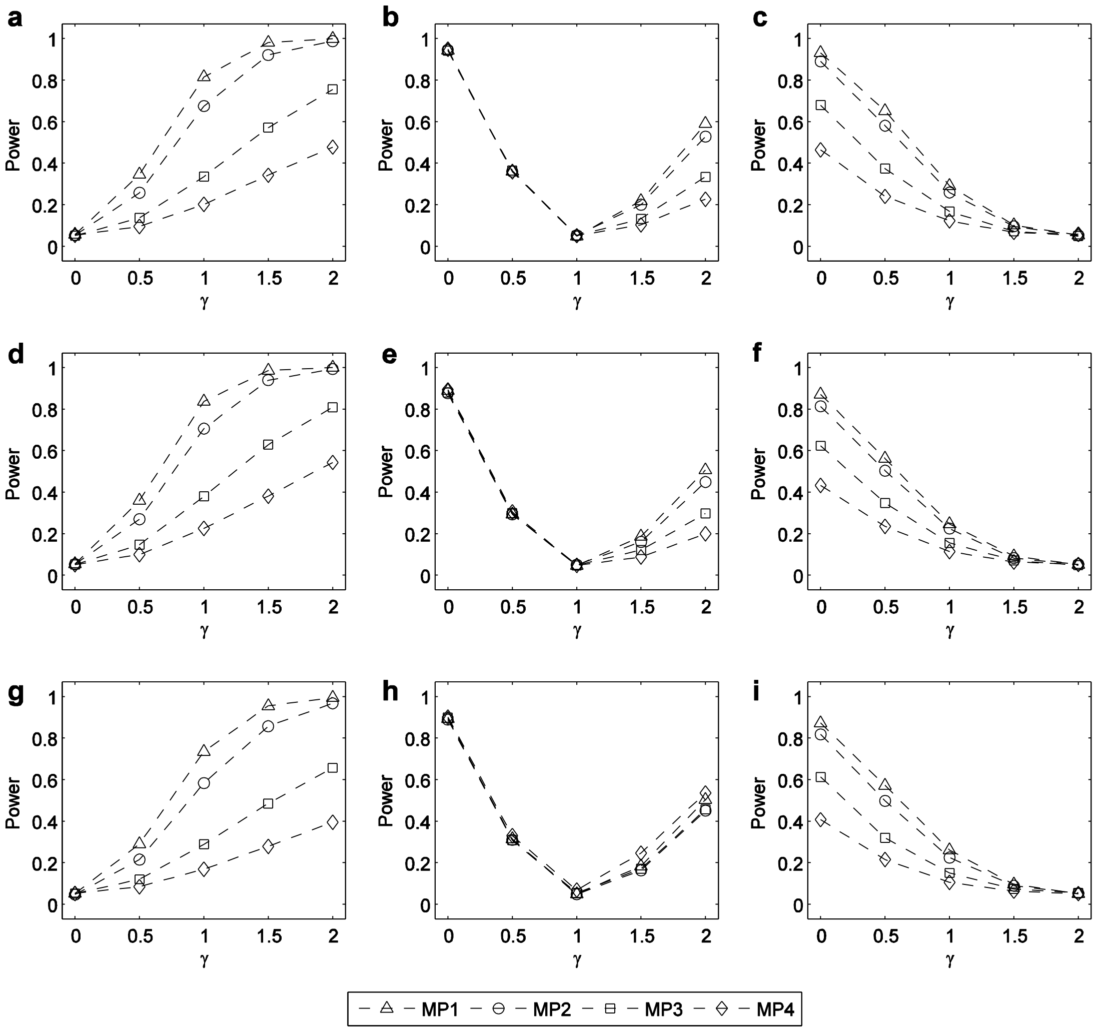

Fig. S13 Estimated powers of LRT against γ under MP1–MP4 with $\rho = 0.05$ and $\lambda_2 = 2$. The results are based on 10,000 replicates and 5% significance level. **a** (p_m, p_f) = (0.20, 0.20) and $\gamma_0 = 0$; **b** (p_m, p_f) = (0.20, 0.20) and $\gamma_0 = 1$; **c** (p_m, p_f) = (0.20, 0.20) and $\gamma_0 = 2$; **d** (p_m, p_f) = (0.15, 0.20) and $\gamma_0 = 0$; **e** (p_m, p_f) = (0.15, 0.20) and $\gamma_0 = 1$; **f** (p_m, p_f) = (0.15, 0.20) and $\gamma_0 = 2$; **g** (p_m, p_f) = (0.20, 0.15) and $\gamma_0 = 0$; **h** (p_m, p_f) = (0.20, 0.15) and $\gamma_0 = 1$; **i** (p_m, p_f) = (0.20, 0.15) and $\gamma_0 = 2$. Note that $\gamma_0 = 0$, 1 and 2 represent XCI skewing completely against mutant allele, random XCI and XCI skewing completely toward mutant allele, respectively.

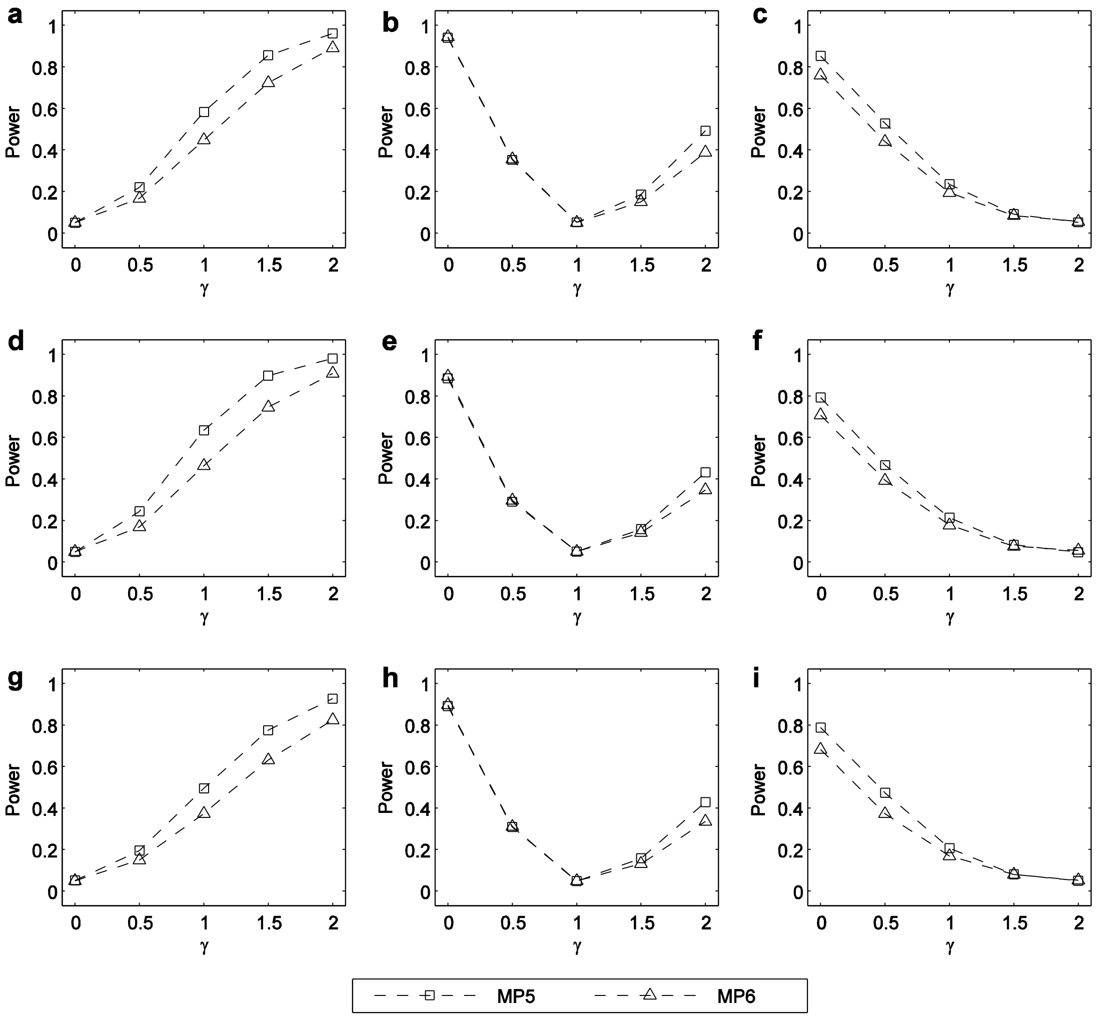

Fig. S14 Estimated powers of LRT against γ under MP5 and MP6 with $\rho = 0.05$ and $\lambda_2 = 2$. The results are based on 10,000 replicates and 5% significance level. **a** (p_m, p_f) = (0.20, 0.20) and $\gamma_0 = 0$; **b** (p_m, p_f) = (0.20, 0.20) and $\gamma_0 = 1$; **c** (p_m, p_f) = (0.20, 0.20) and $\gamma_0 = 2$; **d** (p_m, p_f) = (0.15, 0.20) and $\gamma_0 = 0$; **e** (p_m, p_f) = (0.15, 0.20) and $\gamma_0 = 1$; **f** (p_m, p_f) = (0.15, 0.20) and $\gamma_0 = 2$; **g** (p_m, p_f) = (0.20, 0.15) and $\gamma_0 = 0$; **h** (p_m, p_f) = (0.20, 0.15) and $\gamma_0 = 1$; **i** (p_m, p_f) = (0.20, 0.15) and $\gamma_0 = 2$. Note that $\gamma_0 = 0$, 1 and 2 represent XCI skewing completely against mutant allele, random XCI and XCI skewing completely toward mutant allele, respectively.
